# c-Met-targeted NIR-II imaging for precision management of oral squamous cell carcinoma and premalignant lesions

**DOI:** 10.7150/thno.132350

**Published:** 2026-05-29

**Authors:** Zhipeng Xia, Aiyan Ji, Qifan Ma, Yonghao Li, Hongyue Lou, Kun Qian, Jie Tian, Ying Yuan, Zhen Cheng, Xiaofeng Tao

**Affiliations:** 1 Department of Radiology, Shanghai Ninth People’s Hospital, Shanghai Jiao Tong University School of Medicine, Shanghai, 200011, China.; 2 State Key Laboratory of Drug Research, Molecular Imaging Center, Shanghai Institute of Materia Medica, Chinese Academy of Sciences, Shanghai, 201203, China.; 3 CAS Key Laboratory of Molecular Imaging, Beijing Key Laboratory of Molecular Imaging, Institute of Automation, Chinese Academy of Sciences, Beijing, 100190, China.; 4 University of Chinese Academy of Sciences, No.19A Yuquan Road, Beijing, 100049, China.

**Keywords:** oral squamous cell carcinoma, premalignant lesion, lymph node metastasis, near-infrared window II (NIR-II) fluorescence imaging, surgical navigation

## Abstract

**Background:**

Poor outcomes in oral squamous cell carcinoma (OSCC) are closely linked to delayed diagnosis, positive surgical margins and occult lymph node metastasis. A unified strategy for premalignant lesion screening, primary tumor delineation and metastatic lymph node detection remains lacking.

**Methods:**

We evaluated c-Met expression in human oral lesions, OSCC tissues and metastatic lymph nodes to establish its suitability as an imaging target. On this basis, we developed IR788-Crizotinib, a c-Met-targeted near-infrared window-II (NIR-II) fluorescent probe, and evaluated its imaging performance in subcutaneous xenograft, 4-NQO-induced oral lesion, orthotopic tongue OSCC and lymph node metastasis mouse models.

**Results:**

c-Met expression increased progressively during oral lesion progression and remained high in metastatic tumor deposits within lymph nodes. IR788-Crizotinib identified high-risk lesions with 100% sensitivity and 96.77% accuracy, including severe dysplasia lesions as small as 300 μm and microinvasive OSCC lesions as small as 286 μm. In fluorescence-guided surgery models, the probe identified multifocal tumors and detected residual foci as small as 1 mm. In cervical lymph nodes, it detected micro-metastatic foci as small as 342 μm, with 100% sensitivity.

**Conclusions:**

c-Met-targeted NIR-II imaging provides an integrated visualization strategy for premalignant lesion screening, primary tumor delineation and metastatic lymph node detection in OSCC, with translational potential for precision management of oral cancer.

## Introduction

Oral squamous cell carcinoma (OSCC) accounts for more than 90% of oral malignancies and remains a major global health burden, with an estimated 389,482 new cases and 188,230 deaths worldwide in 2022 1, 2. Despite advances in surgery, radiotherapy, chemotherapy, targeted therapy and immunotherapy, the 5-year overall survival rate of OSCC remains approximately 50% 3. This poor outcome is largely driven by three interrelated clinical challenges. First, early detection remains inadequate. Oral epithelial dysplasia (OED) is the major premalignant precursor of OSCC, and its risk of malignant transformation increases with dysplasia severity, with reported rates ranging from 6% to 40% 4-6. Timely intervention is therefore essential for patients with severe dysplasia and OSCC 7, 8. However, the diagnosis of OED and OSCC still relies heavily on histopathological biopsy, whereas subtle molecular and cytological abnormalities are often not apparent on routine visual examination, making optimal biopsy-site selection difficult. Repeated biopsies during long-term surveillance can further lead to diagnostic delay, reduced patient compliance and increased healthcare burden, contributing to the fact that nearly 60% of OSCC cases are diagnosed at advanced stages 9, 10. Second, intraoperative delineation of tumor extent remains challenging, resulting in positive surgical margins in approximately 40% of patients 11, 12. Positive margins are strongly associated with increased risks of local recurrence and mortality. Third, occult lymph node metastasis is common. Cervical lymph node metastasis occurs in about 40% of OSCC cases, and 15%-34% of these are occult 13. Although neck dissection remains the standard treatment for clinically node-positive disease, inaccurate staging may lead to incomplete removal of metastatic nodes, whereas in early-stage clinically node-negative disease, elective neck dissection may expose patients without pathological nodal involvement to unnecessary surgery, thereby creating parallel risks of undertreatment and overtreatment 13, 14. Together, these limitations underscore the need for an imaging strategy that can simultaneously support early lesion detection, primary tumor delineation and nodal staging in OSCC.

Fluorescence imaging has emerged as a powerful approach for cancer detection, staging, intraoperative guidance and postoperative assessment because of its rapid response, high sensitivity and favorable spatiotemporal resolution. Head and neck tumors, particularly oral cancers, are especially amenable to optical imaging because of their relatively superficial anatomical location 15-17. A variety of fluorescent probes have therefore been developed for early oral cancer detection, intraoperative margin assessment and sentinel lymph node mapping 5, 18-20. However, most of these strategies remain confined to the first near-infrared window (NIR-I, 700-900 nm), where tissue scattering and autofluorescence limit imaging contrast and penetration depth. Moreover, most currently available fluorescent probes are tailored to isolated clinical tasks, and their performance has rarely been evaluated in an integrated manner across the major stages of OSCC management, from premalignant lesion screening to preoperative assessment and intraoperative guidance. By contrast, imaging in the second near-infrared window (NIR-II, 1000-1700 nm) reduces light scattering and tissue autofluorescence, thereby improving signal-to-noise ratio, spatial resolution and imaging depth. These features make NIR-II imaging particularly attractive for visualizing both superficial oral lesions and cervical lymph nodes, and support its potential for clinical translation 21, 22.

Despite these advantages, the application of NIR-II imaging in OSCC has remained limited, in part because of the lack of highly specific probes with suitable *in vivo* imaging performance. The cellular mesenchymal-epithelial transition factor (c-Met), a receptor tyrosine kinase activated by hepatocyte growth factor (HGF), is overexpressed in more than 80% of OSCCs, whereas its expression in normal oral mucosa is limited 23, 24. Importantly, c-Met signaling is closely associated with tumor invasion and lymph node metastasis, making it a compelling target for monitoring OSCC initiation, progression and dissemination 24-26.

**Figure 1:**
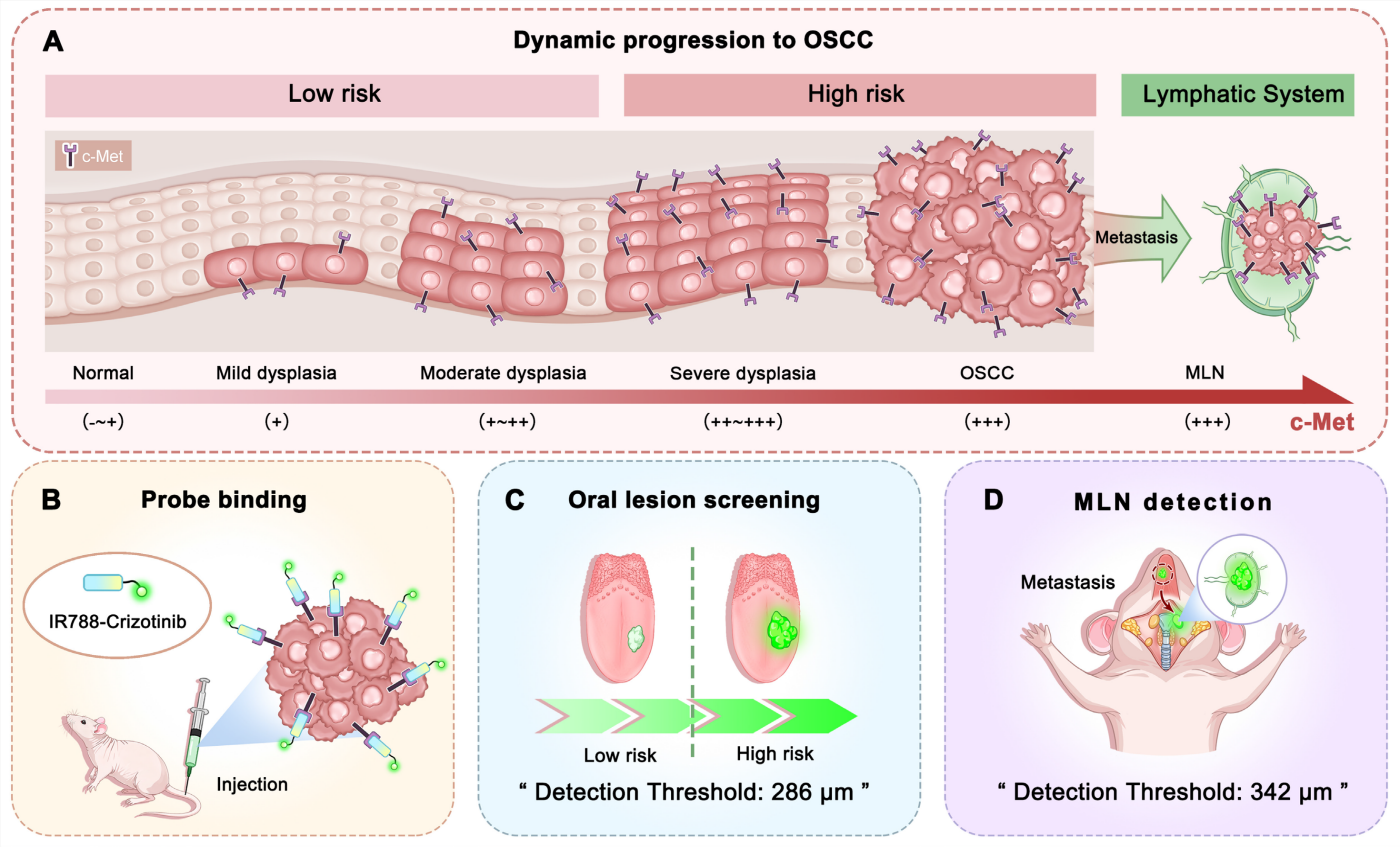
**Schematic overview of c-Met-targeted NIR-II imaging for stepwise management of OSCC. (A)** Progressive increase in c-Met expression from normal oral mucosa to dysplasia and OSCC, with sustained high expression in metastatic tumor deposits within cervical lymph nodes.** (B)** Selective binding of IR788-Crizotinib to c-Met. **(C)** Fluorescence-guided identification of high-risk oral lesions using IR788-Crizotinib, with a minimum detected lesion size of 286 μm.** (D)** Fluorescence-guided identification of metastatic lymph nodes (MLNs) in OSCC using IR788-Crizotinib, with a minimum detected micro-metastatic focus of 342 μm.

On this basis, we developed IR788-Crizotinib, a c-Met-targeted small-molecule fluorescent probe built on the near-infrared fluorophore IR788 and linked to the c-Met inhibitor Crizotinib. We first characterized c-Met expression in human oral lesions, OSCC tissues and metastatic lymph nodes to establish its suitability as an imaging target. We then evaluated the imaging performance of IR788-Crizotinib in subcutaneous xenograft, 4-NQO-induced oral lesion, orthotopic tongue OSCC and cervical lymph node metastasis models. Our findings show that this probe enables identification of high-risk lesions, delineation of primary tumor extent and detection of metastatic lymph nodes (MLN), thereby providing an integrated imaging strategy for OSCC management from premalignant lesion screening to intraoperative navigation (**Figure 1**).

## Materials and Methods

### Ethics statement

The study involving human specimens was approved by the Ethics Committee of Shanghai Ninth People’s Hospital, Shanghai Jiao Tong University School of Medicine (Protocol No. SH9H-2024-T178-2), and written informed consent was obtained from all participants. Animal experiments were approved by the Animal Protection and Use Committee of Shanghai Ninth People’s Hospital, Shanghai Jiao Tong University School of Medicine (Protocol No. SH9H-2023-A832-1) and conducted in accordance with institutional regulations for the care and use of laboratory animals. All experimental mice were obtained from the Experimental Animal Center of Shanghai Ninth People’s Hospital, Shanghai Jiao Tong University, and all procedures complied with the NIH Guide for the Care and Use of Laboratory Animals.

### Clinical samples

Formalin-fixed, paraffin-embedded specimens from oral premalignant lesions, primary OSCC, and metastatic cervical lymph nodes were collected from 59 patients treated at Shanghai Ninth People’s Hospital. Dysplasia diagnosis and grading were independently reviewed by three pathologists according to the World Health Organization criteria 27. For immunohistochemical scoring, staining intensity was classified as 0, 1, 2, or 3, corresponding to negative, weak, moderate, and strong staining, respectively. Semi-quantitative assessment was performed using the H-score method. H-scores were calculated as 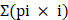, where pi denotes the percentage of positively stained cells (0-100%) at each intensity level and i represents the corresponding intensity score (0-3), yielding a total score ranging from 0 to 300.

### Transcriptomic data analysis

The GSE227919 dataset was obtained from the NCBI Gene Expression Omnibus (GEO). The dataset was generated on the GPL18573 Illumina NextSeq 500 platform and reported as transcripts per million (TPM). A total of 18 healthy control, 21 dysplasia, and 8 OSCC samples were included in the present analysis. TPM values were transformed as log_2_(TPM+1). Statistical analyses were conducted in R with the *rstatix* package (v1.0.0). Differences among groups were evaluated by one-way ANOVA followed by Bonferroni-adjusted pairwise* t* tests.

### Synthesis and characterization

IR788-Crizotinib was synthesized by conjugating IR788 to Crizotinib through a PEG8 linker. Synthetic routes and structural characterization of the probe and related compounds are provided in the Supplementary Material (**Schemes S1-S3, Figure S1-S14**).

### Cell lines and cell culture

Cal27-Luc (human OSCC cells) and HOK (human oral keratinocytes) were obtained from Prof. Youguang Lu (Fujian Medical University) and Dr. Yiyan Pei (West China Hospital of Stomatology), respectively 28, 29. The human OSCC cell line HSC2 was purchased from Anwei-sci Cell Center (Shanghai, China). All lines were confirmed to be mycoplasma-free before use. Cal27-Luc and HSC2 cells were maintained in high-glucose DMEM (Gibco) supplemented with 10% fetal bovine serum (FBS) and 1% penicillin-streptomycin. HOK cells were cultured in Defined Keratinocyte-SFM (Gibco, 10744-019) supplemented with 1% penicillin-streptomycin. Cultures were maintained at 37 °C in 5% CO_2_.

### Confocal validation of c-Met targeting

For cell-based validation, CAL27-Luc, HSC2, and HOK cells were seeded into 35-mm glass-bottom dishes at 3 × 10^5^ cells/mL (1 mL per dish) and cultured for 24 h. Cells were then incubated with BODIPY-Crizotinib/IR788-Crizotinib (1 μM) for 2 h at 37 °C in the dark. For the blocking group, CAL27-Luc cells were pre-incubated with Crizotinib (10 μM) for 1 h before probe treatment. To control for the effect of the solvent, all other groups received the same final concentration of DMSO as the blocking group. After washing with PBS, cells were fixed in 4% paraformaldehyde, permeabilized with 0.1% Triton X-100, and blocked with 5% BSA. Cells were incubated overnight at 4 °C with anti-MET rabbit monoclonal antibody (Abcam, ab51067, 1:200), followed by Cy5-conjugated secondary antibody (Abcam, ab6564, 1:500) for 1 h at room temperature in the dark. Nuclei were stained with DAPI for 10 min. Images were acquired on an Olympus FV4000 confocal microscope (Olympus, Japan). Because the available confocal system provided excitation up to 730 nm, which did not match the optimal excitation condition of IR788-Crizotinib, BODIPY-Crizotinib was initially used as a surrogate probe for conventional confocal assessment. Supplementary imaging with IR788-Crizotinib was additionally performed to compare probe localization with c-Met immunofluorescence.

For tissue-level analysis, paraffin sections from mouse tongue tumors were processed by deparaffinization, rehydration, and citrate-based antigen retrieval. Endogenous peroxidase activity was quenched, followed by blocking with 3% BSA. Slides were incubated overnight at 4 °C with anti-MET rabbit monoclonal antibody (Abcam, ab51067, 1:500), followed by HRP-conjugated goat anti-rabbit IgG (Abcam, ab205718, 1:5000) for 50 min at room temperature and TSA 670 working solution (HISTOV, DFT67100) for 10 min in the dark. After PBS washing, sections were incubated with IR788-Crizotinib at 37 °C for 4 h, counterstained with DAPI, and mounted with antifade medium. Images were collected on an Olympus FV4000 microscope. Colocalization between probe fluorescence and c-Met immunofluorescence in cells and tumor tissues was quantified in Image*J* using Pearson’s correlation coefficient.

### Mouse models of OSCC and lymph node metastasis

Subcutaneous and orthotopic xenografts were established in BALB/c nude mice. For subcutaneous xenografts, 2 × 10^6^ Cal27-Luc or HSC2 cells were injected into the right flank. For orthotopic tongue xenografts, 2 × 10^5^ Cal27-Luc cells were injected into the anterior tongue.

For chemically induced oral carcinogenesis, male SPF C57BL/6 mice received 4-nitroquinoline 1-oxide (4-NQO, 100 μg/mL, Sigma-Aldrich, N8141) in sterile drinking water for 16 weeks, followed by regular sterile water for 2 additional weeks before lesion assessment 26, 30. This model recapitulates multistep oral carcinogenesis, progressing from normal mucosa through dysplasia to OSCC.

For the cervical lymph node metastasis model, BALB/c nude mice received orthotopic inoculation of 2 × 10^5^ Cal27-Luc cells into the anterior tongue. Cervical lymph node metastasis was monitored by bioluminescence imaging using an IVIS Spectrum system (PerkinElmer). Metastatic cervical lymph nodes were identified by bioluminescent signals.

### *In vivo* and *ex vivo* NIR-II imaging

Mice underwent isoflurane anesthesia in medical air before image acquisition (4% for induction and 1%-2% for maintenance). IR788-Crizotinib was administered through the tail vein at 1.5 μg/g body weight. NIR-II imaging of whole animals and excised tissues was performed on a MARS-FAST system (Artemis Intelligent Imaging, Shanghai, China). The imaging parameters were kept constant across experiments, including 808 nm excitation, 75.5 mW/cm^2^ laser power density, 50 ms exposure, and a 1000 nm long-pass filter. No additional gain adjustment was applied during comparative image acquisition. Quantitative image analysis was conducted in Image*J*.

### Statistical analysis

All statistical analyses were performed using GraphPad Prism (v9.5.0), and data are presented as mean ± SD. Comparisons between two groups were conducted using two-tailed Student’s *t*-test or paired *t*-test, as appropriate. Comparisons among multiple groups were performed using one-way analysis of variance (ANOVA) followed by Tukey’s multiple-comparison test. A two-sided *p* < 0.05 was considered statistically significant. Statistical significance was denoted as follows: * *p* < 0.05, ** *p* < 0.01, *** *p* < 0.001, and **** *p* < 0.0001.

## Results

### c-Met upregulation across oral lesion progression, primary tumors, and metastatic lymph nodes

**Figure 2:**
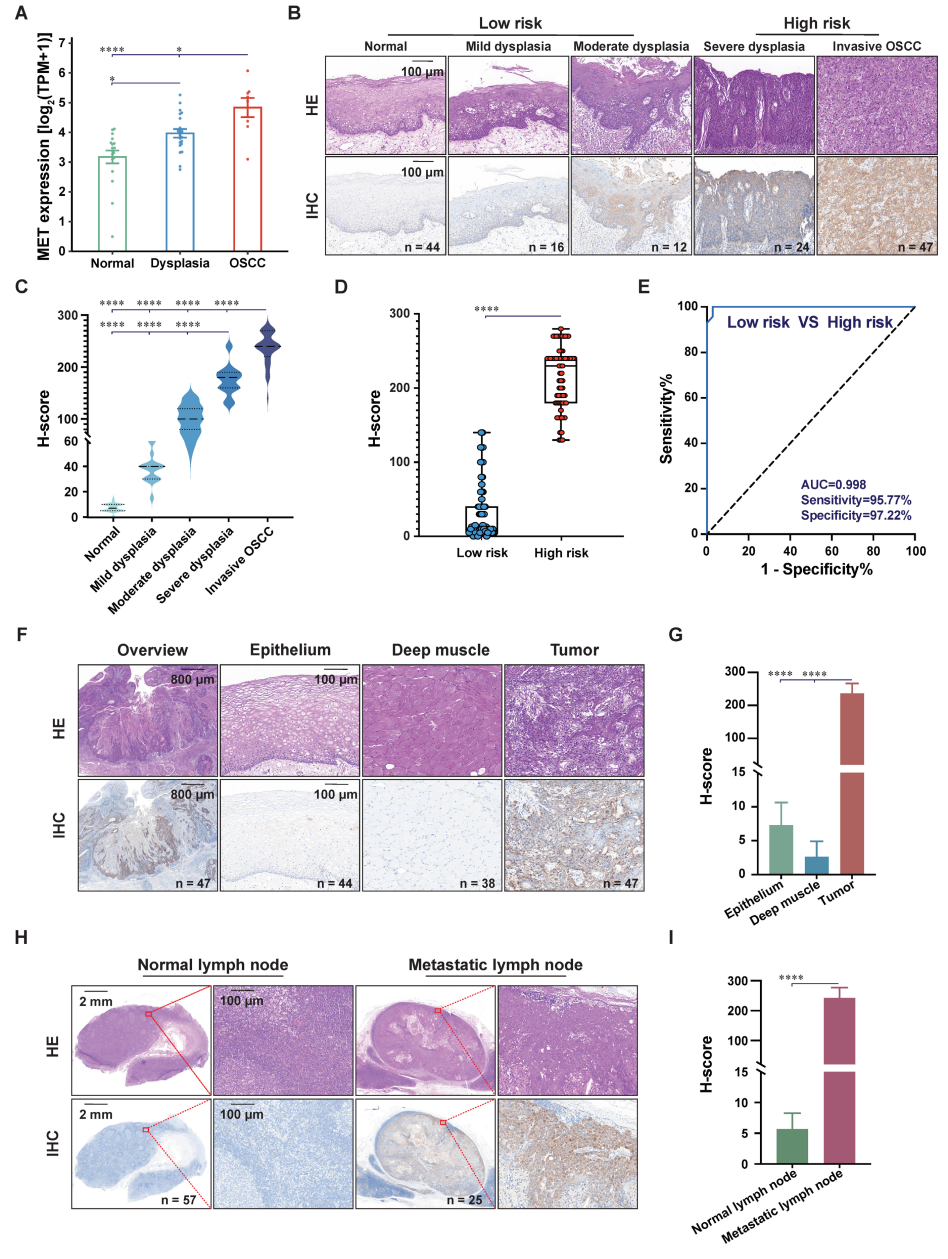
**c-Met expression across the progression from normal epithelium to invasive OSCC and lymph node metastasis in human specimens. (A)** Stepwise increase in MET expression from normal oral mucosa through dysplasia to OSCC in a GEO dataset.** (B)** Representative IHC and H&E images showing c-Met expression across the progression of OSCC, including normal oral epithelium (n = 44), mild dysplasia (n = 16), moderate dysplasia (n = 12), severe dysplasia (n = 24), and OSCC (n = 47). Normal to moderate dysplasia were classified as low-risk lesions, whereas severe dysplasia and invasive OSCC were classified as high-risk lesions. Scale bar, 100 µm.** (C)** Quantitative analysis of c-Met expression in epithelial or tumor regions of IHC specimens using the H-score method (**** *p* < 0.0001; one-way ANOVA followed by Tukey’s HSD test).** (D)** Comparison of H-scores between low-risk and high-risk groups (**** p < 0.0001; two-tailed Student’s t-test).** (E)** ROC analysis of H-scores for distinguishing low-risk from high-risk lesions.** (F)** Representative IHC images of c-Met expression in epithelial, deep muscle, and tumor regions of OSCC resection specimens, with corresponding H&E staining. Scale bars, 800 µm and 100 µm.** (G)** Quantitative comparison of c-Met expression between paired tumor and adjacent non-tumorous tissues from 47 patients (**** p < 0.0001; paired two-tailed *t*-test).** (H)** Representative IHC images of normal and metastatic lymph nodes with corresponding H&E staining. Scale bars, 2 mm and 100 µm.** (I)** Quantitative analysis of c-Met expression by H-score in paired normal and metastatic lymph nodes (**** p < 0.0001; paired two-tailed *t*-test).

MET mRNA was progressively upregulated across oral mucosal lesions in the GSE227919 dataset. Compared with normal mucosa (3.17 ± 0.91), expression was higher in dysplasia (3.97 ± 0.65, * *p* < 0.05) and further increased in OSCC (4.83 ± 0.91, **** *p* < 0.0001; **Figure 2A**). This pattern was reproduced in resected human specimens. Immunohistochemistry showed absent-to-weak c-Met staining in normal oral epithelium, with physiological expression largely restricted to the basal layer, whereas staining increased in mild and moderate dysplasia and became prominent in severe dysplasia and OSCC (**Figure 2B**). H-score analysis confirmed a graded increase with lesion severity, reaching the highest level in OSCC (normal mucosa: 7.27 ± 3.36; mild dysplasia: 38.44 ± 11.51; moderate dysplasia: 102.50 ± 26.33; severe dysplasia: 174.60 ± 28.74; OSCC: 236.40 ± 30.03; **** *p* < 0.0001; **Figure 2C**).

When lesions were stratified into low-risk (normal to moderate dysplasia) and high-risk (severe dysplasia and OSCC) groups, c-Met expression differed markedly between the two categories (30.07 ± 36.97 vs. 215.50 ± 40.60, **** *p* < 0.0001; **Figure 2D**). ROC analysis showed that c-Met H-score distinguished high-risk from low-risk lesions with high accuracy, yielding an AUC of 0.998, a sensitivity of 95.77%, and a specificity of 97.22% (**Figure 2E**).

In OSCC resection specimens, c-Met expression was markedly higher in tumor regions than in adjacent non-neoplastic mucosa or deep muscle, both of which showed minimal to undetectable staining (**Figure 2F**). H-score analysis confirmed significantly higher expression in tumor regions than in adjacent mucosa and deep muscle (tumor: 236.40 ± 30.03; adjacent mucosa: 7.27 ± 3.35; deep muscle: 2.63 ± 2.28; **** *p* < 0.0001; **Figure 2G**). A similar pattern was observed in cervical lymph nodes: metastatic tumor deposits showed strong c-Met immunoreactivity, whereas normal lymph nodes showed minimal staining, with markedly different H-scores between metastatic and normal nodes (242.00 ± 34.52 vs. 5.67 ± 2.59, **** *p* < 0.0001; **Figure 2H-I**). Together, these data identify c-Met as a biologically relevant target across lesion progression, primary tumor margins, and metastatic lymph node deposits, thereby providing the rationale for probe development.

### Construction and optical properties of the c-Met-targeted probe IR788-Crizotinib

**Figure 3:**
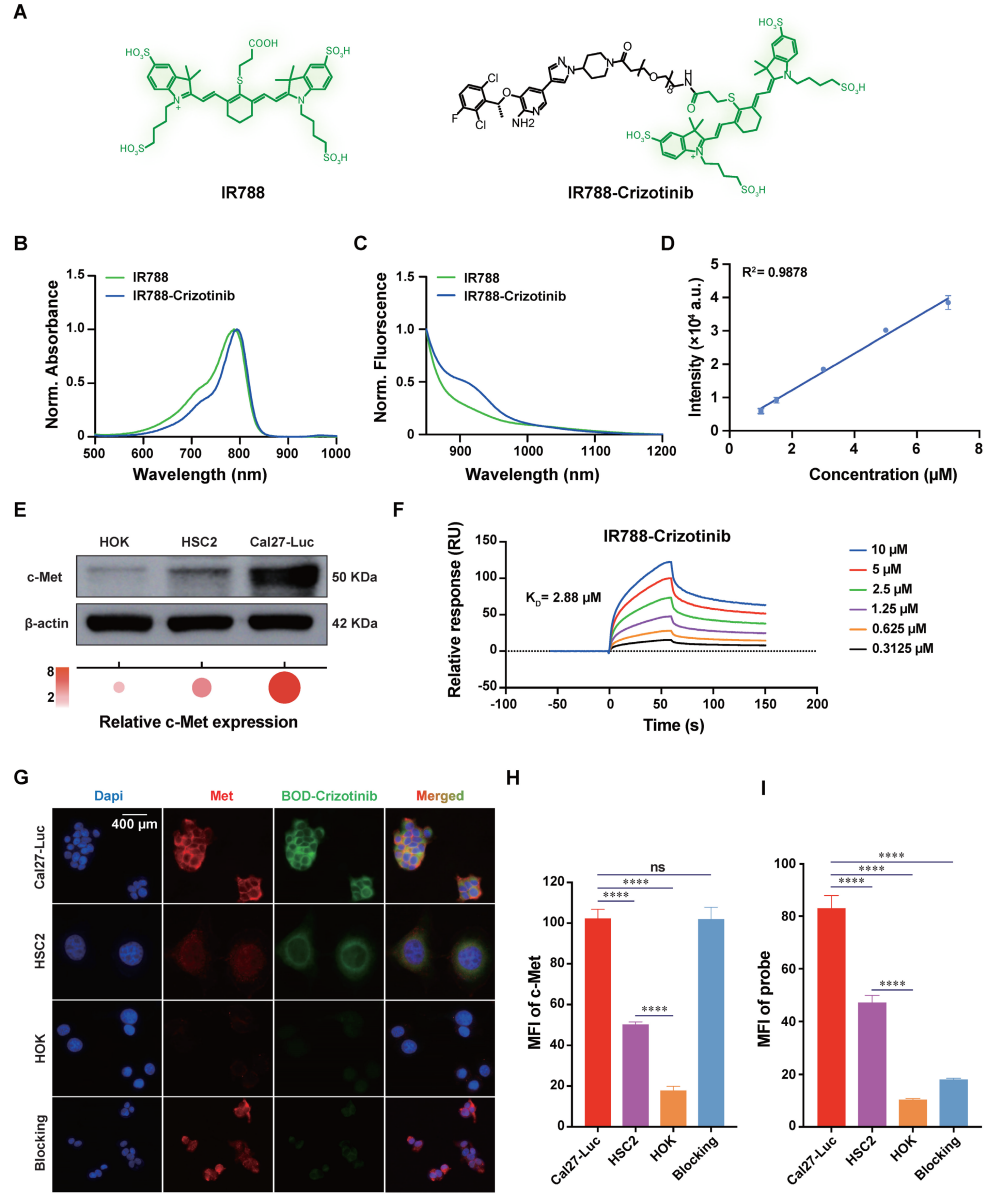
***In vitro* characterization of IR788-Crizotinib and evaluation of its binding to c-Met. (A)** Chemical structures of IR788 and IR788-Crizotinib. **(B)** Absorption spectra of IR788 and IR788-Crizotinib. **(C)** Normalized NIR-II fluorescence emission spectra. **(D)** Fluorescence intensity curves of IR788-Crizotinib at different concentrations. **(E)** Western blot analysis of c-Met expression in different cell lines. **(F)** Surface plasmon resonance (SPR) analysis of the binding of IR788-Crizotinib to c-Met. **(G)** Representative confocal images of Cal27-Luc, HSC2, HOK, and Crizotinib-blocked Cal27-Luc cells after incubation with BODIPY-Crizotinib (BOD-Crizotinib) for 2 h, showing DAPI (blue), c-Met immunofluorescence (red), and BODIPY-Crizotinib fluorescence (green). Scale bar, 400 μm. **(H, I)** Quantitative analysis of cellular c-Met expression and BODIPY-Crizotinib uptake (**** p < 0.0001; ns, not significant; one-way ANOVA followed by Tukey’s HSD test).

On the basis of this target profile, we constructed the c-Met-targeted fluorescent probe IR788-Crizotinib by conjugating the near-infrared fluorophore IR788 to the c-Met-targeting small molecule Crizotinib through a PEG8 linker. The resulting probe was obtained with high analytical purity, as confirmed by HPLC (**Figure 3A** and **Figure S15**). Spectral characterization showed that IR788 had a maximum absorption peak at 788 nm, whereas IR788-Crizotinib exhibited a red-shifted absorption maximum at 794 nm (**Figure 3B**). IR788-Crizotinib also showed an emission tail extending into the 900-1,200 nm range, consistent with NIR-II imaging (**Figure 3C**). Its molar extinction coefficient was 9.992 × 10^4^ M^-1^cm^-1^ (**Figure S16**), and probe concentration correlated linearly with NIR-II fluorescence intensity over the tested range (R^2^ = 0.9878; **Figure 3D**).

For comparison, we synthesized the corresponding ICG-labeled analogue, ICG-Crizotinib (**Figure S17**), and compared IR788-Crizotinib, IR788, ICG-Crizotinib, and ICG in terms of quantum yield, photostability, and tissue penetration. Using IR-26 as the reference, the quantum yields of IR788-Crizotinib, IR788, ICG, and ICG-Crizotinib were 0.22%, 0.17%, 0.31%, and 0.06%, respectively (**Figure S18**). For all probes, absorbance at 808 nm correlated linearly with integrated fluorescence intensity, supporting the reliability of the measurements. Crizotinib conjugation had only a limited effect on the fluorescence efficiency of the IR788 scaffold, whereas a more pronounced loss was observed for the ICG-derived probe.

Under continuous 808 nm laser irradiation, fluorescence from all four probes declined over time, but the decay profiles differed substantially (**Figure S19**). IR788-Crizotinib showed the slowest signal loss and maintained the highest normalized fluorescence throughout the irradiation period. After 30 min of continuous exposure, its normalized fluorescence intensity remained 0.51 ± 0.03, significantly higher than that of IR788 (0.22 ± 0.01), ICG (0.21 ± 0.04), and ICG-Crizotinib (0.39 ± 0.05) (* *p* < 0.05, **** *p* < 0.0001; **Figure S19B**). IR788-Crizotinib also remained optically stable across a physiologically relevant pH range. After incubation at pH 6.5, 7.4, and 8.0 for 0.5 h, 2 h, and 20 h, fluorescence intensity varied only minimally, and absorption spectra remained largely superimposable without appreciable peak shifts or spectral distortion (**Figure S20**).

Tissue penetration was then evaluated *in vitro* and* in vivo* (**Figure S21**). In the capillary-based phantom assay using 1% intralipid as the scattering medium, fluorescence from all probes weakened with increasing depth; however, at 5 mm, the SBR of IR788-Crizotinib remained 4.48 ± 0.30, significantly higher than that of IR788 (1.39 ± 0.05), ICG (1.23 ± 0.03), and ICG-Crizotinib (1.01 ± 0.05) (**** *p* < 0.0001; **Figure S21C**). Even at 6 mm, its SBR remained 2.52 ± 0.18. This advantage was maintained* in vivo*, where IR788-Crizotinib remained detectable at a tissue depth of 4.97 mm with an SBR of 3.96 ± 0.61. Collectively, these properties indicate that IR788-Crizotinib retains a favorable optical profile after c-Met ligand conjugation and is suitable for subsequent biological evaluation.

### *In vitro* validation of c-Met-specific binding by IR788-Crizotinib

We next examined whether IR788-Crizotinib retained specific binding to c-Met *in vitro*. To define suitable cell models for subsequent NIR imaging, we first examined c-Met expression in two human OSCC cell lines (Cal27-Luc and HSC2) and in normal human oral keratinocytes (HOK). Western blot analysis showed markedly higher c-Met protein levels in Cal27-Luc and HSC2 cells than in HOK cells, with the highest expression in Cal27-Luc cells (**Figure 3E**). These two OSCC lines were therefore selected to represent high and intermediate c-Met expression in subsequent xenograft studies.

SPR confirmed direct interaction between IR788-Crizotinib and c-Met, with a dissociation constant (K_D_) of 2.88 μM (**Figure 3F**). To examine cellular targeting, we first performed confocal imaging using BODIPY-Crizotinib (**Figure 3G**). BODIPY-Crizotinib fluorescence showed strong colocalization with c-Met immunofluorescence, with a Pearson’s correlation coefficient of 0.915 (**Figure S22**). Signal intensity followed the expected expression gradient, being highest in Cal27-Luc cells, intermediate in HSC2 cells, and minimal in HOK cells. Competitive blocking with excess unlabeled Crizotinib markedly reduced fluorescence in Cal27-Luc cells, consistent with receptor-dependent binding. Quantitative analysis similarly showed the highest c-Met expression (**** *p* < 0.0001,** Figure 3H**) and probe uptake (**** *p* < 0.0001, **Figure 3I**) in Cal27-Luc cells, whereas HSC2 cells also showed significantly higher fluorescence than HOK controls (**** *p* < 0.0001).

As an additional validation step, we performed confocal imaging using IR788-Crizotinib. Although fluorescence intensity under the available confocal settings was lower than that of BODIPY-Crizotinib because excitation conditions did not fully match the optimal excitation wavelength of IR788-Crizotinib, the signal distribution remained consistent with c-Met expression: fluorescence was strongest in Cal27-Luc cells, intermediate in HSC2 cells, and weak in HOK cells, and was reduced in the blocking group (**Figure S23A**). Colocalization analysis between IR788-Crizotinib fluorescence and anti-c-Met staining yielded a Pearson’s correlation coefficient of 0.901 (**Figure S23B**). These results show that IR788-Crizotinib retains a cellular targeting pattern consistent with c-Met expression, providing direct support for its subsequent evaluation *in vivo*.

### *In vivo* c-Met-dependent targeting of IR788-Crizotinib in OSCC models

**Figure 4:**
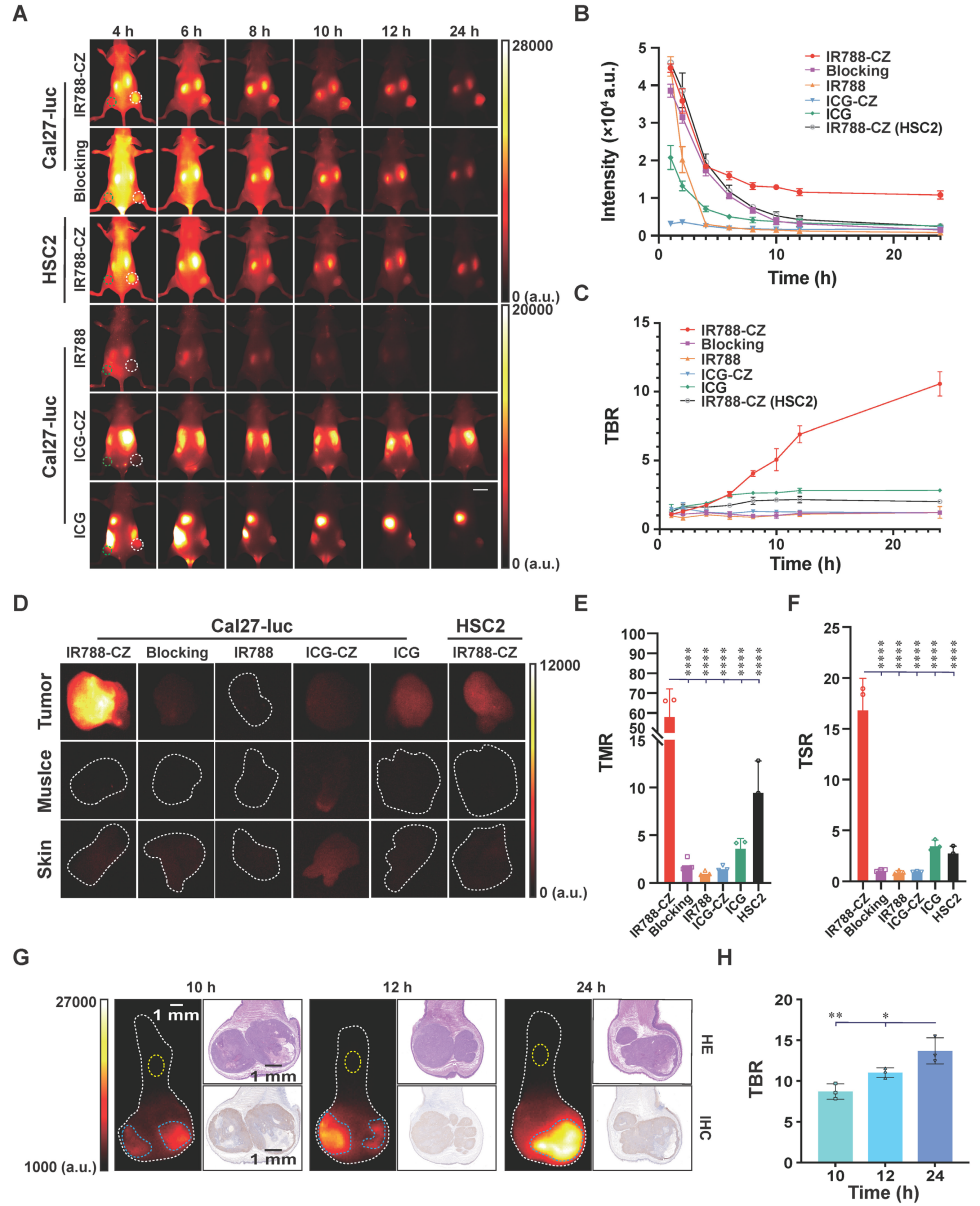
**NIR-II imaging of IR788-Crizotinib in subcutaneous and orthotopic mouse models. (A)** Representative whole-body NIR-II images of Cal27-Luc and HSC2 subcutaneous xenografts acquired at 4, 6, 8, 10, 12, and 24 h after intravenous injection of IR788-Crizotinib. In the Cal27-Luc subcutaneous xenograft model, additional comparator groups included a Crizotinib blocking group, as well as ICG, ICG-Crizotinib, and IR788 (n = 3). The white dashed outlines indicate the tumor regions, and the green dashed outlines indicate the background regions. Scale bar, 1 cm.** (B, C)** Quantitative analysis of tumor fluorescence intensity and tumor-to-background ratio (TBR) over time for each group.** (D)** *Ex vivo* NIR-II fluorescence images of tumor, muscle, and skin collected at 24 h from the IR788-Crizotinib, Crizotinib blocking, IR788, ICG, ICG-Crizotinib, and HSC2 xenograft groups. **(E, F)** Quantitative comparison of tumor-to-muscle ratio (TMR) and tumor-to-skin ratio (TSR) calculated from* ex vivo* NIR-II fluorescence signals (**** *p* < 0.0001; one-way ANOVA followed by Tukey’s HSD test). **(G)** *Ex vivo* NIR-II images of Cal27-Luc orthotopic tongue xenografts at 10, 12, and 24 h post-injection, with corresponding H&E and c-Met IHC staining. The blue dashed outline indicates the tumor ROI, and the yellow dashed outline indicates the background ROI. Scale bar, 1 mm. **(H)** Quantitative analysis of TBR in orthotopic lesions at the indicated time points (* p < 0.05, ** *p* < 0.01; one-way ANOVA followed by Tukey’s HSD test). Imaging parameters: Laser at 808 nm, power at 75.5 mW·cm^-2^, exposure time of 50 ms, with a 1000 nm long-pass filter. CZ, Crizotinib.

We therefore evaluated the *in vivo* targeting performance of IR788-Crizotinib in OSCC models. Subcutaneous xenografts were established using Cal27-Luc and HSC2 cells, representing high and intermediate c-Met expression, respectively. In the Cal27-Luc xenograft model, tumor-associated fluorescence was readily identifiable from 4 h after intravenous injection. Although the absolute tumor signal gradually declined thereafter, background fluorescence decreased more rapidly, resulting in a progressive increase in tumor-to-background ratio (TBR), from 1.76 ± 0.10 at 4 h to 2.56 ± 0.16 at 6 h, 4.06 ± 0.21 at 8 h, 5.06 ± 0.81 at 10 h, 6.19 ± 0.62 at 12 h, and 10.57 ± 0.88 at 24 h (**Figure 4A-C**). By contrast, the blocking, ICG, ICG-Crizotinib, IR788, and HSC2 groups all showed lower contrast at 24 h, with TBR values of 1.20 ± 0.07, 2.83 ± 0.13, 1.21 ± 0.09, 1.22 ± 0.43, and 2.01 ± 0.02, respectively (**** *p* < 0.0001, **Figure S24**). IHC was consistent with these imaging results, confirming higher c-Met expression in Cal27-Luc tumors than in HSC2 tumors (**Figure S25**).

Accordingly, *ex vivo* NIR-II fluorescence imaging was performed at 24 h post-administration. Relative to the blocking, IR788, ICG-Crizotinib, ICG, and HSC2 xenograft groups, the IR788-Crizotinib-treated Cal27-Luc group showed markedly higher tumor-to-muscle ratios (TMR = 58.44 ± 13.68) and tumor-to-skin ratios (TSR = 16.90 ± 3.06). The corresponding TMR and TSR values were 1.96 ± 0.69 and 1.11 ± 0.11 in the blocking group, 1.05 ± 0.24 and 0.92 ± 0.17 in the IR788 group, 1.54 ± 0.27 and 0.93 ± 0.03 in the ICG-Crizotinib group, 3.65 ± 1.00 and 3.53 ± 0.52 in the ICG group, and 9.52 ± 3.27 and 2.81 ± 0.64 in the HSC2 group (**** *p* < 0.0001, **Figure 4D-F**). These data indicate preferential accumulation of IR788-Crizotinib in c-Met-high tumors, with improved *ex vivo* imaging contrast.

The probe performance was then assessed in an orthotopic tongue OSCC model established with Cal27-Luc cells. After intravenous administration of IR788-Crizotinib, tongues were harvested at 10, 12, and 24 h for *ex vivo* imaging (**Figure 4G**; workflow in **Figure S26**). Fluorescence remained localized to the tumor-bearing region, and TBR increased from 8.70 ± 0.93 at 10 h to 11.02 ± 0.61 at 12 h and 13.69 ± 1.60 at 24 h (* *p* < 0.05, ** *p* < 0.01,** Figure 4H**). H&E staining and c-Met immunohistochemistry further showed spatial correspondence between areas of high NIR-II signal and histologically confirmed tumor tissue (**Figure 4G**). Supplementary confocal imaging further showed that IR788-Crizotinib fluorescence overlapped with c-Met immunofluorescence in orthotopic tongue tumor sections and that the probe-positive area corresponded to the histologically defined tumor boundary, supporting its potential for tumor delineation (**Figure S27**). Together, these findings indicate that IR788-Crizotinib supports c-Met-dependent imaging of OSCC *in vivo* and establishes the basis for its subsequent evaluation in spontaneous oral lesion, nodal metastasis, and fluorescence-guided surgery models.

### Biodistribution and biosafety evaluation of IR788-Crizotinib

To further define the* in vivo* behavior of IR788-Crizotinib, we examined biodistribution and biosafety.* Ex vivo* fluorescence imaging showed that IR788-Crizotinib and IR788 produced their strongest signals in the kidneys, followed by the liver, consistent with predominant renal clearance and secondary hepatic uptake. Tumor-associated fluorescence was more evident in the IR788-Crizotinib group than in the other groups. In contrast, ICG and ICG-Crizotinib showed the highest fluorescence in the liver, consistent with hepatobiliary disposition (**Figure S28-S29**).

We next assessed *in vitro* and *in vivo* biosafety. Cal27-Luc and HOK cells were incubated with IR788-Crizotinib at concentrations ranging from 0.625 to 10 μM, and viability was measured using a Cell Counting Kit-8 assay. No appreciable cytotoxicity was observed within this concentration range (**Figure S30A**). For* in vivo* assessment, mice received a single tail-vein injection of IR788-Crizotinib at 15 μg/g. Serum biochemical analyses performed on days 14 and 28 after injection showed no significant differences in liver or kidney function parameters compared with PBS-treated controls (ns, *p* > 0.05, **Figure S30B-C**). In addition, H&E staining of major organs revealed no evident probe-related tissue injury (**Figure S30D**). Collectively, the data support that IR788-Crizotinib is cleared predominantly through the kidneys and remains well tolerated under the conditions tested, supporting its further evaluation for *in vivo* imaging applications.

### IR788-Crizotinib-based NIR-II imaging enables identification of high-risk oral lesions

**Figure 5:**
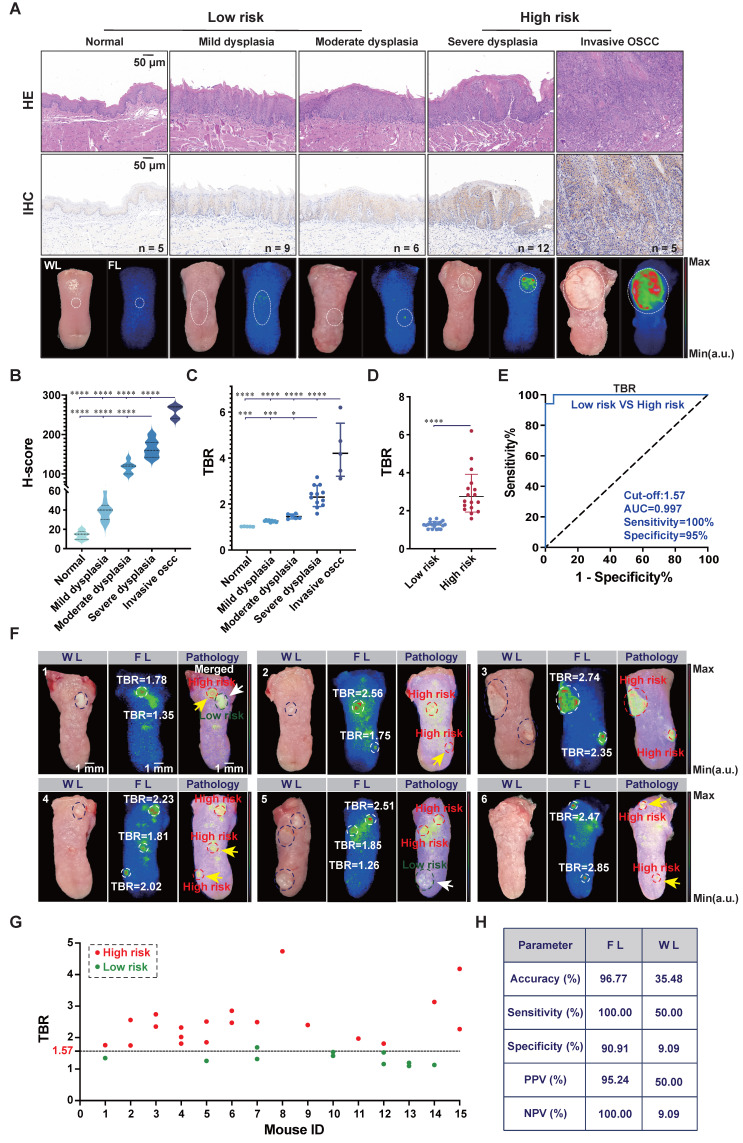
** IR788-Crizotinib-based NIR-II imaging for monitoring c-Met-associated progression of 4-NQO-induced tongue lesions in mice. (A)** Representative tongue lesions at different pathological stages, showing c-Met IHC, H&E, and the corresponding NIR-II fluorescence and white-light images of the biopsy regions. The white dashed circles indicate the biopsy regions. Normal mucosa to moderate dysplasia were classified as low-risk lesions, whereas severe dysplasia and invasive OSCC were classified as high-risk lesions. Scale bars, 50 μm and 1 mm.** (B)** Quantitative analysis of c-Met H-scores in mouse tongue tissues of different pathological types (n = 37), assessed in epithelial or tumor regions (**** *p* < 0.0001; one-way ANOVA followed by Tukey’s HSD test). **(C)** Quantitative analysis of tumor-to-background ratio (TBR) from IR788-Crizotinib NIR-II imaging in lesions of different pathological types (* *p* < 0.05, *** *p* < 0.001, **** *p* < 0.0001; one-way ANOVA followed by Tukey’s HSD test). **(D)** Comparison of TBR between low-risk and high-risk groups (**** *p* < 0.0001; unpaired two-tailed Student’s *t*-test).** (E)** ROC analysis of TBR for distinguishing low-risk from high-risk lesions. **(F)** Representative comparison of biopsy guidance by white light (WL) and NIR-II fluorescence (FL) at 24 h after probe injection, including white-light, fluorescence, and fusion images. The blue dashed lines indicate suspicious high-risk regions identified by white light, whereas the white dashed lines indicate fluorescence-identified regions. The TBR values of all selected regions are shown on the fluorescence image, and the corresponding pathological results are shown on the fusion image. The red dashed lines delineate high-risk lesions, and the green dashed lines delineate low-risk lesions. White arrows indicate false-positive regions identified by white light, and yellow arrows indicate high-risk lesions missed by white light. Scale bar, 1 mm. **(G)** Summary of TBR values and pathological grades for all biopsied regions (n = 31). **(H)** Comparison of the diagnostic performance of FL and WL, including accuracy, sensitivity, specificity, positive predictive value (PPV), and negative predictive value (NPV). Imaging parameters: Laser at 808 nm, power at 75.5 mW·cm^-2^, exposure time of 50 ms, with a 1000 nm long-pass filter.

We next examined whether IR788-Crizotinib-based NIR-II imaging could distinguish oral lesions at different stages of disease progression in the 4-NQO-induced spontaneous oral carcinogenesis model. On the basis of prior optimization in the subcutaneous and orthotopic xenograft models, all imaging in this model was performed at 24 h after injection, a time point that provided favorable contrast (workflow in **Figure S31**). A total of 22 4-NQO-treated mice and 5 normal controls were included, yielding 37 fluorescence-guided tissue samples. c-Met expression increased progressively with lesion severity, with H-scores of 13.80 ± 4.44 in normal mucosa, 39.44 ± 10.47 in mild dysplasia, 116.70 ± 15.06 in moderate dysplasia, 165.80 ± 21.93 in severe dysplasia, and 258.00 ± 16.43 in OSCC (**** *p* < 0.0001, **Figure 5A-B**). This pattern was consistent with that observed in human oral lesions and further supported the relevance of c-Met to oral cancer progression.

Consistent with increasing c-Met expression, TBR values obtained by NIR-II imaging rose with lesion severity (**Figure 5C**). Severe dysplasia and OSCC showed TBR values of 2.34 ± 0.46 and 4.34 ± 1.22, respectively, both significantly higher than those of normal mucosa (1.02 ± 0.01), mild dysplasia (1.26 ± 0.04), and moderate dysplasia (1.46 ± 0.10) (* *p* < 0.05, *** *p* < 0.001, **** *p* < 0.0001, **Figure 5C**). Correlation analysis further showed a strong positive association between c-Met expression and TBR (correlation coefficient = 0.8592, **Figure S32**). When lesions were stratified by risk, the high-risk group, defined as severe dysplasia and OSCC, showed a markedly higher TBR than the low-risk group, defined as normal mucosa to moderate dysplasia (2.93 ± 1.18 vs. 1.26 ± 0.18, **** *p* < 0.0001; **Figure 5D**). ROC analysis identified 1.57 as the optimal TBR cut-off for distinguishing high-risk from low-risk lesions, corresponding to the maximum Youden index and yielding a sensitivity of 100%, a specificity of 95%, and an AUC of 0.997 (**Figure 5E**).

To assess the practical value of this approach for identifying high-risk oral lesions, we compared fluorescence-guided biopsy selection with conventional white-light guidance (workflow in **Figure S33**). Under NIR-II fluorescence and white-light guidance, 31 suspicious lesions were biopsied from the tongues of 15 mice. Histopathological analysis confirmed 20 high-risk lesions, including 17 severe dysplasia and 3 OSCC, and 11 low-risk lesions, including 6 mild dysplasia and 5 moderate dysplasia. Histopathological examination of the remaining tongue tissue after biopsy revealed no residual high-risk lesions. Representative examples of fluorescence-guided and white-light-guided biopsies are shown in** Figure 5F**, with corresponding pathological images in **Figure S34-S35**.

Fluorescence-guided biopsy identified 21 suspicious lesions, of which 20 were pathologically confirmed as high-risk and 1 as low-risk. By contrast, white-light-guided biopsy identified 20 suspicious lesions, but only 10 were high-risk, whereas the remaining 10 were low-risk (**Figure 5G**). IR788-Crizotinib-based NIR-II imaging detected all high-risk lesions and yielded only one misclassification in the low-risk group, corresponding to a moderate dysplasia (**Figure S36**). White-light guidance showed limited performance in identifying smaller high-risk lesions, with an overall sensitivity of 50%, a specificity of 9.09%, and an accuracy of 35.48% (**Figure 5H**). By comparison, IR788-Crizotinib-based NIR-II imaging achieved a sensitivity of 100%, a specificity of 90.91%, and an accuracy of 96.77% (**Figure 5H**). Notably, this approach detected severe dysplastic lesions as small as 300 μm and microinvasive OSCC lesions as small as 286 μm (**Figure S35**). These findings indicate that IR788-Crizotinib-based NIR-II imaging enables non-invasive identification of high-risk oral lesions and supports real-time guidance for biopsy selection.

### IR788-Crizotinib-based NIR-II imaging enables identification of cervical MLN in OSCC

**Figure 6:**
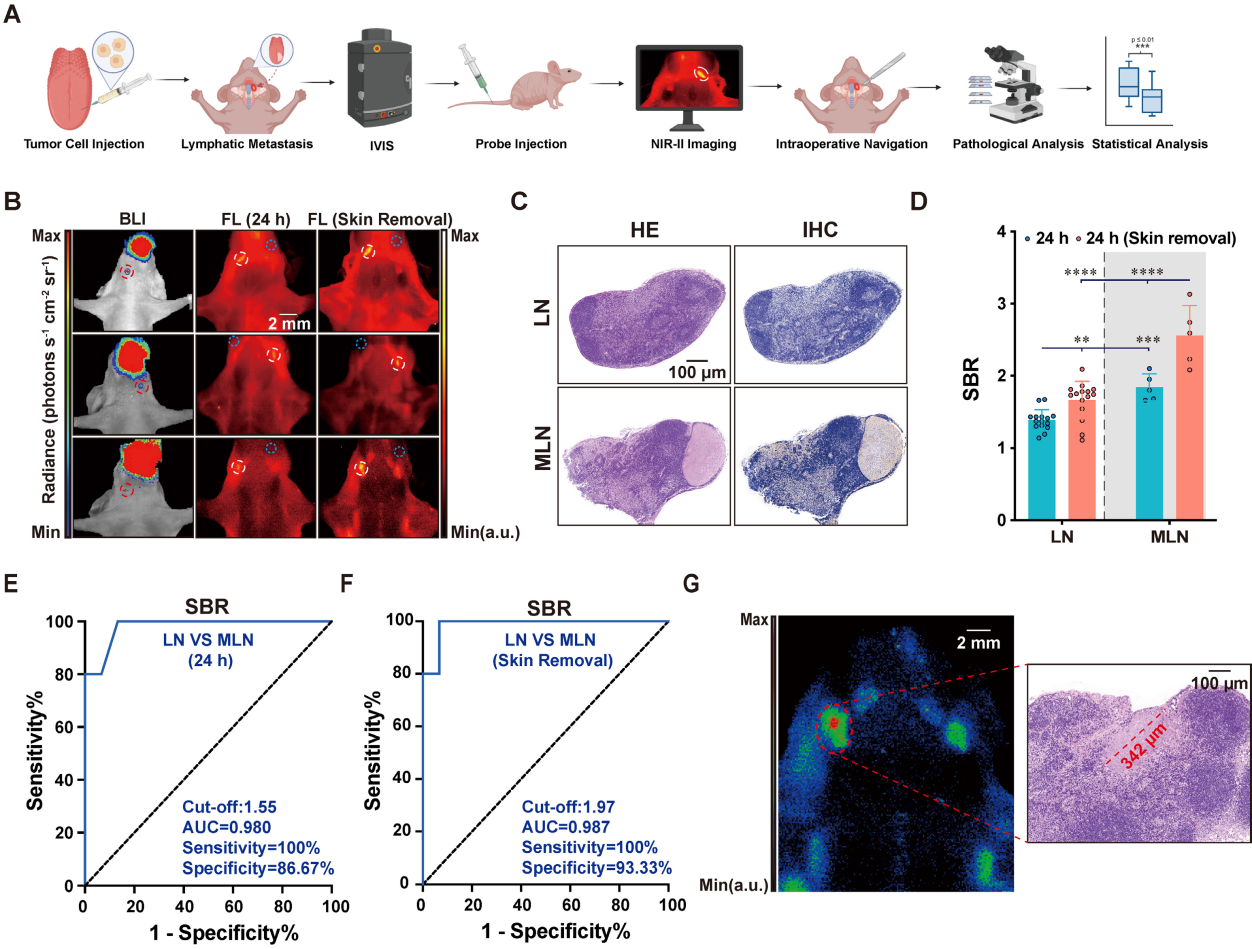
** IR788-Crizotinib-based NIR-II imaging for detection of metastatic cervical lymph nodes. (A)** Schematic workflow for establishing a murine oral squamous cell carcinoma lymph node metastasis model and performing NIR-II fluorescence-guided detection of metastatic lymph nodes. Created in BioRender. Ma, Q. (2026) https://BioRender.com/0l5qyhi. **(B)** Representative bioluminescence imaging (BLI) and NIR-II fluorescence (FL) images showing cervical lymph node metastasis at 24 h after probe injection and after skin removal. The red dashed outlines and white dashed outlines indicate metastatic lymph nodes, and the blue dashed outlines indicate the background masseter muscle region. Scale bar, 2 mm. **(C)** Representative H&E and c-Met IHC images of normal lymph nodes and metastatic lymph nodes (MLN). Metastatic foci within lymph nodes showed strong c-Met positivity, whereas normal lymph nodes (LN) showed minimal to no staining. Scale bar, 100 μm. **(D)** Quantitative comparison of signal-to-background ratio (SBR) between normal and metastatic lymph nodes at 24 h post-injection and after skin removal (** *p* < 0.01, *** *p* < 0.001, **** *p* < 0.0001; paired two-tailed *t*-test). **(E, F)** ROC analysis of SBR for discriminating normal and metastatic lymph nodes at 24 h post-injection and after skin removal.** (G)** Representative NIR-II fluorescence image of the smallest metastatic focus detected, with corresponding H&E staining. The smallest metastatic focus detected by NIR-II imaging measured 342 μm. Scale bars, 2 mm and 100 μm. Imaging parameters: Laser at 808 nm, power at 75.5 mW·cm^-2^, exposure time of 50 ms, with a 1000 nm long-pass filter.

We then investigated the ability of IR788-Crizotinib-based NIR-II imaging to detect cervical metastatic lymph nodes in OSCC. Because superficial cervical lymph nodes are anatomically accessible, NIR-II fluorescence imaging is well suited to both preoperative mapping and intraoperative navigation. To establish a relevant preclinical model, the luciferase-expressing human OSCC cell line Cal27-Luc was orthotopically implanted into the tongues of mice, resulting in metastatic spread to cervical lymph nodes. After cervical lymph node metastases were confirmed by bioluminescence imaging (BLI), mice received IR788-Crizotinib and were subjected to NIR-II fluorescence imaging to assess the probe’s ability to detect metastatic lymph nodes (**Figure 6A**).

To define the optimal imaging window, we first performed a time-course study in five OSCC-bearing mice with IVIS-confirmed cervical lymph node metastases. Because skin-associated background fluorescence remained high at earlier time points, the second pair of superficial cervical lymph nodes could not be clearly visualized before 24 h. Accordingly, only the first pair of larger superficial cervical lymph nodes was analyzed in this initial experiment. Signal-to-background ratios (SBRs) were measured at 8, 10, 12, and 24 h after probe injection. A total of 10 lymph nodes were collected after imaging, of which 7 were pathologically confirmed as metastatic and 3 as normal. SBR in metastatic lymph nodes increased over time, rising from 1.48 ± 0.09 at 8 h to 2.01 ± 0.29 at 24 h and further to 2.58 ± 0.25 after skin removal. By contrast, SBR in normal lymph nodes peaked at 12 h (1.54 ± 0.08) and remained similar at 24 h (1.53 ± 0.13). At both 24 h post-injection and after skin removal, metastatic lymph nodes showed significantly higher SBR than normal lymph nodes (2.01 ± 0.29 vs. 1.53 ± 0.13; 2.58 ± 0.25 vs. 1.49 ± 0.06; *** *p* < 0.001, **** *p* < 0.0001; **Figure S37**). These data support 24 h after injection as the optimal imaging window for IR788-Crizotinib-based detection of metastatic lymph nodes.

We next validated these findings in an independent cohort of five OSCC-bearing mice imaged at 24 h after injection. At this time point, reduced background fluorescence enabled visualization of all four superficial cervical lymph nodes in each mouse. Representative BLI and NIR-II fluorescence images are shown in **Figure 6B**. A total of 20 superficial cervical lymph nodes were harvested, including 5 metastatic and 15 normal nodes by pathology. H&E staining and immunohistochemistry showed c-Met-positive tumor nests in metastatic lymph nodes, whereas normal lymph nodes showed minimal c-Met staining (**Figure 6C**). Consistent with this, metastatic lymph nodes exhibited higher SBR than normal lymph nodes both at 24 h and after skin removal (1.84 ± 0.19 vs. 1.38 ± 0.14; 2.55 ± 0.42 vs. 1.66 ± 0.26; ** *p* < 0.01, **** *p* < 0.0001; **Figure 6D**). ROC analysis further demonstrated strong diagnostic performance: at 24 h, an SBR cut-off of 1.55 yielded 100% sensitivity and 86.7% specificity, with an AUC of 0.980 (**Figure 6E**), whereas after skin removal, a cut-off of 1.97 yielded 100% sensitivity and 93.3% specificity, with an AUC of 0.987 (**Figure 6F**). Notably, NIR-II fluorescence imaging detected metastatic foci as small as 342 μm (**Figure 6G**). These results demonstrate that IR788-Crizotinib-based NIR-II imaging enables sensitive identification of cervical metastatic lymph nodes in OSCC and supports its further evaluation for nodal mapping and fluorescence-guided navigation.

### IR788-Crizotinib-guided NIR-II surgery in OSCC and metastatic lymph node models

**Figure 7:**
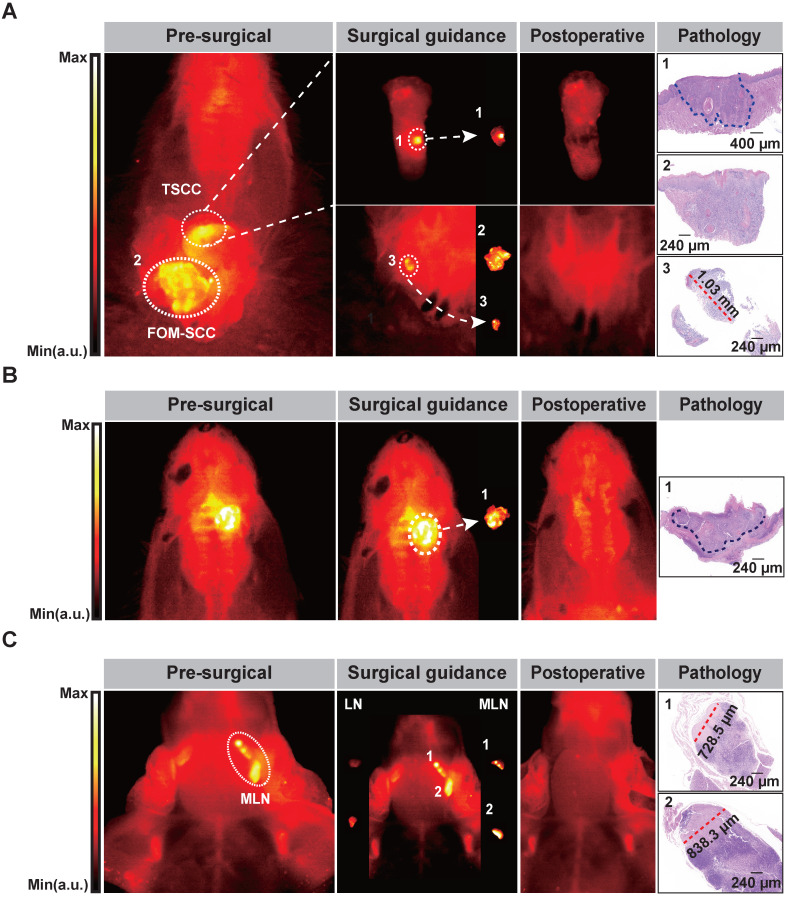
** IR788-Crizotinib-based NIR-II fluorescence-guided resection of OSCC and cervical lymph node dissection. (A-C)** Representative NIR-II fluorescence images acquired 24 h after intravenous injection of IR788-Crizotinib, showing fluorescence-guided resection of OSCC lesions and dissection of cervical metastatic lymph nodes. For each case, the panels are arranged as follows: fluorescence image of the primary lesion or lymph node (first column), fluorescence-guided resection/dissection (second column), post-resection fluorescence image (third column), and H&E staining of the excised fluorescent tissues (fourth column). **(A)** Floor-of-mouth squamous cell carcinoma (FOM-SCC) and tongue squamous cell carcinoma (TSCC). A-3 shows a residual FOM-SCC focus detected after the initial resection. **(B)** Hard palate squamous cell carcinoma (HPSCC). **(C)** Cervical metastatic lymph nodes (MLNs). The blue dashed lines indicate the histological tumor boundaries. Scale bars, 400 μm and 240 μm. Imaging parameters: Laser at 808 nm, power at 75.5 mW·cm^-2^, exposure time of 50 ms, with a 1000 nm long-pass filter.

Finally, we evaluated the feasibility of IR788-Crizotinib-guided NIR-II imaging in surgical settings. To approximate clinically relevant conditions, we included 4-NQO-induced OSCC models arising at common oral sites together with a cervical lymph node metastasis model. Following systemic administration, primary tumors and metastatic lymph nodes exhibited clear intraoperative fluorescence, whereas adipose tissue, muscle, normal mucosa, and non-metastatic lymph nodes showed low background signal, facilitating intraoperative discrimination of malignant lesions (**Figure 7A-C**). Positive contrast was observed across tumors arising at different anatomical sites, with mean TBRs of approximately 3.1 for tongue, floor-of-mouth, and hard palate SCC lesions, and a mean SBR of approximately 2.7 for metastatic lymph nodes.

NIR-II imaging improved detection of residual lesions and delineation of tumor boundaries that were difficult to appreciate under white light. In the floor-of-mouth SCC case, fluorescence revealed a residual tumor focus measuring approximately 1.03 mm in diameter after the initial resection. In the tongue and hard palate SCC cases, NIR-II imaging more clearly delineated tumor margins (**Figure 7A-B**). These findings were supported by histology. In the tongue and hard palate SCC cases, H&E sections confirmed complete resection with negative margins. In the floor-of-mouth SCC case, H&E staining of the surgical bed after NIR-II-guided re-excision showed no residual tumor cells (**Figure S38**). Immunohistochemical staining of the resected lesions is provided in **Figure S39**. In the lymph node dissection model, NIR-II imaging visualized both normal and metastatic cervical lymph nodes and identified metastatic nodes containing foci as small as 700-800 μm, thereby facilitating targeted excision while helping to avoid unnecessary removal of normal nodes (**Figure 7C**). In summary, these results point to that IR788-Crizotinib can support intraoperative fluorescence guidance for both primary tumor resection and cervical lymph node dissection in OSCC.

## Discussion and Conclusion

We developed the c-Met-targeted fluorescent probe IR788-Crizotinib and combined it with an NIR-II imaging platform to enable integrated visualization of OSCC progression, from high-risk oral lesions to primary tumors and metastatic lymph nodes. Within a single imaging framework, this strategy supported lesion stratification, delineation of tumor extent, and intraoperative identification of nodal metastases after one systemic administration. Although IR788-Crizotinib, like ICG, emits predominantly in the NIR-I range with signal extending beyond 1000 nm, its advantage in this study lay in its superior effective imaging performance under the applied detection conditions, rather than in absolute spectral classification alone.

This behavior likely reflects preserved optical output after conjugation, together with improved photostability and stronger depth-dependent contrast. Although IR788-Crizotinib did not have the highest quantum yield among the tested fluorophores, it retained stronger signal under prolonged 808 nm irradiation and showed better penetration-related contrast than IR788, ICG, and ICG-Crizotinib. In our system, conjugation to the ICG scaffold was associated with reduced fluorescence efficiency and poor deep-tissue contrast, consistent with previous reports showing that fluorophore chemistry can strongly influence the imaging behavior of bioconjugates and that ICG-based conjugates may be particularly sensitive to modification 31, 32. These findings suggest that targeted probe performance depends not only on target affinity, but also on how conjugation reshapes fluorophore photophysics and biodistribution. In this context, the IR788 scaffold appeared more tolerant of conjugation than ICG in our study, which may partly explain the superior performance of IR788-Crizotinib across multiple *in vivo* settings.

Diagnosis of OSCC and oral epithelial dysplasia still relies largely on histopathological evaluation of biopsy specimens. Although this remains the clinical standard, it is constrained by sampling bias, repeated procedures and imperfect follow-up adherence. Field cancerization and skip lesions further complicate management, as severe dysplasia or malignant transformation may arise in areas that appear clinically unremarkable 33-36. Non-targeted adjuncts, including toluidine blue, methylene blue, and Lugol’s iodine, can assist lesion detection, but their relatively high false-positive rates and limited specificity reduce diagnostic reliability 37. Together, these limitations argue for a non-invasive, biologically informative approach to early detection and risk-directed intervention.

In previous studies, Reiner *et al.* used PARPi-FL, a green fluorescent probe targeting PARP1, for early oral cancer screening through topical application and demonstrated efficacy in Phase I studies 5, 18, 38. In our earlier work, we developed cMBP-ICG, a NIR probe targeting c-Met, which detected severe dysplasia and OSCC via topical application with 91% accuracy in a 50-patient clinical trial 25, 26. Although targeted fluorescence imaging has shown promise in oral lesion detection, but most previous approaches have remained in the NIR-I window or relied on topical administration. While these methods can be effective, they may be limited by shallow penetration, higher tissue autofluorescence, incomplete mucosal contact, and nonspecific retention on rough or ulcerated surfaces. Against this background, the present study extends previous work in two directions: it uses systemic delivery rather than topical exposure and exploits effective signal detection beyond 1000 nm to improve *in vivo* contrast.

In this study, we established a 4-NQO-induced spontaneous oral carcinogenesis model, successfully recapitulating the process of human oral mucosal carcinogenesis 26, 39, 40. During this process, c-Met expression increased with the severity of dysplasia, peaking in severe dysplasia and OSCC. Consistent with this, IR788-Crizotinib-based imaging distinguished high-risk from low-risk lesions with high sensitivity and high overall accuracy. Notably, the single moderate dysplasia lesion misclassified as high-risk showed strong c-Met expression, suggesting that targeted molecular imaging may reveal biologically relevant alteration before overt structural progression becomes apparent 41. These observations support c-Met-targeted NIR-II imaging not simply as a lesion-visualization tool, but as an aid to biologically informed biopsy selection.

Surgical margin status remains a major determinant of outcome in OSCC 42-44. Although frozen section analysis is widely used intraoperatively, sampling limitations and restricted tissue coverage can lead to discordance with final pathology 45-47. Fluorescence-guided surgery offers a complementary way to visualize tumor extent in real time during resection. In our study, IR788-Crizotinib supported delineation of tumor extent in both xenograft and more clinically relevant spontaneous models. In the 4-NQO-induced model, NIR-II fluorescence guidance enabled complete resection of multifocal lesions despite irregular morphology and poorly defined borders under white light, and residual tumor foci of approximately 1 mm were identified intraoperatively in floor-of-mouth SCC. These support that IR788-Crizotinib-mediated imaging may help reduce the risk of incomplete resection during OSCC surgery by improving intraoperative margin control.

Lymph node metastasis is another major determinant of prognosis in OSCC and remains difficult to assess accurately in both early-stage and advanced disease 13, 48, 49. Occult cervical metastases may be missed in clinically node-negative (cN0) patients, whereas many early-stage patients undergo unnecessary elective neck dissection 48, 50, 51. Recent optical approaches have demonstrated the feasibility of lymph node imaging in OSCC 19, 50, 52, but many rely on antibody-derived targeting strategies that are limited by large molecular size, delayed clearance, and prolonged imaging windows. An ideal lymph node-targeting optical probe should have several key characteristics: robust retention in metastatic lymph nodes, excellent biocompatibility, rapid clearance from normal tissues, high photostability, deep tissue penetration, and precise spatiotemporal resolution 53. Compared to monoclonal antibodies, IR788-Crizotinib (MW: 1814.002 Da) is substantially smaller and, in our system, combined favorable tissue penetration, tumor cell uptake, biosafety, and nodal imaging performance. Furthermore, a single injection enabled simultaneous visualization of primary tumors and metastatic lymph nodes, and metastatic deposits were detected at submillimeter scale. This dual capability is clinically relevant because it supports both resection planning and intraoperative nodal assessment within a single procedure.

While the current study provides valuable insights, it is important to acknowledge its limitations. First, this study used various murine models to simulate the progression of OSCC, from oral mucosal dysplasia to oral cancer and lymph node metastasis. However, it did not involve human subjects, and thus, further research is necessary to determine whether these detection thresholds are applicable to humans. Second, the toxicology of the probe has not been fully validated. While no adverse events were observed in the mice used in this study, and biochemical markers and major organs showed no histological abnormalities, it is important to note that IR788-Crizotinib is primarily cleared through the kidneys, which may lower the risk of hepatic toxicity. Nevertheless, further studies are required to assess its long-term safety in humans.

Overall, IR788-Crizotinib-based imaging provides a targeted framework for real-time visualization of high-risk oral lesions, primary tumors, and metastatic lymph nodes in OSCC. By integrating lesion detection, preoperative assessment, and intraoperative guidance within a single platform, this approach addresses several clinically relevant needs in oral cancer management. These results support further translational evaluation of IR788-Crizotinib for imaging-guided diagnosis and surgery in oral disease.

## Supplementary Material

Supplementary figures and materials and methods.

## Abbreviations

4-NQO: 4-nitroquinoline 1-oxide
AUC: area under the curve
BLI: bioluminescence imaging
BOD: BODIPY
CCK-8: Cell Counting Kit-8
c-Met: mesenchymal-epithelial transition factor
CZ: Crizotinib
FL: fluorescence
FOM-SCC: floor-of-mouth squamous cell carcinoma
H&E: hematoxylin and eosin
HOK: human oral keratinocytes
HPSCC: hard palate squamous cell carcinoma
IHC: immunohistochemistry
IVIS: *in vivo* imaging system
K_D_: equilibrium dissociation constant
LN: lymph node
LNM: lymph node metastasis
MFI: mean fluorescence intensity
MLN: metastatic lymph node
NPV: negative predictive value
NIR: near-infrared
NIR-II: Near-infrared window II
OED: oral epithelial dysplasia
OPMD: oral potentially malignant disorder
OSCC: oral squamous cell carcinoma
PPV: positive predictive value
QY: quantum yield
ROC: receiver operating characteristic
ROI: region of interest
SBR: signal-to-background ratio
SCC: squamous cell carcinoma
SPR: surface plasmon resonance
TBR: tumor-to-background ratio
TMR: tumor-to-muscle ratio
TSCC: tongue squamous cell carcinoma
TSR: tumor-to-skin ratio
WL: white light
