# Supplementary material for: c-Met-targeted NIR-II imaging for precision management of oral squamous cell carcinoma and premalignant lesions
Source: Theranostics. 2026 May 29;16(13):7178–95. doi: 10.7150/thno.132350 (PMC13294983; doi:10.7150/thno.132350)
Supplement: Supplementary file 1 — Supplementary figures and materials and methods. [file thnov16p7178s1.pdf]

Supplementary Materials

c-Met-targeted NIR-II imaging for precision management of oral squamous cell carcinoma and premalignant lesions

Table of contents

General materials and instruments....4

Synthesis and characterization....5

Scheme 1. The synthetic route of IR788-Crizotinib....5
Scheme 2. The synthetic route of ICG-Crizotinib....6
Scheme 3. The synthetic route of BODIPY-Crizotinib....6
Synthesis of Crizotinib-PEG8-NHBoc....7
Synthesis of Crizotinib-PEG8-NH_2....7
Synthesis of IR-788....7
Synthesis of IR788-Crizotinib....8
Synthesis of ICG-Crizotinib....8
Synthesis of BODIPY-Crizotinib....9

Supplementary Methods....10

Fluorescence quantum yield measurement....10
Photostability evaluation under continuous 808-nm irradiation....10
Evaluation of optical stability of IR788-Crizotinib in different pH....10
In vitro penetration assay in an Intralipid phantom....11
In vivo penetration assay....11
Western blot....11
Immunohistochemistry....11
Surface plasmon resonance....12
Toxicity evaluation of IR788-Crizotinib.....12

NMR and MS spectra....13

Figure S1. 13
Figure S2. 14
Figure S3. 15
Figure S4. 16
Figure S5. 17
Figure S6. 18
Figure S7. 19
Figure S8. 20
Figure S9. 21
Figure S10. 22
Figure S11. 23
Figure S12. 24
Figure S13. 25
Figure S14. 26

Supplementary Figures 27

Figure S15. 27
Figure S16. 28
Figure S17. 29
Figure S18. 30
Figure S19. 31
Figure S20. 32
Figure S21. 33
Figure S22. 34
Figure S23. 35
Figure S24. 36
Figure S25. 37
Figure S26. 38
Figure S27. 39
Figure S28. 40
Figure S29. 41

Figure S30......42
Figure S31......43
Figure S32......44
Figure S33......45
Figure S34......46
Figure S35......47
Figure S36......48
Figure S37......49
Figure S38......50
Figure S39......51

General materials and instruments

Commercially available solvents and reagents were used without further purification unless otherwise specified. ^{1}H and ^{13}C NMR spectra were acquired on Bruker 400, 500, or 600 MHz spectrometers in CDCl3 or DMSO-*d*6, with Chemical shifts (δ) are reported in ppm relative to internal TMS. Multiplicities are denoted as s, singlet; d, doublet; t, triplet; q, quartet; m, multiplet; and dd, doublet of doublets. LC-MS analysis was conducted on a Waters SQ Detector 2 (SQD2), and preparative HPLC was performed using a Shimadzu LC-20AR system equipped with PDA detection. UV-vis-NIR absorption spectra were recorded on a Shimadzu UV-2600 spectrophotometer using Shimadzu Common Container software, version 2.70, whereas fluorescence emission spectra were collected with a home-built NIR-II fluorescence spectrometer. Deionized water was obtained from a Millipore Milli-DI water purification system (Merck). NIR-II fluorescence images were acquired using an *in vivo* imaging system equipped with a water-cooled InGaAs SWIR camera (NIRvana-640, Teledyne Princeton Instruments; 640 × 512 pixels), and images were background-corrected using LightField software, version 6.11. 4.

Synthesis and characterization

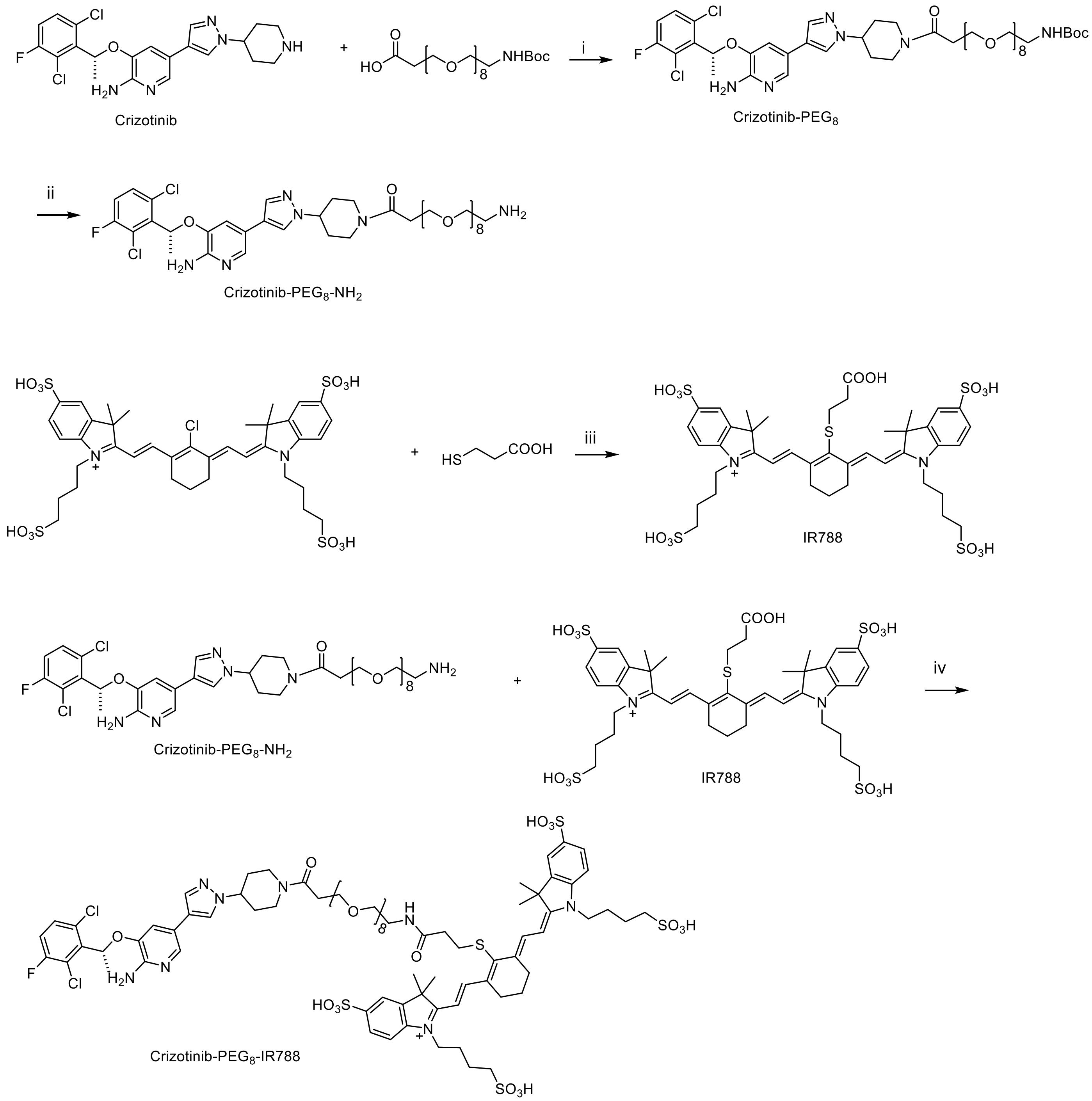

Scheme 1. The synthetic route of IR788-Crizotinib. Reactions and reagents: i) TBTU, DIPEA, DMF; ii) TFA/DCM; iii) DIPEA, dry DMSO; iv) TBTU, DIPEA, dry DMSO.

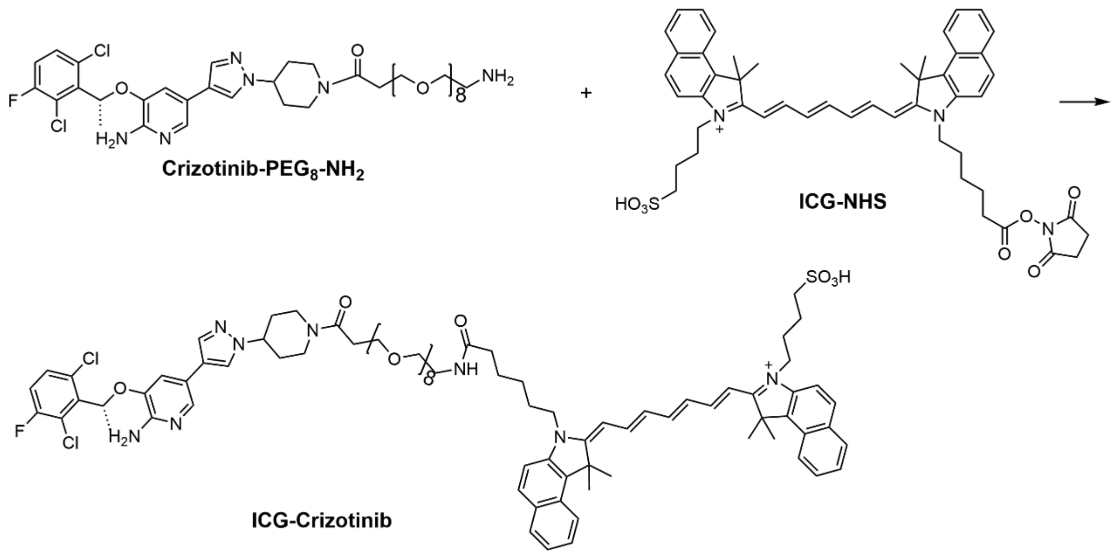

Scheme 2. The synthetic route of ICG-Crizotinib. Reaction and reagents: DIPEA, dry DMSO.

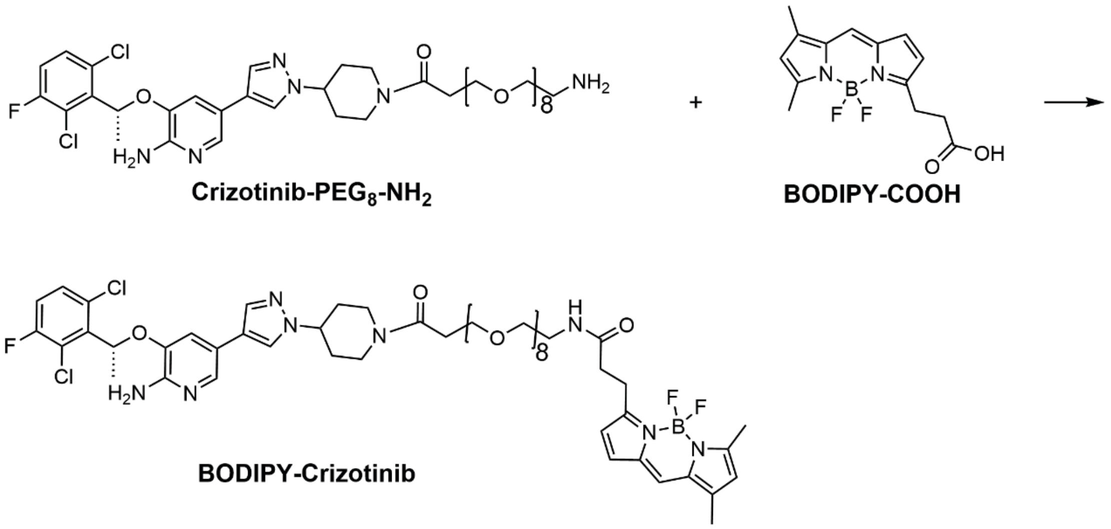

Scheme 3. The synthetic route of BODIPY-Crizotinib. Reaction and reagents: TBTU, DIPEA, dry DMF.

Synthesis of Crizotinib-PEG8-NHBoc

A solution of crizotinib (100 mg, 0.22 mmol) in DMF was treated with Boc-N-amido-PEG8-acid (132 mg, 0.24 mmol), TBTU (92 mg, 0.28 mmol), and DIPEA (86 mg, 0.66mmol) under a nitrogen atmosphere. The reaction mixture was stirred at room temperature overnight. After the reaction completed, water was added to quench the reaction. The mixture was extracted with ethyl acetate, and the combined organic layers were dried over anhydrous sodium sulfate, filtered and concentrated under vacuum. The crude product was purified by silica gel column chromatography to afford the compound **Crizotinib-PEG8-NHBoc** as a colorless oil 150 mg (yield, 69%). ^{1}H NMR (600 MHz, Chloroform-*d*) δ 7.72 (d, *J* = 1.8 Hz, 1H), 7.58 (s, 1H), 7.53 (s, 1H), 7.34 (dd, *J* = 8.9, 4.7 Hz, 1H), 7.13 – 7.07 (m, 1H), 6.92 (d, *J* = 1.8 Hz, 1H), 6.11 (q, *J* = 6.7 Hz, 1H), 5.32 (s, 2H), 5.18 (s, 1H), 4.75 (d, *J* = 13.6 Hz, 1H), 4.35 (tt, *J* = 11.3, 4.0 Hz, 1H), 4.08 (d, *J* = 13.4 Hz, 1H), 3.83 (t, *J* = 6.7 Hz, 2H), 3.68 – 3.64 (m, 28H), 3.56 (t, *J* = 5.2 Hz, 2H), 3.33 (q, *J* = 5.4 Hz, 2H), 3.23 (ddd, *J* = 14.3, 12.0, 2.7 Hz, 1H), 2.80 (d, *J* = 13.1 Hz, 1H), 2.71 (q, *J* = 6.8 Hz, 2H), 2.26 – 2.17 (m, 2H), 1.98 (ddt, *J* = 24.6, 12.3, 6.2 Hz, 2H), 1.89 (d, *J* = 6.6 Hz, 3H), 1.46 (s, 9H).^{13}C NMR (126 MHz, Chloroform-*d*) δ169.49, 158.54, 156.56, 156.05, 148.60, 140.14, 136.51, 135.95, 132.88, 130.03, 128.93, 128.90, 122.96, 122.11, 121.96, 119.52, 118.90, 117.02, 116.84, 115.50, 79.11, 77.31, 77.06, 76.81, 72.77, 70.53, 70.48, 70.45, 70.26, 70.22, 67.46, 59.07, 44.53, 40.51, 40.36, 33.61, 32.84, 31.91, 28.43, 18.87. LC-MS Calcd for: $C_{45}H_{67}Cl_{2}FN_{6}O_{12}$: 973.96. Found: 974.06 $[M+H]^{+}$.

Synthesis of Crizotinib-PEG8-NH$_2$

Compound **Crizotinib-PEG8-NHBoc** was dissolved in dichloromethane, followed by addition of trifluoroacetic acid (V_{DCM}: V_{TFA} = 3:1). The mixture was stirred at room temperature for 1 hour. Then the solvent was concentrated under vacuum to afford a brown oil which was used for the next step without further purification. LC-MS Calcd for: $C_{40}H_{59}Cl_{2}FN_{6}O_{10}$: 872.37. Found: 873.87 $[M+H]^{+}$.

Synthesis of IR-788

To a solution of tetrasulfonyl chloride Cy7 (10 mg, 0.011 mmol) in anhydrous dimethyl sulfoxide (1 mL) were added triethylamine (3 μL) and mercaptopropionic acid (10 μL) under a nitrogen atmosphere. Then the reaction mixture was stirred at room temperature in the dark. After the reaction finished, an excess of ethyl acetate was added to the reaction mixture to precipitate a green solid. The precipitate was collected and dissolved in a water. Shimadzu LC-20AR high-performance liquid chromatography (HPLC) system

(Shimadzu Corporation, Japan), XBridge® BEH C18 OBD™ Preparative Column (10 mm × 250 mm, 5 μm), gradient elution starting from 10% acetonitrile and ending up with 50% acetonitrile (in water with 0.1%TFA), 3 mL/min flow rate, 254 nm and 780 nm detection wavelength was used to purify the reaction. The product was obtained as a green solid 5 mg (yield: 53%). ^{1}H NMR (600 MHz, DMSO-d_6) δ 8.69 (d, J = 13.8 Hz, 2H), 7.74 (s, 2H), 7.64 (dd, J = 8.2, 1.6 Hz, 2H), 7.36 (d, J = 8.3 Hz, 2H), 6.35 (d, J = 14.2 Hz, 2H), 4.19 – 4.13 (m, 4H), 2.99 (t, J = 7.0 Hz, 2H), 2.66 (t, J = 6.1 Hz, 4H), 2.53 (t, J = 7.2 Hz, 4H), 1.84 – 1.77 (m, 6H), 1.74 (d, J = 7.5 Hz, 6H), 1.68 (s, 12H). ^{13}C NMR (126 MHz, DMSO-*d6*) δ 172.79, 172.39, 155.44, 145.36, 145.16, 142.80, 140.80, 134.07, 126.73, 120.20, 110.95, 102.27, 51.16, 49.22, 44.17, 34.97, 32.72, 27.84, 26.43, 26.20, 22.82, 20.99. LC-MS Calcd for: $C_{41}H_{53}N_2O_{14}S_5^+$: 957.21. Found: 957.94 $[M]^+$.

Synthesis of IR788-Crizotinib

Crizotinib-PEG8-NH₂ (18 mg, 0.02 mmol) and **IR-788** (10 mg, 0.01 mmol) were dissolved in anhydrous DMSO. Then, TBTU (6.7 mg, 0.02 mmol) and DIPEA (6.7 mg, 0.05 mmol) were added to the mixture. The reaction was stirred in the dark under nitrogen overnight. After the reaction finished, the crude product was precipitated with excess ethyl acetate as a green solid, filtered, redissolved in water, and purified by semi-preparative HPLC using the same method as for **IR-788**. Finally, the product was obtained as a green solid 5 mg (yield, 26%). MS (ESI) *m/z* 907.65 $[M+H]^{2+}$, 917.80 $[M+Na]^{2+}$; HRMS 907.7838 $[M+H]^{2+}$.

Synthesis of ICG-Crizotinib

Crizotinib-PEG8-NH₂ (2.1 mg, 0.002 mmol) and **ICG-NHS** (1 mg, 0.001 mmol) were dissolved in anhydrous DMSO. Then, DIPEA (0.78 mg, 0.006 mmol) was added to the mixture. The reaction was stirred in the dark under nitrogen for 5 h. After the reaction finished, the crude product was precipitated with excess ethyl acetate as a green solid. The precipitate was collected and dissolved in water. Purification was performed using a Shimadzu LC-20AR HPLC system equipped with an XBridge® BEH C18 OBD preparative column (10 mm × 250 mm, 5 μm). A gradient elution was applied from 30% to 80% acetonitrile in water (containing 0.1% TFA) at a flow rate of 3 mL/min. Detection wavelengths were set at 254 nm and 780 nm. The product was obtained as a green solid 1 mg (yield, 52%). MS (ESI) *m/z* 1587 $[M+H]^+$, 794.68 $[M+H]^{2+}$, 804.48 $[M+Na]^{2+}$; HRMS (ESI) 794.3556 $[M+H]^{2+}$.

Synthesis of BODIPY-Crizotinib

The synthesis of **BODIPY-Crizotinib** was performed according to the same method used for **IR788-Crizotinib**. ^{1}H NMR (400 MHz, Chloroform-*d*) δ 7.74 (s, 1H), 7.56 (s, 1H), 7.50 (s, 1H), 7.32 (dd, *J* = 8.8, 4.7 Hz, 1H), 7.11 – 7.04 (m, 2H), 6.88 (s, 2H), 6.37 (s, 1H), 6.30 (d, *J* = 3.8 Hz, 1H), 6.11 (s, 1H), 6.10 – 6.05 (m, 1H), 5.01 (s, 2H), 4.73 (d, *J* = 14.8 Hz, 1H), 4.32 (t, *J* = 11.3 Hz, 1H), 4.05 (d, *J* = 13.9 Hz, 1H), 3.81 (t, *J* = 6.7 Hz, 2H), 3.64 (s, 26H), 3.52 (t, *J* = 4.9 Hz, 2H), 3.47 – 3.42 (m, 2H), 3.29 (t, *J* = 7.5 Hz, 2H), 3.21 (t, *J* = 12.3 Hz, 1H), 2.77 (t, *J* = 12.0 Hz, 1H), 2.69 (t, *J* = 5.5 Hz, 2H), 2.63 (t, *J* = 7.5 Hz, 2H), 2.56 (s, 3H), 2.35 (t, *J* = 6.5 Hz, 1H), 2.25 (s, 3H), 2.22 – 2.13 (m, 2H), 2.00 – 1.91 (m, 2H), 1.87 (d, *J* = 6.6 Hz, 3H). ^{13}C NMR (151 MHz, $CDCl_3$) δ 171.32, 169.04, 159.53, 157.52, 148.42, 143.23, 139.53, 136.33, 135.54, 134.59, 134.12, 132.95, 128.53, 128.50, 127.89, 123.36, 122.43, 119.88, 119.47, 118.53, 117.05, 116.49, 116.33, 114.69, 72.12, 70.11, 70.05, 69.81, 69.43, 67.05, 58.62, 44.12, 41.58, 40.10, 38.85, 35.32, 33.20, 32.45, 31.51, 29.29, 26.61, 24.34, 18.48, 14.52, 10.90. MALDI-TOF Calcd for: $C_{54}H_{72}BCl_2F_3N_8O_{11}$: 1146.47. Found: 1147.80 $[M+H]^+$.

Supplementary Methods

Fluorescence quantum yield measurement

The fluorescence quantum yields of IR788-Crizotinib, ICG-Crizotinib, IR788, and ICG were determined by a relative method using IR-26 in 1,2-dichloroethane as the reference standard. For each compound, a series of diluted solutions was prepared, and the absorbance at 808 nm was maintained below 0.1 to minimize re-absorption effects. Fluorescence emission spectra were collected over the 850-1000 nm range, and the integrated emission intensity was plotted against the corresponding absorbance at 808 nm. Linear fitting was then performed for each sample. The fluorescence quantum yield was calculated from the slope ratio relative to IR-26, with correction for the refractive indices of the solvents.

The fluorescence quantum yield of each sample was calculated according to the following equation:

$$QY_{sample} = QY_{ref} \times \frac{n_{sample}^2}{n_{ref}^2} \times \frac{Slope_{sample}}{Slope_{ref}}$$

where QY_{ref} is the quantum yield of IR-26, QY_{sample} is the quantum yield of the tested sample, $Slope_{sample}$ and $slope_{ref}$ are the slopes obtained from the linear fitting of the sample and IR-26, respectively. n_{sample} is the refractive index of water (1.333), and n_{ref} is the refractive index of 1,2-dichloroethane (1.444).

Photostability evaluation under continuous 808-nm irradiation

Photostability was assessed by exposing IR788-Crizotinib, ICG-Crizotinib, IR788 and ICG solutions (100 µM in 1× PBS) to continuous 808-nm laser irradiation at 75.5 mW cm^{-2} for 30 min. Fluorescence signals were recorded in real-time using an InGaAs short-wave infrared camera equipped with a 1000-nm long-pass emission filter under identical acquisition settings. Signal intensity at each time point was normalized to the initial intensity to quantify fluorescence decay.

Evaluation of optical stability of IR788-Crizotinib in different pH

The pH stability of IR788-Crizotinib was assessed under mildly acidic, neutral and mildly alkaline conditions. The probe was incubated in buffer solutions at pH 6.5, 7.4 and 8.0 for 0.5, 2 and 20 h, after which fluorescence intensity was measured using identical instrumental settings. Absorption spectra were further recorded after 20 h of incubation at each pH to evaluate potential spectral changes.

In vitro penetration assay in an Intralipid phantom

The penetration performance of IR788-Crizotinib, ICG-Crizotinib, IR788 and ICG was evaluated in vitro using a capillary-based Intralipid phantom. Capillary tubes containing each probe solution (100 µM in 1× PBS) were placed beneath a square culture dish, and increasing volumes of 1% Intralipid were added to generate defined phantom depths. The depth from the Intralipid surface to the capillary was calculated by dividing the Intralipid volume by the bottom area of the dish. NIR-II fluorescence images were acquired at each depth using 808-nm excitation at 75.5 mW cm^{-2} and a 1000-nm long-pass emission filter. SBR was calculated to compare penetration performance among the probes.

In vivo penetration assay

To assess tissue penetration *in vivo*, capillary tubes containing IR788-Crizotinib solution (100 µM in 1× PBS) were inserted at defined subcutaneous or intramuscular depths in mice. NIR-II fluorescence images were acquired under the same settings, including 808-nm excitation at 75.5 mW cm^{-2} and a 1000-nm long-pass emission filter. Capillary depth was measured with a vernier caliper, and SBR was analysed as a function of tissue depth.

Western blot

Whole-cell lysates were prepared in RIPA buffer supplemented with protease and phosphatase inhibitors. Total protein concentration was determined using a BCA assay, and 15 µg protein was loaded per lane. Proteins were denatured at 95 °C for 10 min, separated by 10% SDS-PAGE and transferred to PVDF membranes. Membranes were blocked with 5% non-fat milk in TBST for 1 h at room temperature and incubated overnight at 4 °C with anti-c-Met antibody (Abcam, ab51067; 1:1000), followed by HRP-conjugated anti-rabbit secondary antibody (HUABIO, HA1001; 1:10000) for 30 min at room temperature. Signals were developed using ECL reagents and captured with a Tanon-4600 imaging system. Parallel membranes loaded with identical samples were probed with β-Actin antibody (Proteintech, 81115-1-RR; 1:10000) as the loading control. Band intensities were quantified using Image*J* Gel Analyzer and normalized to β-Actin.

Immunohistochemistry

Immunohistochemistry was performed on 3-µm-thick paraffin-embedded sections. Sections were deparaffinized, rehydrated and subjected to citrate-based antigen retrieval at pH 6.0. Endogenous peroxidase

activity and non-specific binding were blocked with 3% hydrogen peroxide and 3% bovine serum albumin, respectively. Sections were incubated with an anti-c-Met rabbit monoclonal antibody (Abcam, ab51067; 1:300) overnight at 4 °C, followed by HRP-conjugated goat anti-rabbit IgG (Abcam, ab205718; 1:5000) for 50 min at room temperature. Staining was developed with DAB substrate, counterstained with Harris haematoxylin, dehydrated and mounted.

Surface plasmon resonance

Surface plasmon resonance (SPR) measurements were performed on a Biacore 8K instrument (Cytiva) at 25 °C using CM5 sensor chips. Recombinant c-Met was immobilized on flow cell 4 (Fc4) by standard amine coupling. The surface was activated with freshly prepared EDC/NHS (200 mM/50 mM) for 420 s at 10 μL/min, followed by injection of c-Met (50 μg/mL in sodium acetate buffer, pH 4.0-5.5) to 5922 response units (RU). Flow cell 3 (Fc3), activated and blocked without ligand, served as the reference surface. Residual active esters were quenched with 1 M ethanolamine-HCl for 300 s at 10 μL/min.

IR788-Crizotinib was diluted in running buffer [1× PBS-P+ (pH 7.4), 5% (v/v) DMSO] and injected over Fc3 and Fc4 at 30 μL/min, with 60 s association and 90 s dissociation phases. Six concentrations ranging from 0.3125 to 10 μM were analysed. The surface was regenerated between injections with 10 mM glycine-HCl (pH 2.0) for 5 min. DMSO solvent correction and double referencing were applied, and sensorgrams were fitted using a 1:1 Langmuir binding model.

Toxicity evaluation of IR788-Crizotinib.

Toxicity of IR788-Crizotinib was assessed in both cell-based and animal experiments. For *in vitro* testing, Cal27-Luc and HOK cells were plated in 96-well plates at 5,000 cells per well (five replicates per condition) and incubated with IR788-Crizotinib at 0.625-10 μM for 24 h. Vehicle-treated wells received the corresponding DMSO concentration. CCK-8 reagent (10 μL; yuuta genes, R03-261B) was then added to each well containing 100 μL culture medium and allowed to react for 2 h at 37 °C. Optical density was read at 450 nm, and cell viability was expressed relative to the vehicle group. For *in vivo* evaluation, mice were given a single tail-vein injection of IR788-Crizotinib at 15 μg/g, equivalent to a 10-fold imaging dose. Control animals received the same volume of PBS. General status and body weight were followed throughout the observation period. Blood was collected on days 14 and 28 for hematology and serum biochemistry, including indices of hepatic and renal function. Major organs were harvested at the study endpoint for H&E staining and histopathological evaluation.

NMR and MS spectra

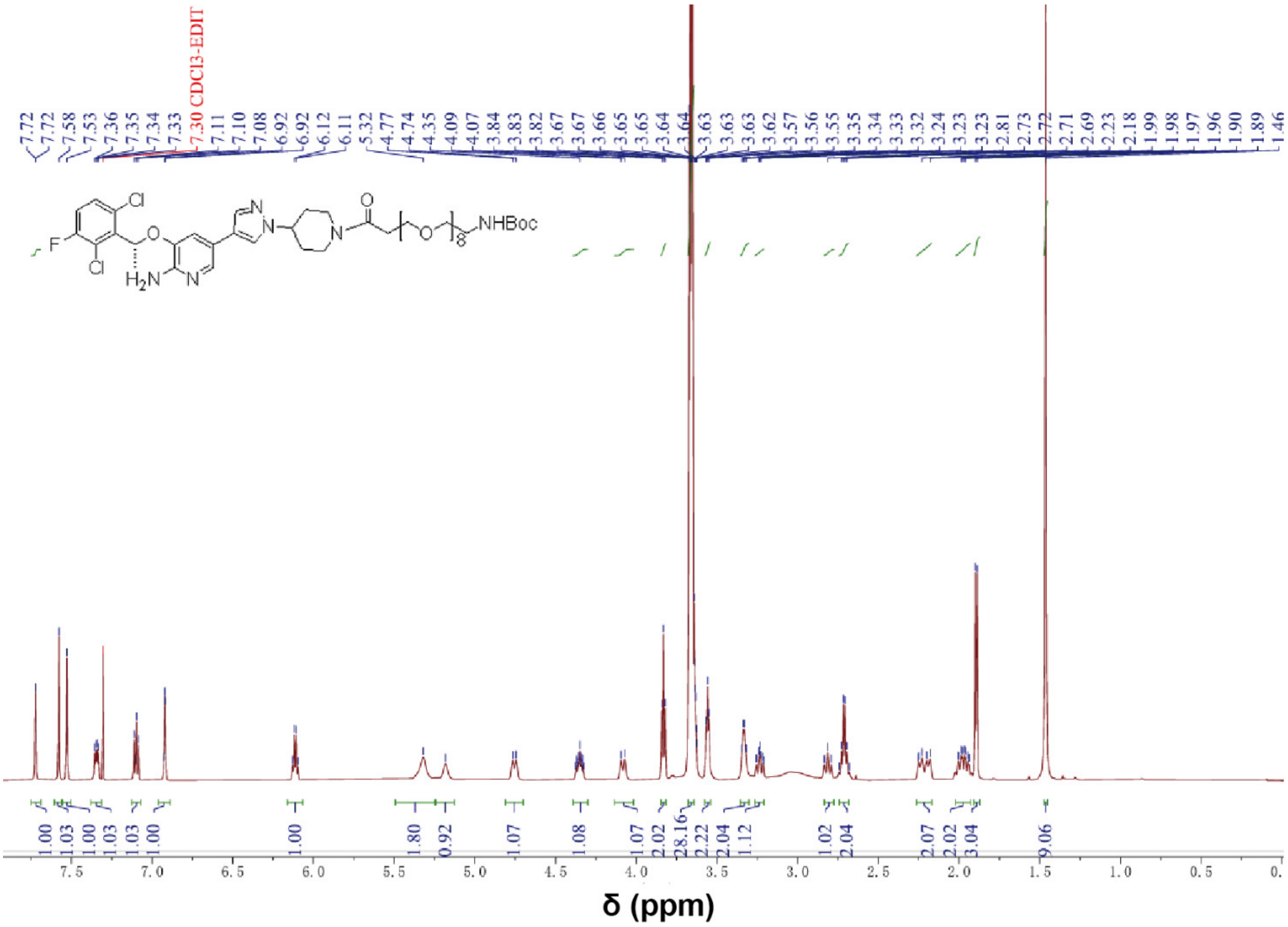

Figure S1.

The ^{1}H NMR (600 MHz, Chloroform-*d*) of Crizotinib-PEG8-NHBoc.

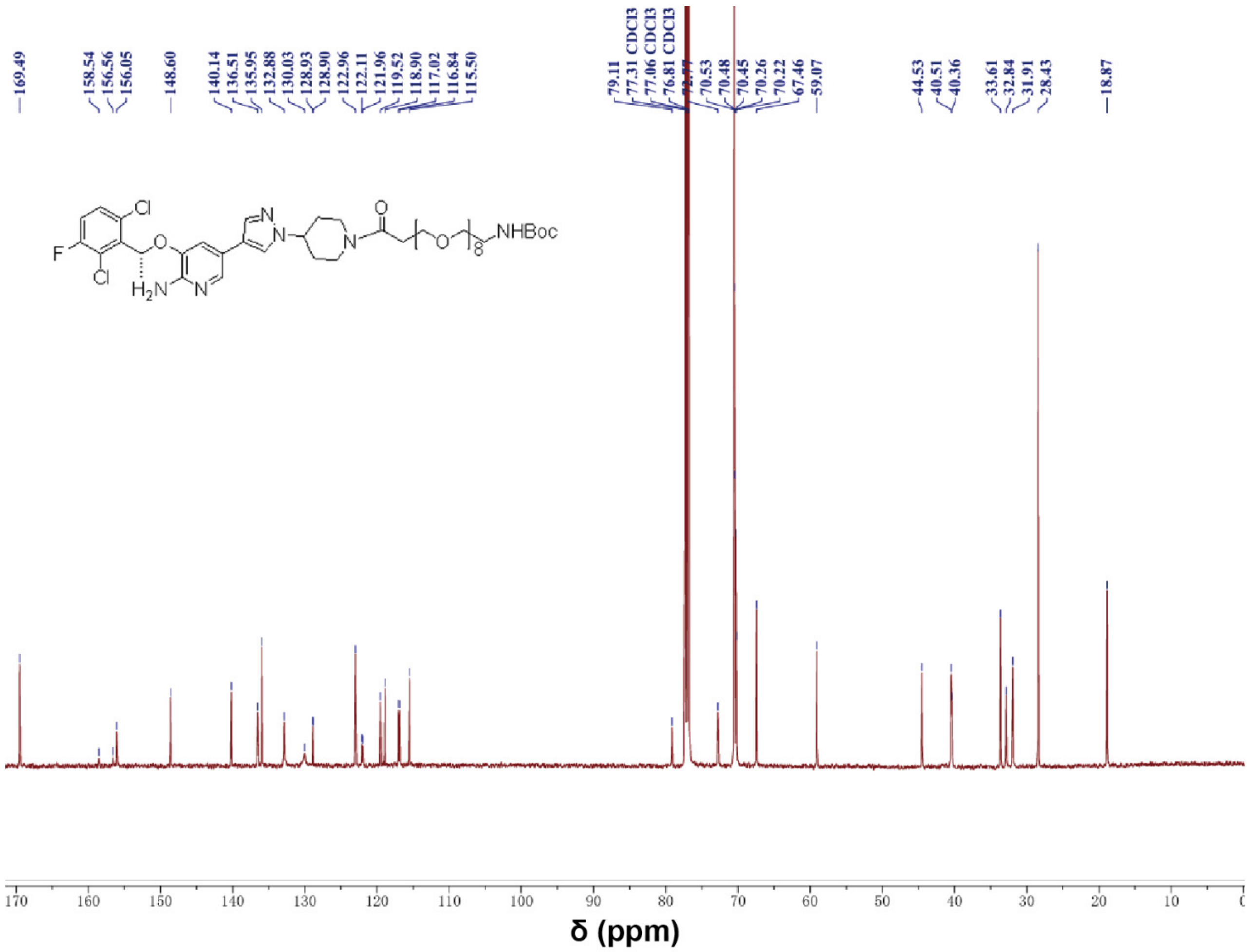

Figure S2.

The ^{13}C NMR (126 MHz, Chloroform-*d*) of Crizotinib-PEG8-NHBoc.

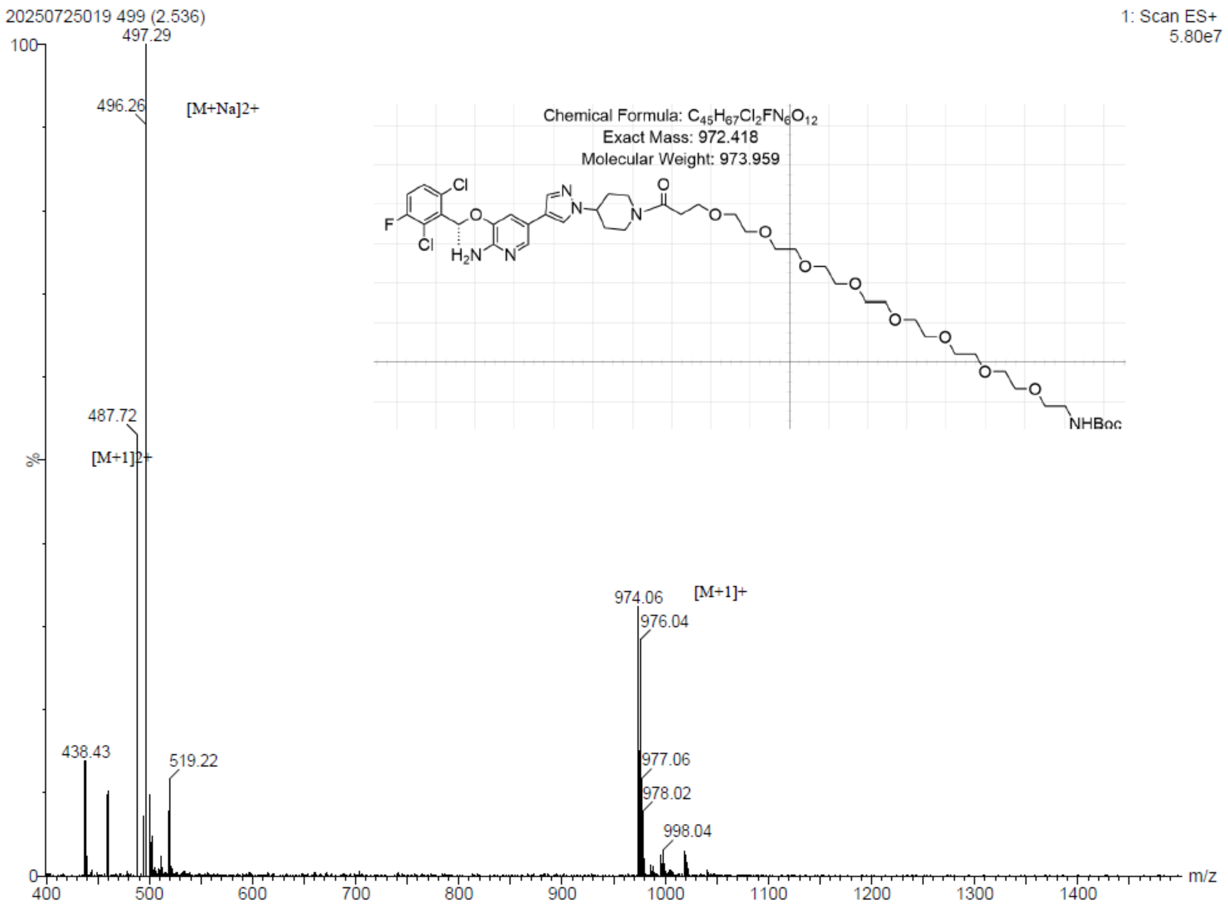

Figure S3.

The MS of Crizotinib-PEG8-NHBoc.

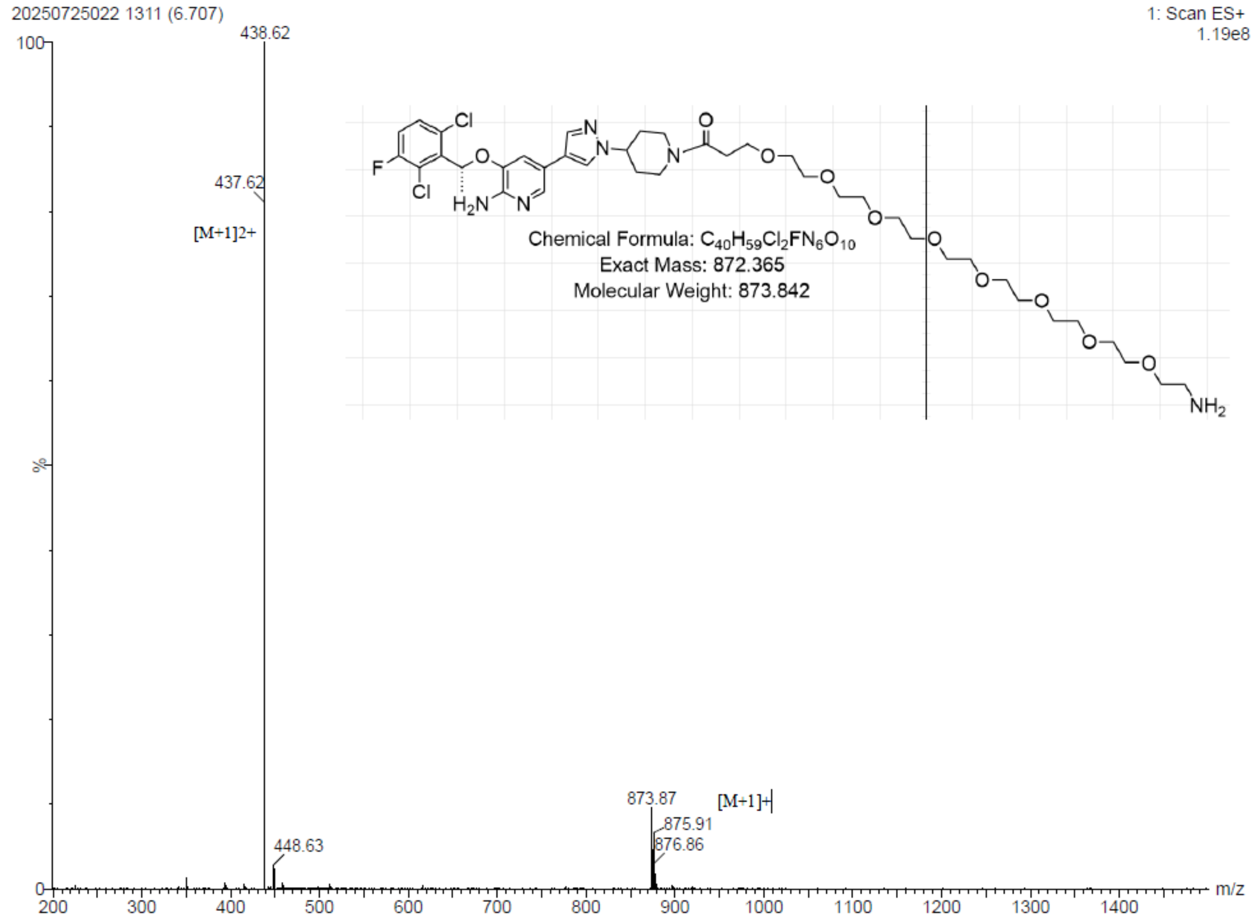

Figure S4.

The MS of Crizotinib-PEG8-NH_2.

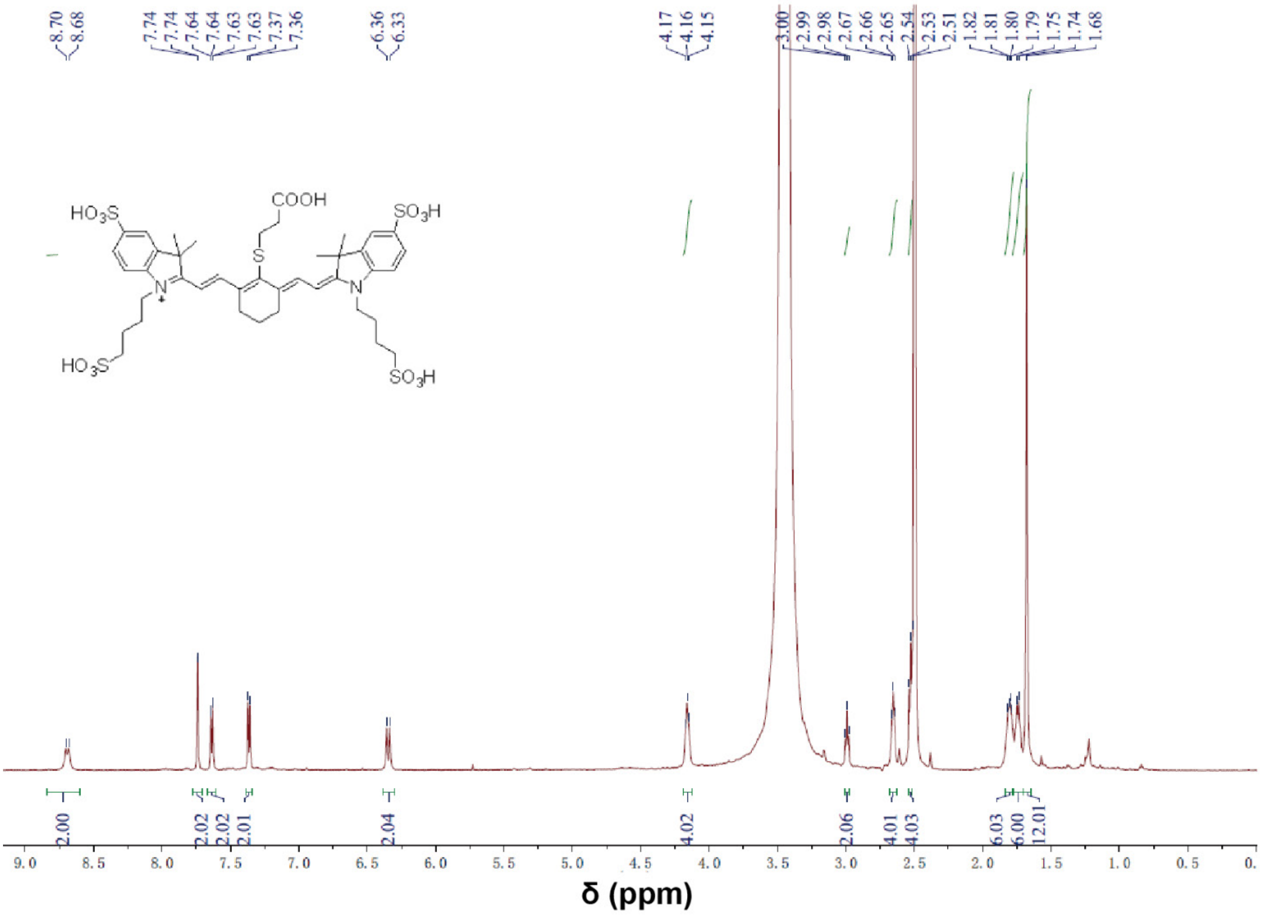

Figure S5.

The ^{1}H NMR (600 MHz, DMSO-d_6) of IR788.

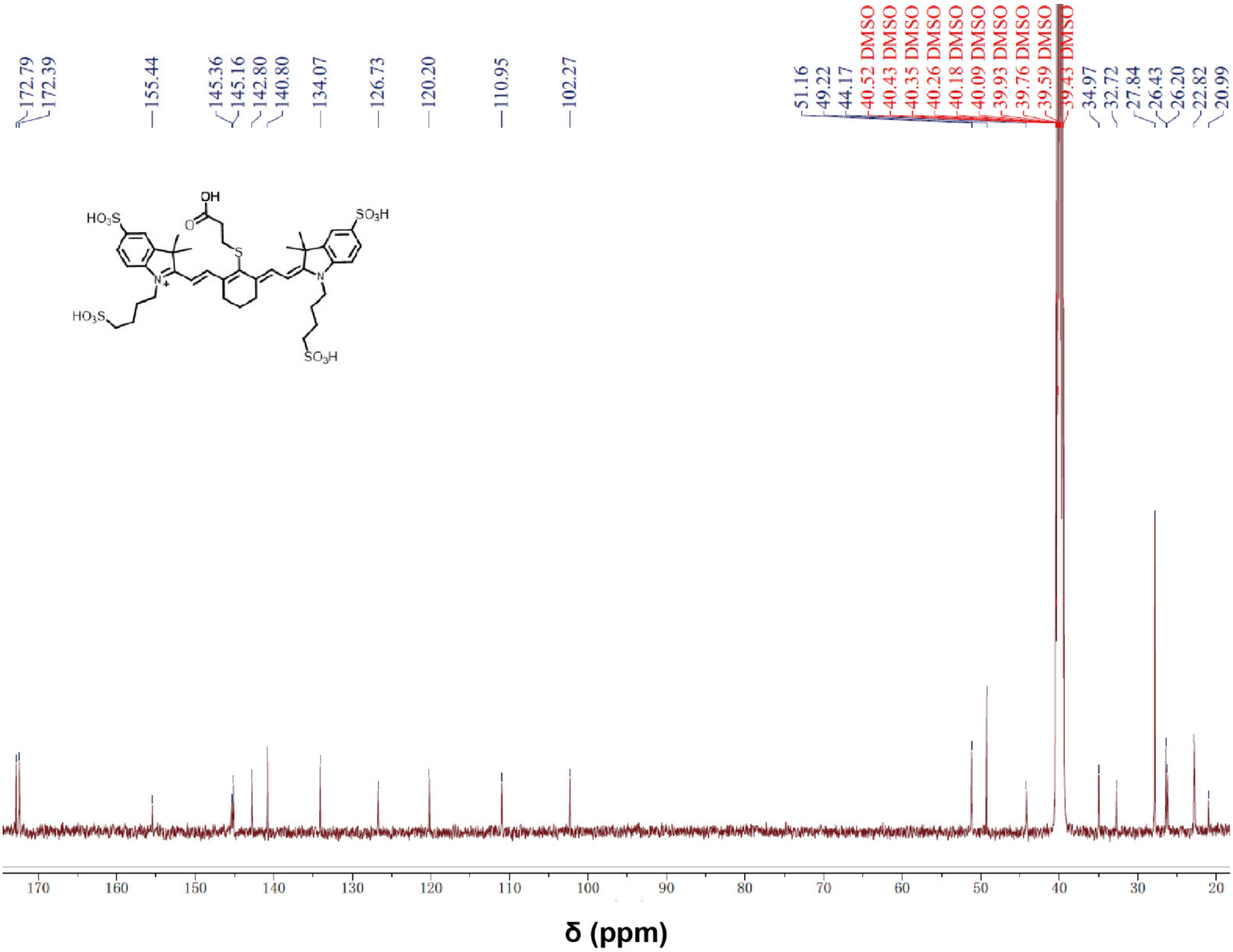

Figure S6.

The ^{13}C NMR (126 MHz, DMSO-d_6) of IR788.

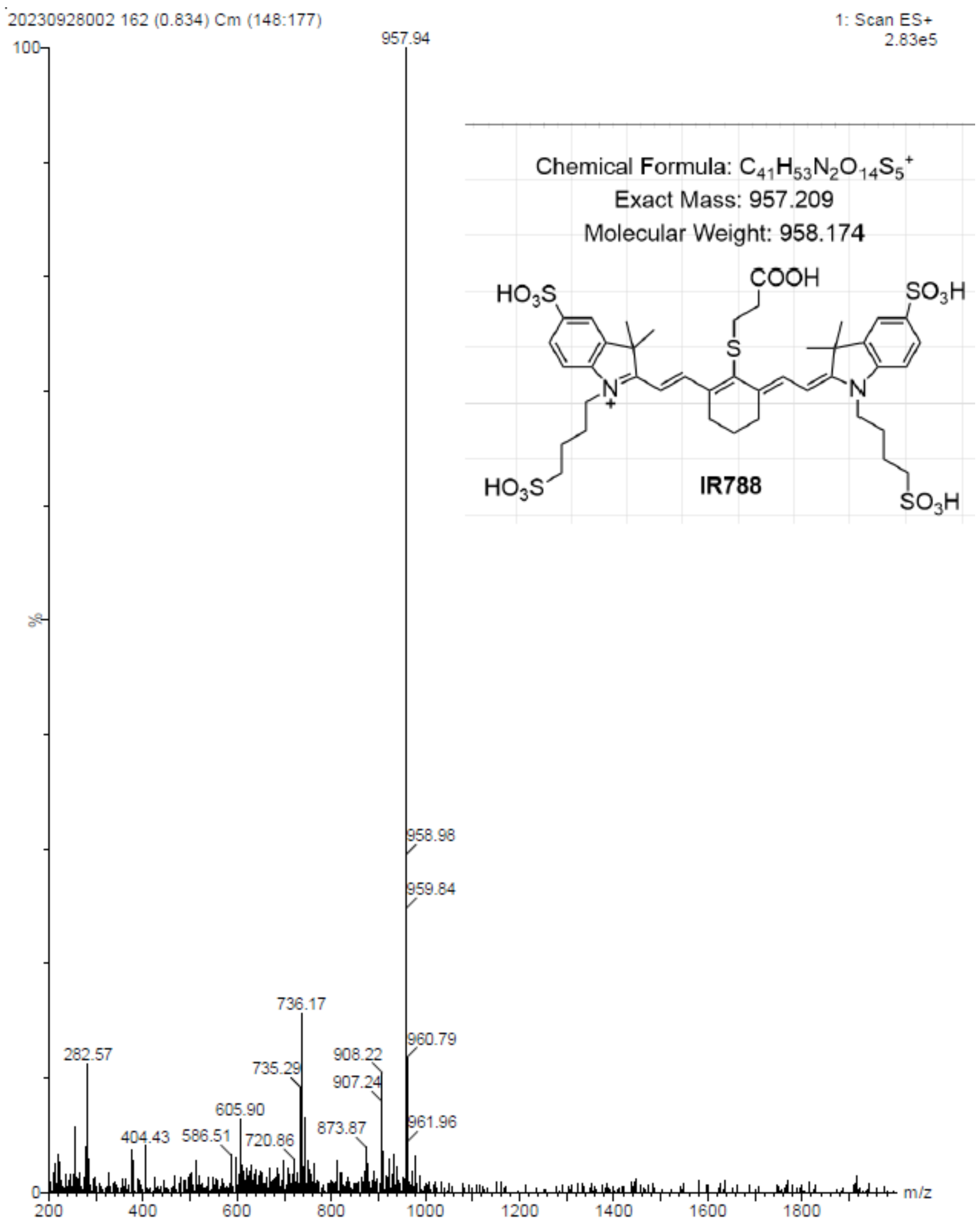

Figure S7.

The MS of IR-788.

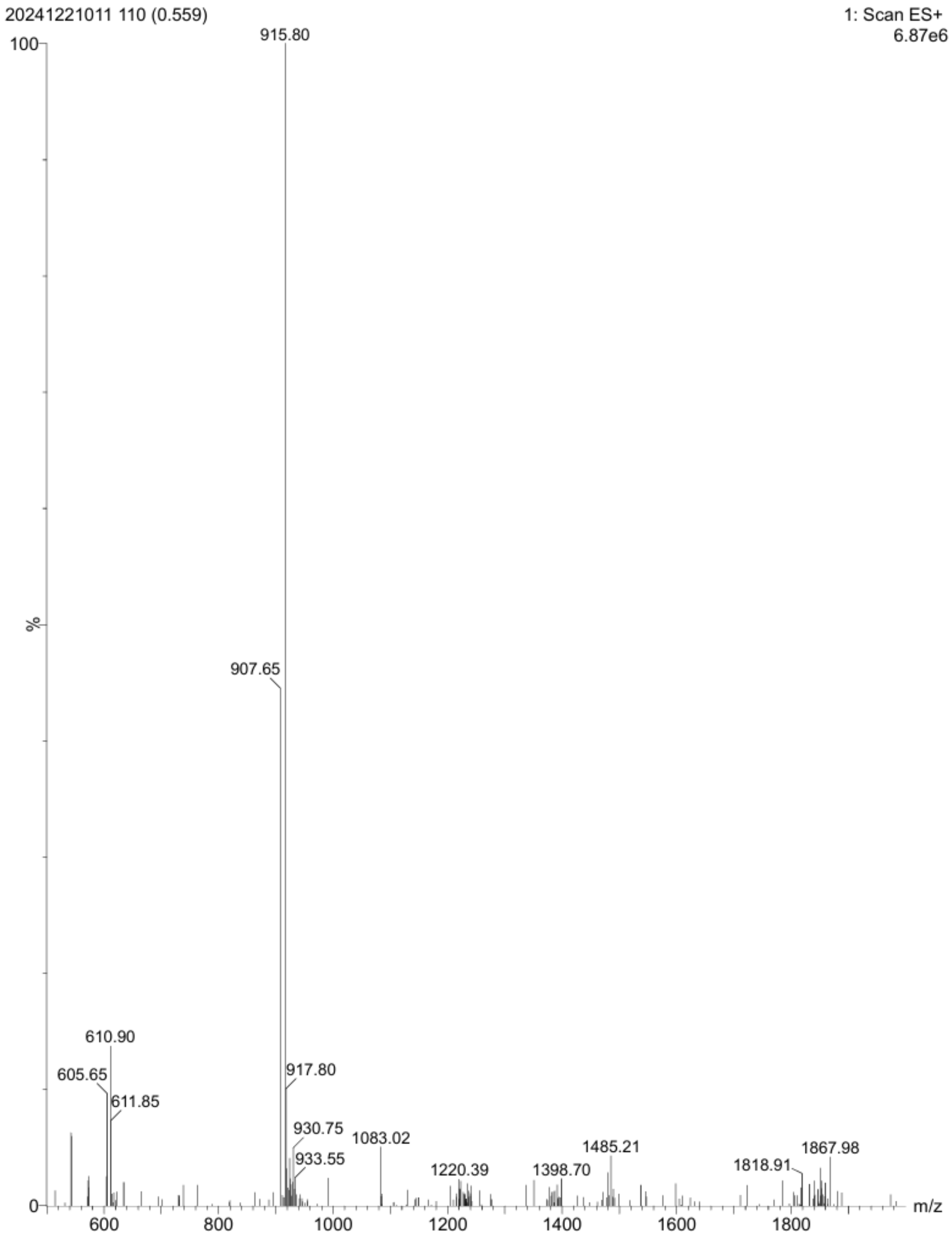

Figure S8.

The MS of IR788-Crizotinib.

Qualitative Analysis Report

**Data Filename**	ESIH202504232.d	**Sample Name**	F9-JAY-87
**Sample ID**		**Position**	P1-B9
**Instrument Name**	Agilent 6520 Q-TOF	**Acq Method**	20160322_MS_ESIH_POS_1min.m
**Acquired Time**	8/21/2025 2:18:27 PM	**DA Method**	ESI-HR-20231114.m
**Comment**	ESIH by fangsu		

User Spectra

**Fragmentor Voltage**	**Collision Energy**	**Ionization Mode**
170	0	ESI

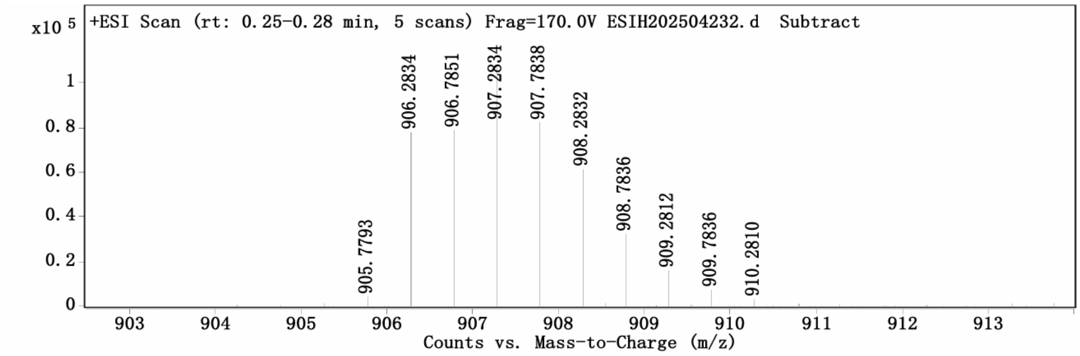

Figure S9.

The HRMS of IR788-Crizotinib.

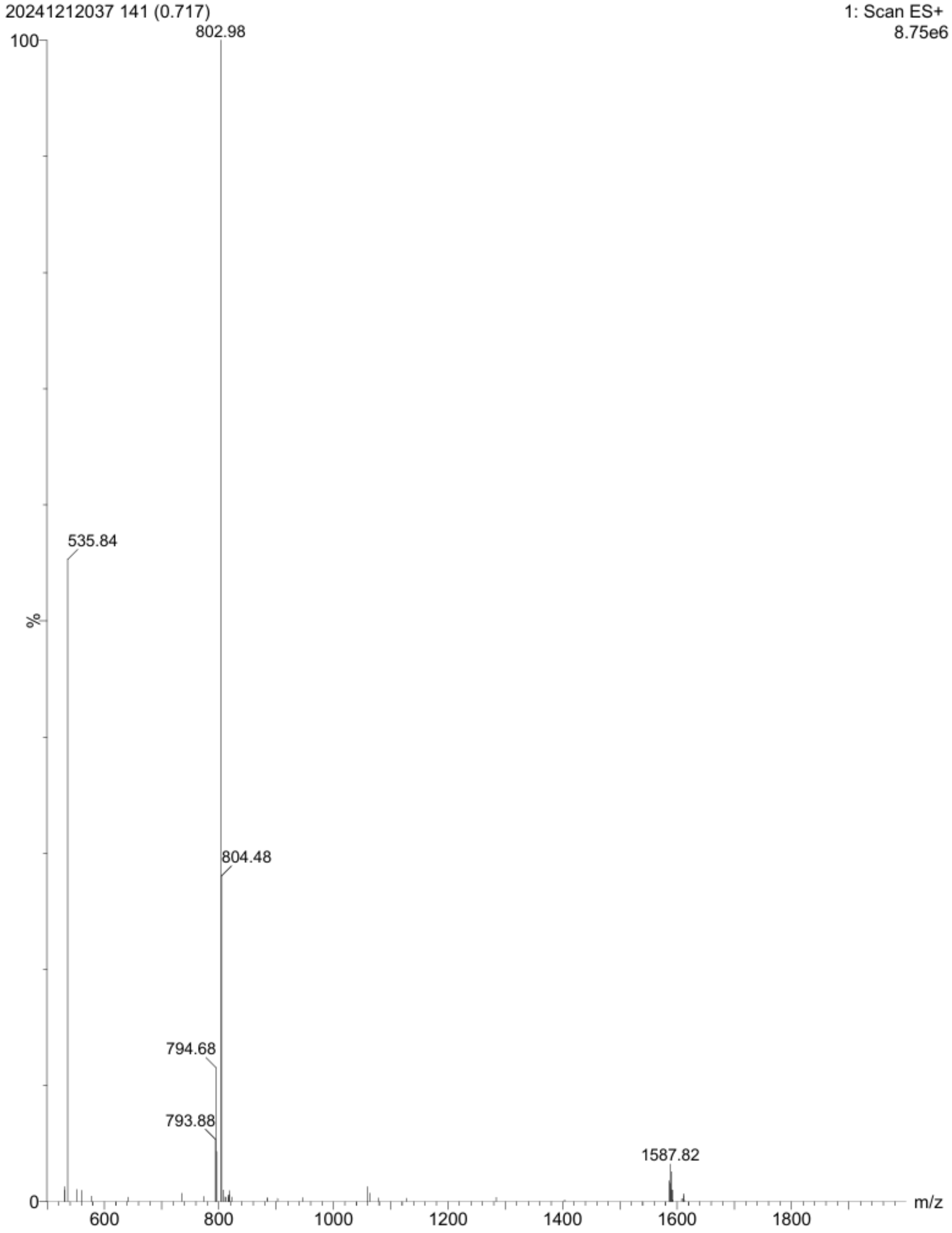

Figure S10.

The MS of ICG-Crizotinib.

Qualitative Analysis Report

**Data Filename**	ESIH202503827.d	**Sample Name**	F9-14911-34
**Sample ID**		**Position**	P1-A4
**Instrument Name**	Agilent 6520 Q-TOF	**User Name**	
**Acq Method**	20160322_MS_ESIH_POS_1min.m	**Acquired Time**	7/11/2025 2:39:00 PM
**IRM Calibration Status**	Success	**DA Method**	ESI-HR-20231114.m
**Comment**	ESIH by fangsu		

**Sample Group**		**Info.**	
**Data Filename**		**Stream Name**	LC 1
**Acquisition SW**	6200 series TOF/6500 series		
**Version**	Q-TOF B.08.00 (B8058.3 SP1)		

User Spectra

Fragmentor Voltage	Collision Energy	Ionization Mode
170	0	ESI

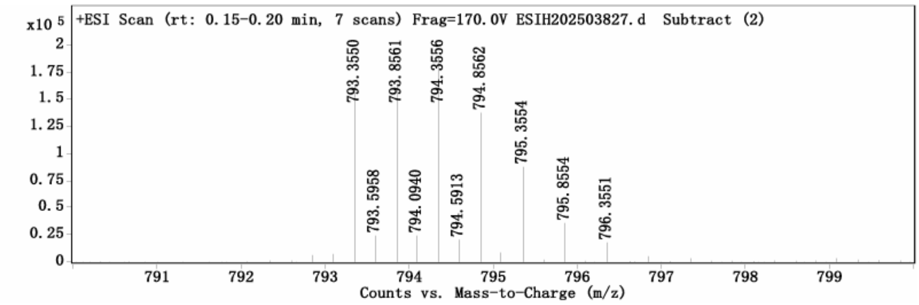

Figure S11.

The HRMS of ICG-Crizotinib.

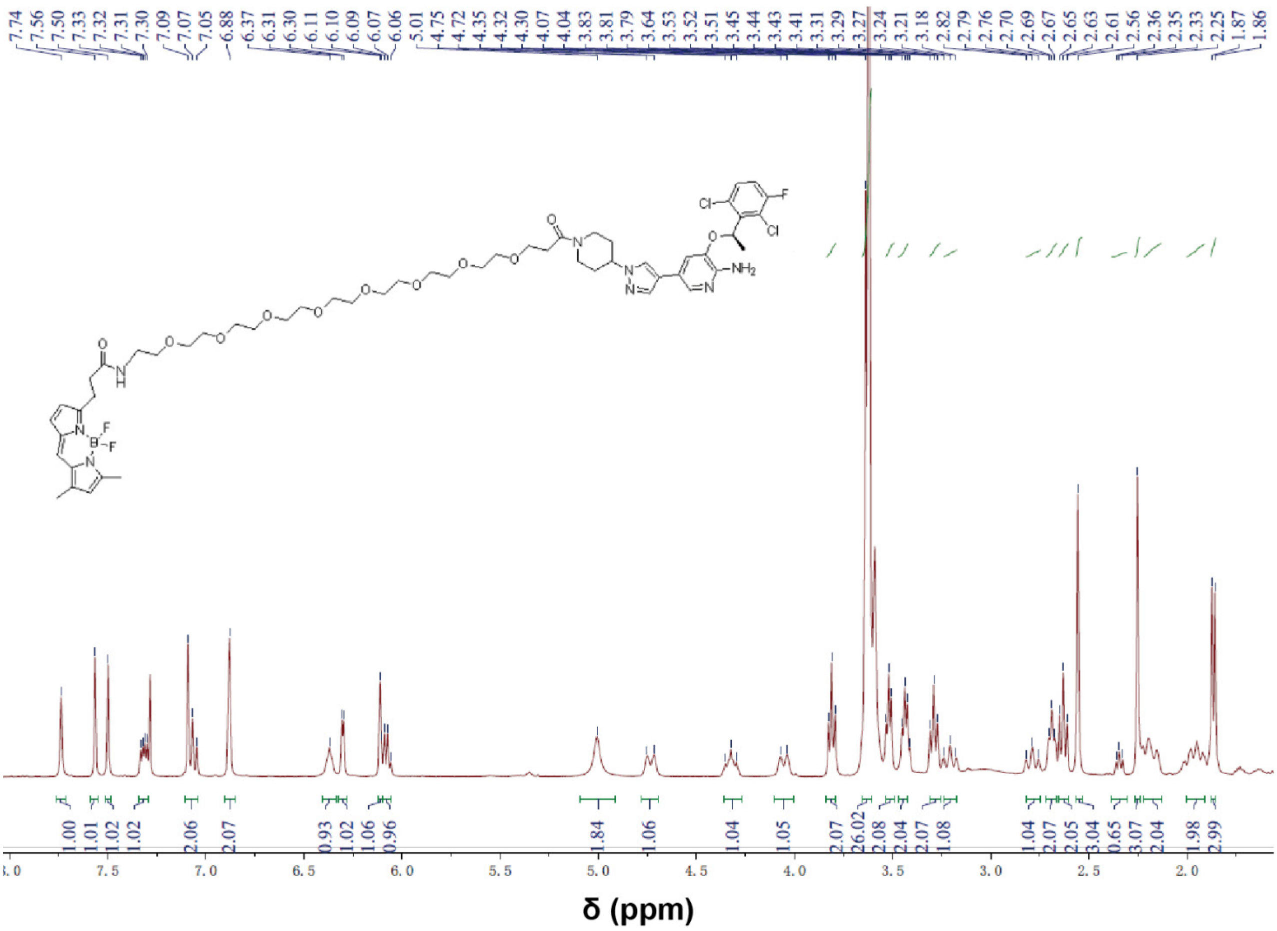

Figure S12.

The ^{1}H-NMR (400 MHz, Chloroform-d) of BODIPY-Crizotinib.

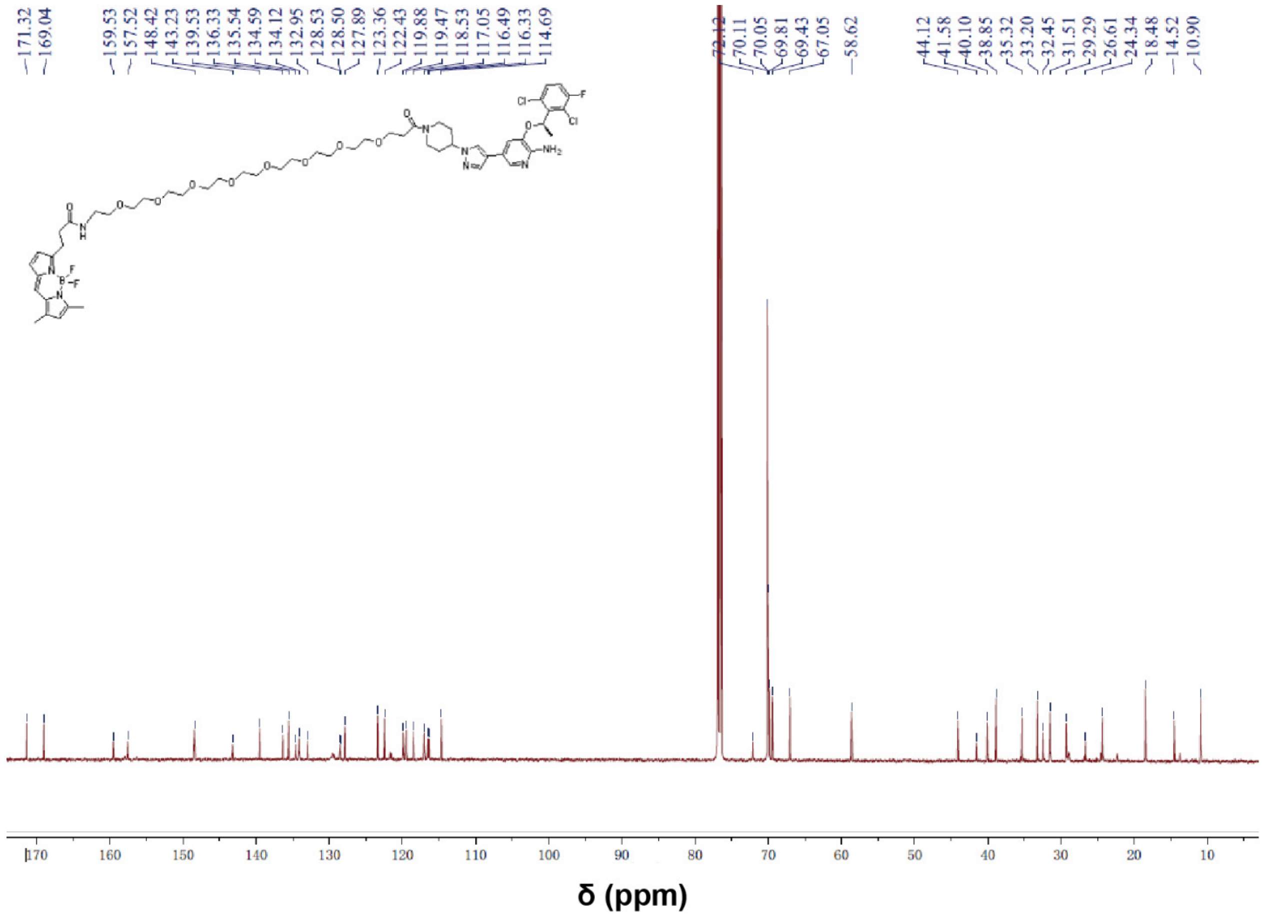

Figure S13.

The ^{13}C-NMR (151 MHz, $CDCl_3$) of BODIPY-Crizotinib.

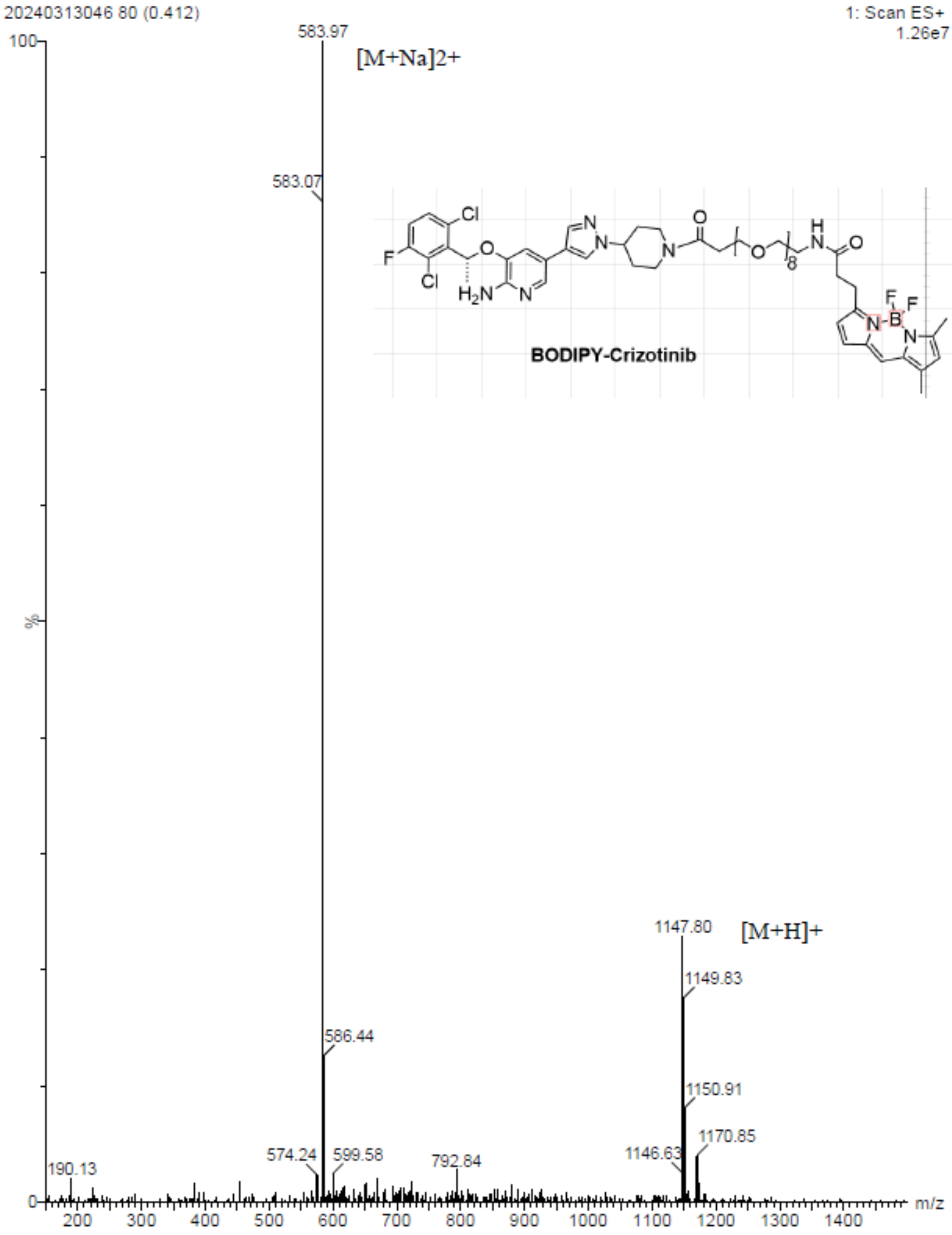

Figure S14.

The MS of of BODIPY-Crizotinib.

Supplementary Figures

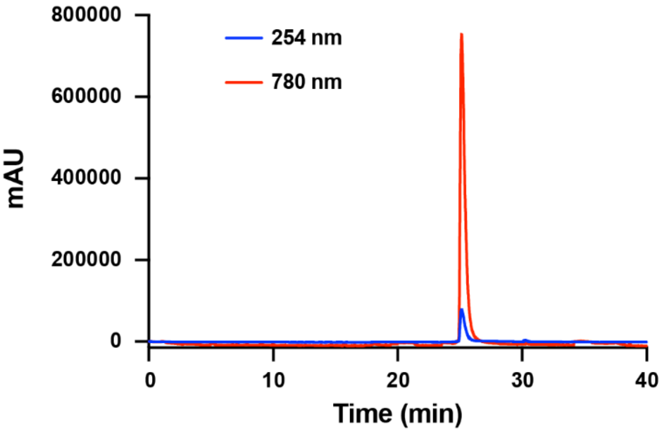

Figure S15.

HPLC analysis of IR788-Crizotinib. The purity of IR788-Crizotinib was confirmed by HPLC to be >99%.

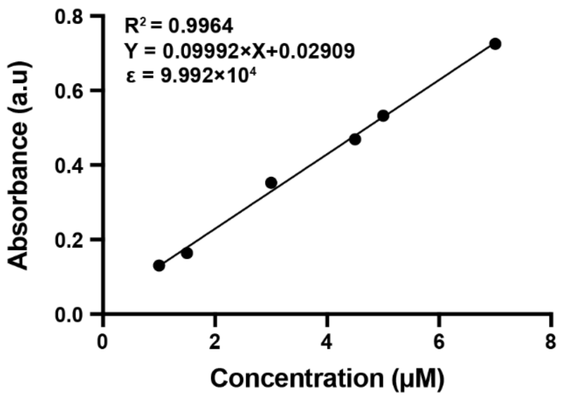

Figure S16.

Determination of the molar extinction coefficient of IR788-Crizotinib. Optical density of IR788-Crizotinib at 788 nm plotted as a function of probe concentration. The molar extinction coefficient, determined from the slope of the linear fit, was 9.992×10^4 $M^{-1}cm^{-1}$.

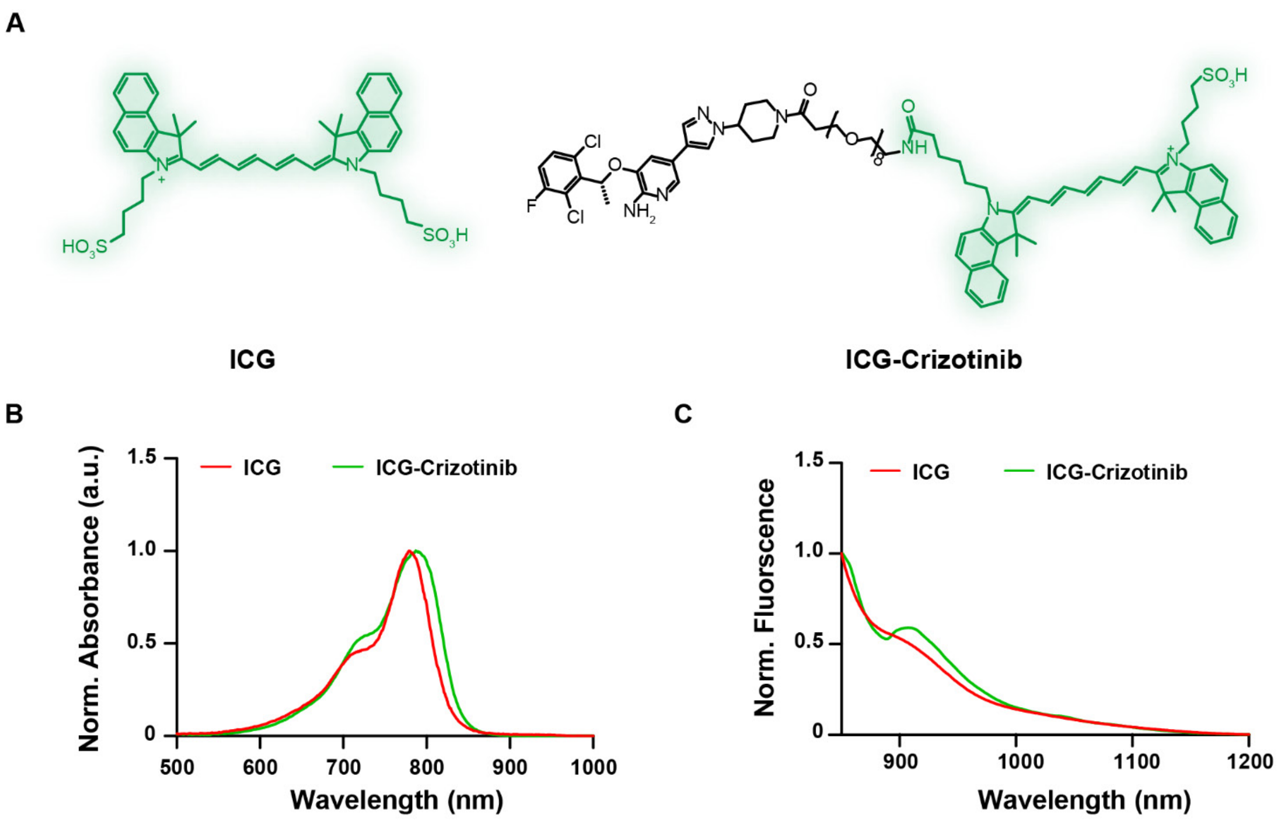

Figure S17.

Chemical structures and optical characterization of ICG and ICG-Crizotinib. (A) Chemical structures of ICG and ICG-Crizotinib. **(B)** Absorption spectra of ICG and ICG-Crizotinib. **(C)** Normalized fluorescence emission spectra of ICG and ICG-Crizotinib in the NIR-II region.

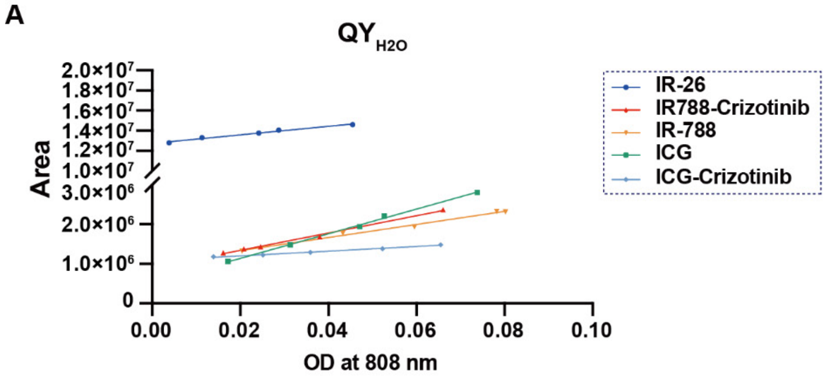

B

Probe	QY_{H2O}
IR-26 (Reference)	0.05%
IR788-Crizotinib	0.22%
IR788	0.17%
ICG	0.31%
ICG-Crizotinib	0.06%

Figure S18.

Determination of the fluorescence quantum yields of IR788-Crizotinib, ICG-Crizotinib, IR788, and ICG using IR-26 as the reference. (A) Linear fitting of integrated fluorescence intensity versus absorbance at 808 nm for IR-26, IR788-Crizotinib, IR788, ICG, and ICG-Crizotinib. For all samples, the absorbance at 808 nm was maintained below 0.1. **(B)** Calculated fluorescence quantum yields of the indicated dyes and probes in water. The quantum yields of IR788-Crizotinib, IR788, ICG, and ICG-Crizotinib were determined to be 0.22%, 0.17%, 0.31%, and 0.06%, respectively.

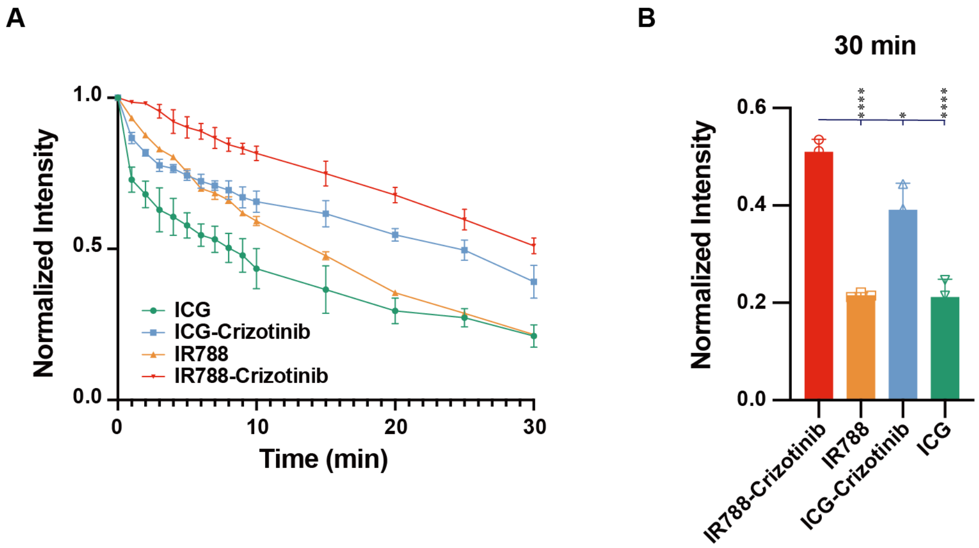

Figure S19.

Photostability of IR788-Crizotinib, ICG-Crizotinib, IR788, and ICG under continuous 808-nm laser irradiation. (A) Time-dependent normalized fluorescence intensity of IR788-Crizotinib, IR788, ICG, and ICG-Crizotinib under continuous 808-nm laser irradiation (75.5 mW cm^{-2}) for 30 min. Fluorescence signals were recorded in real time using an InGaAs short-wave infrared camera equipped with a 1000-nm long-pass emission filter and normalized to the initial intensity. **(B)** Quantitative comparison of normalized fluorescence intensity at 30 min (*$p < 0.05$, **** $p < 0.0001$; one-way ANOVA followed by Tukey's HSD test).

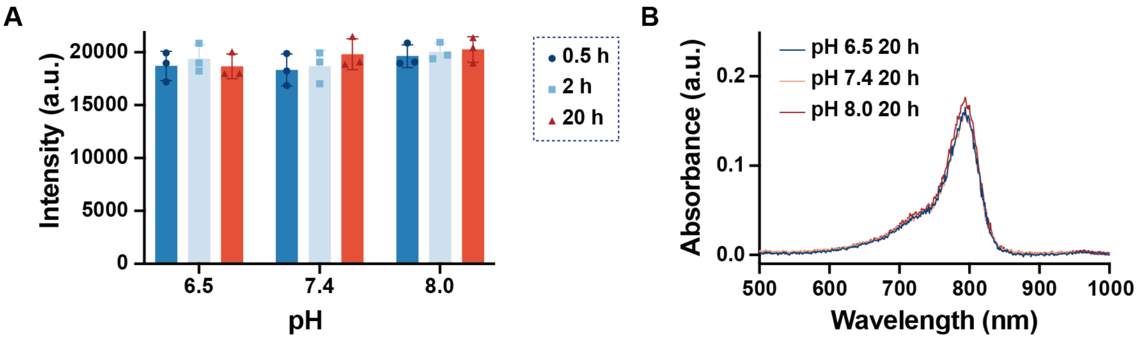

Figure S20.

Optical stability of IR788-Crizotinib in different pH. (A) Fluorescence intensity of IR788-Crizotinib after incubation at pH 6.5, 7.4, and 8.0 for 0.5 h, 2 h, and 20 h. **(B)** Absorption spectra of IR788-Crizotinib after 20 h incubation at pH 6.5, 7.4, and 8.0. No obvious shift in the absorption maximum or substantial spectral distortion was observed across the tested pH conditions.

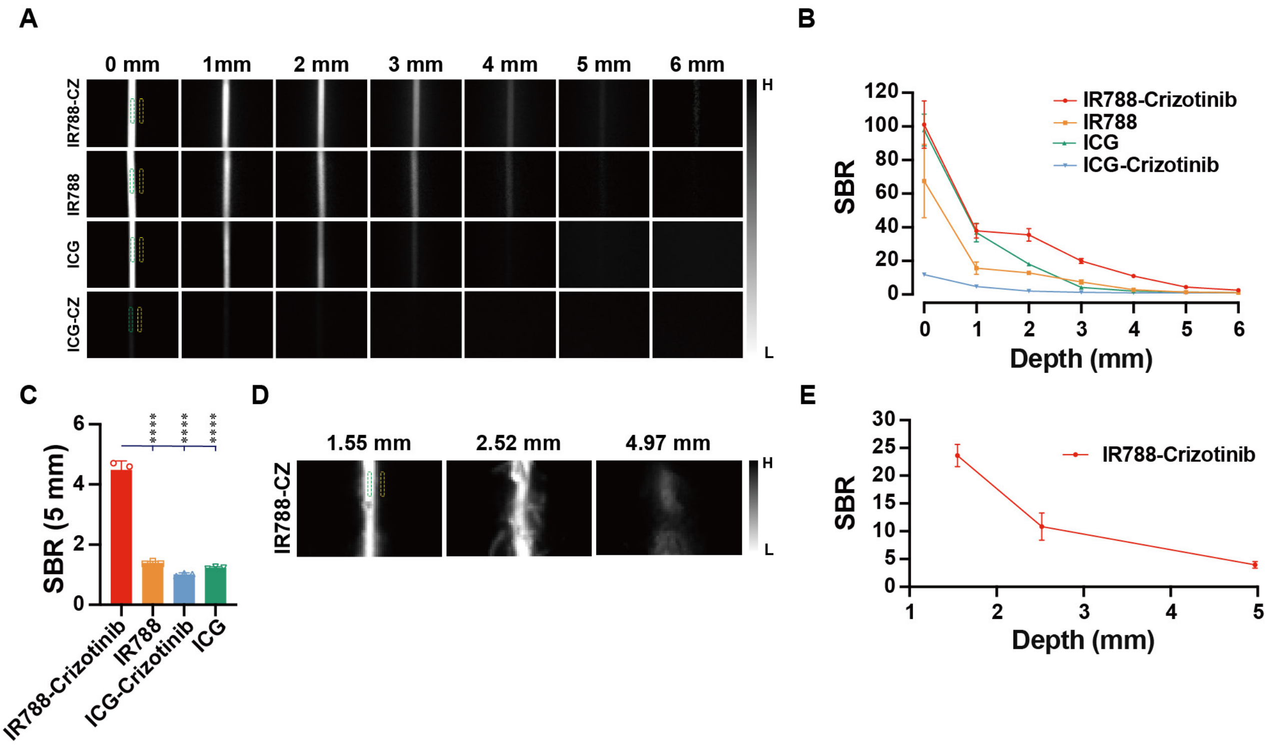

Figure S21.

In vitro* and *in vivo* penetration performance of IR788-Crizotinib, ICG-Crizotinib, IR788, and ICG. (A)** Representative NIR-II fluorescence images of capillaries filled with IR788-Crizotinib, IR788, ICG, or ICG-Crizotinib (100 µM in 1 × PBS) at increasing depths (0-6 mm) in 1% Intralipid. The green dashed outlines indicate the ROI used for capillary fluorescence signal measurement, and the yellow dashed outlines indicate the ROI used for background fluorescence measurement. **(B)** Quantitative analysis of signal-to-background ratio (SBR) as a function of phantom depth *in vitro*. **(C)** Quantitative comparison of SBR for the four probes at a depth of 5 mm in the *in vitro* penetration assay (* $p < 0.0001$; one-way ANOVA followed by Tukey's HSD test). **(D)** Representative NIR-II fluorescence images of capillary tubes filled with IR788-Crizotinib placed at different tissue depths in mice (1.55, 2.52, and 4.97 mm). The green dashed outlines indicate the ROI used for capillary fluorescence signal measurement, and the yellow dashed outlines indicate the ROI used for background fluorescence measurement. **(E)** Quantitative analysis of SBR as a function of tissue depth *in vivo*. CZ, Crizotinib; SBR, signal-to-background ratio.

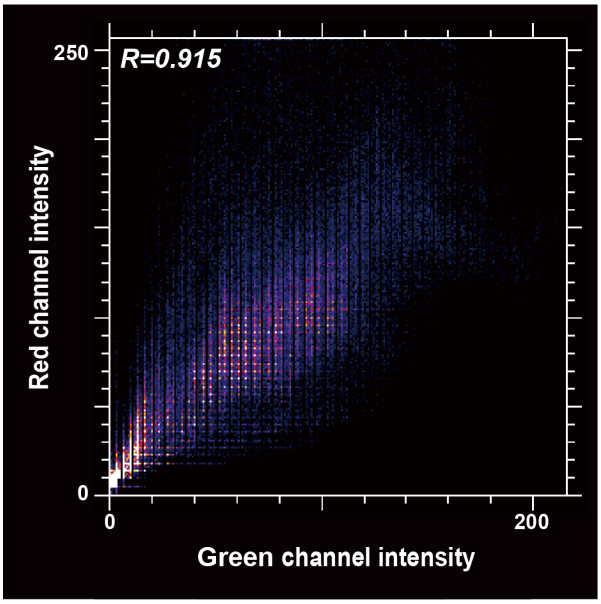

Figure S22.

Colocalization analysis of BODIPY-Crizotinib and c-Met immunofluorescence in Cal27-Luc cells. Two-color confocal microscopy showed strong spatial colocalization between BODIPY-Crizotinib and Cy5-labeled anti-c-Met immunofluorescence in Cal27-Luc cells. Quantitative colocalization analysis showed a Pearson correlation coefficient of 0.915.

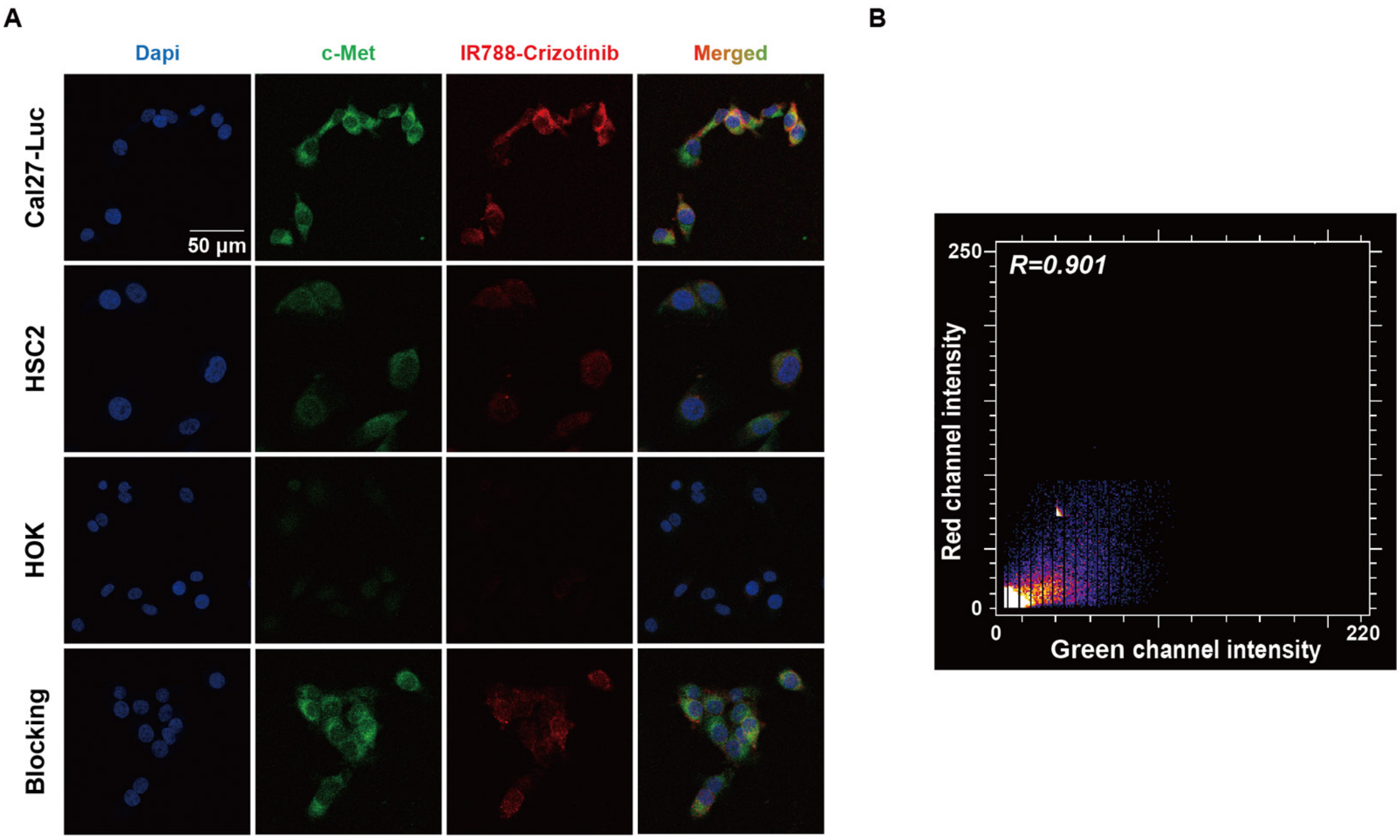

Figure S23.

***In vitro* confocal validation of IR788-Crizotinib targeting. (A)** Representative confocal images of Cal27-Luc, HSC2, HOK, and blocking-group cells co-stained with IR788-Crizotinib and anti-c-Met antibody. **(B)** Colocalization analysis in Cal27-Luc cells showed a correlation coefficient of 0.901 between IR788-Crizotinib fluorescence and c-Met immunofluorescence. Scale bar, 50 μm.

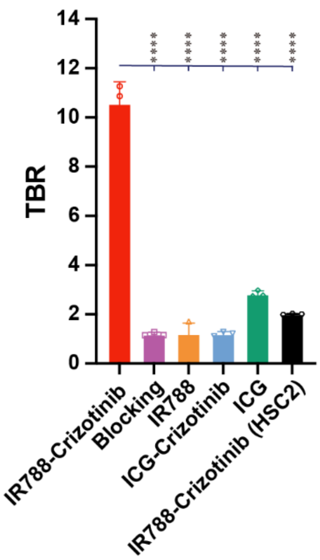

Figure S24.

Statistical comparison of tumor-to-background ratio (TBR) at 24 h post-injection. Bar graph summarizing the 24 h TBR values for each group derived from the time-course analysis shown in **Figure 4C.** Statistical significance was assessed by one-way ANOVA followed by Tukey's HSD multiple-comparison test.

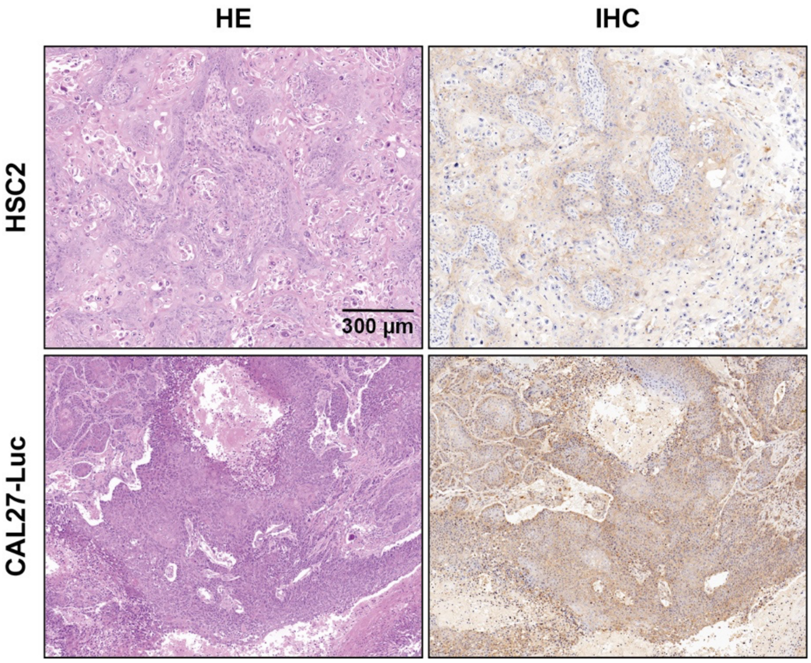

Figure S25.

Histopathological and c-Met immunohistochemical analysis of OSCC xenograft tumors. Representative H&E and c-Met immunohistochemical (IHC) staining of OSCC xenograft tumors used for *in vivo* imaging analysis. Scale bars, 300 μm.

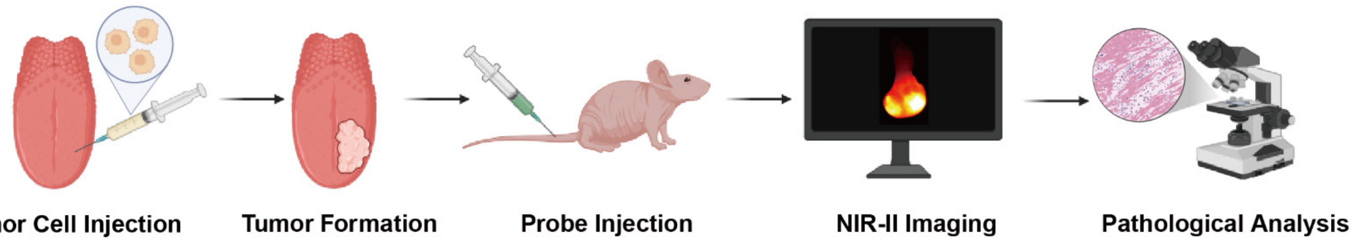

Figure S26.

Workflow for orthotopic OSCC establishment and *ex vivo* NIR-II fluorescence imaging. An orthotopic oral squamous cell carcinoma (OSCC) model was established by injecting Cal27-Luc cells into the tongue. Tongues were collected at the indicated time points after intravenous probe administration for *ex vivo* NIR-II fluorescence imaging. After imaging, the tongue tissues were processed for histopathological analysis to assess tumor distribution and delineate tumor boundaries. Created in BioRender. Ma, Q. (2026) https://BioRender.com/mo39jjh. OSCC, oral squamous cell carcinoma.

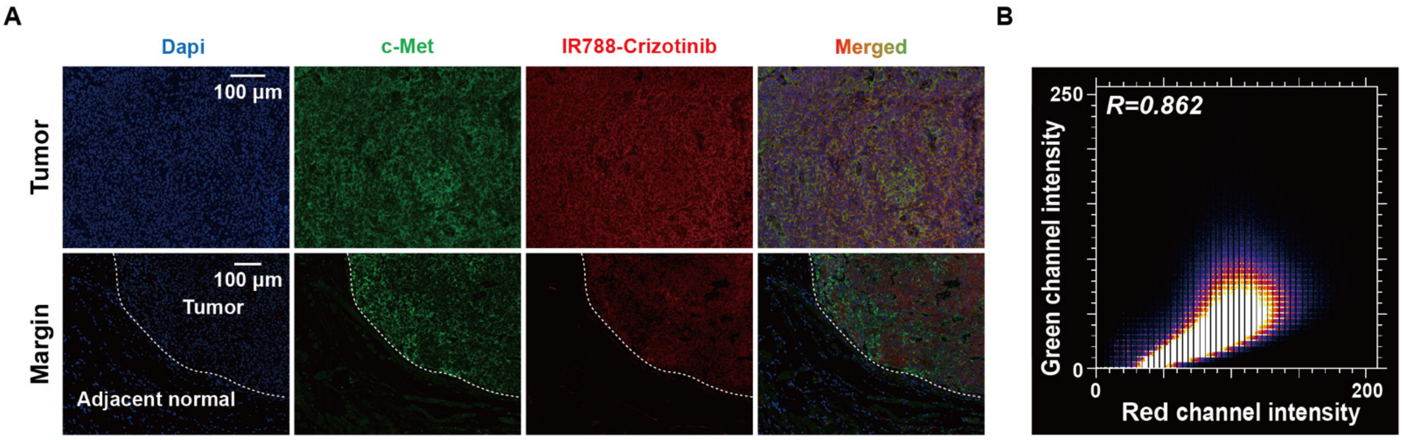

Figure S27.

Confocal fluorescence analysis of IR788-Crizotinib and c-Met in mouse tongue tumor sections. (A) Representative confocal images of DAPI (blue), c-Met immunofluorescence (green), IR788-Crizotinib (red), and merged images in the tumor region (upper row) and tumor margin region (lower row). The dashed line marks the boundary between tumor tissue and adjacent normal tissue in the margin images. **(B)** Correlation analysis of IR788-Crizotinib and c-Met signals in the tumor region. The Pearson's correlation coefficient was 0.862. Scale bars, 100 µm.

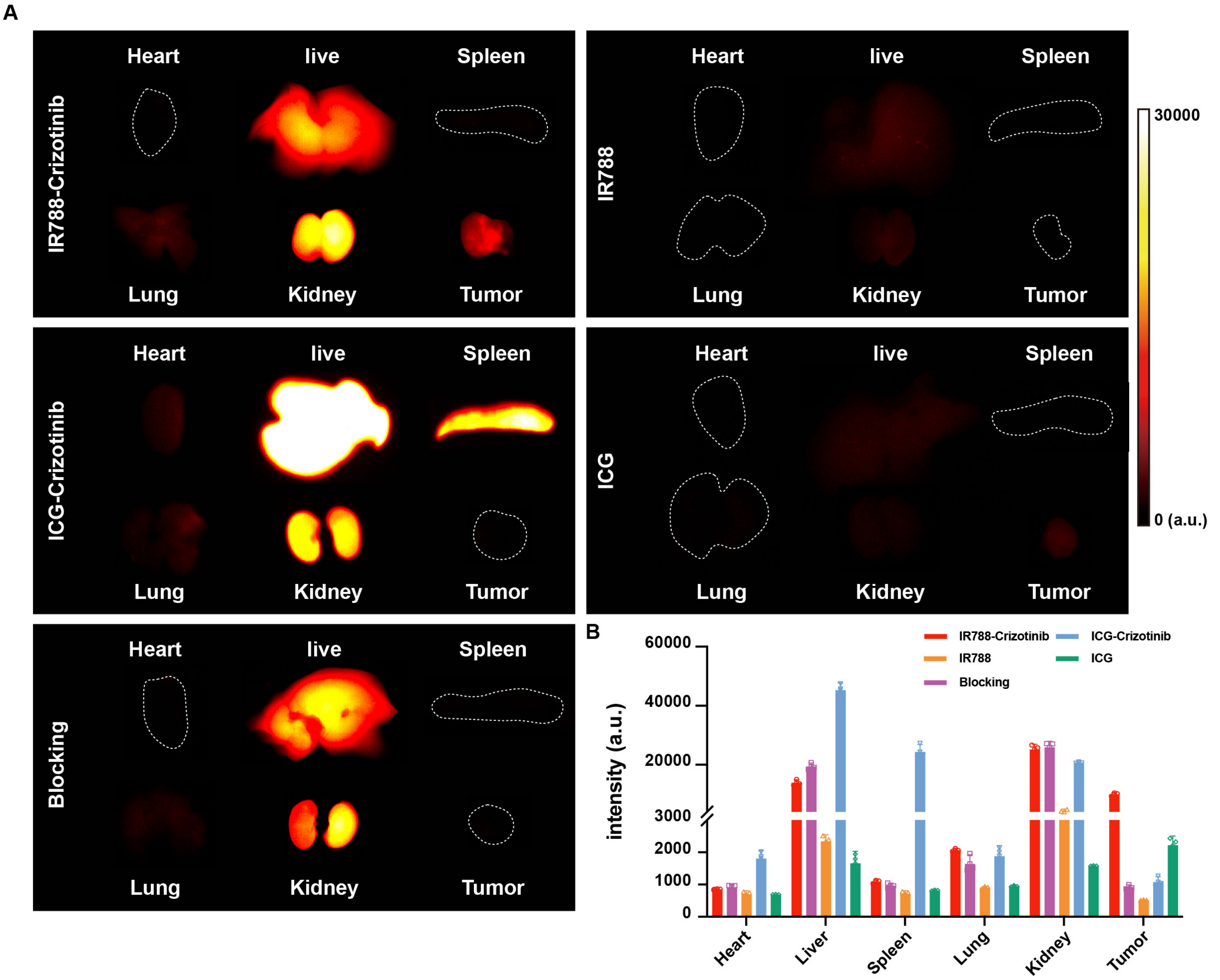

Figure S28.

***Ex vivo* probe biodistribution at 24 h post-injection. (A)** Representative *ex vivo* NIR-II fluorescence images of the heart, liver, spleen, lung, kidney, and tumor obtained 24 h after injection of the indicated probes. **(B)** Quantitative comparison of NIR-II fluorescence intensity in the major organs and tumor across groups.

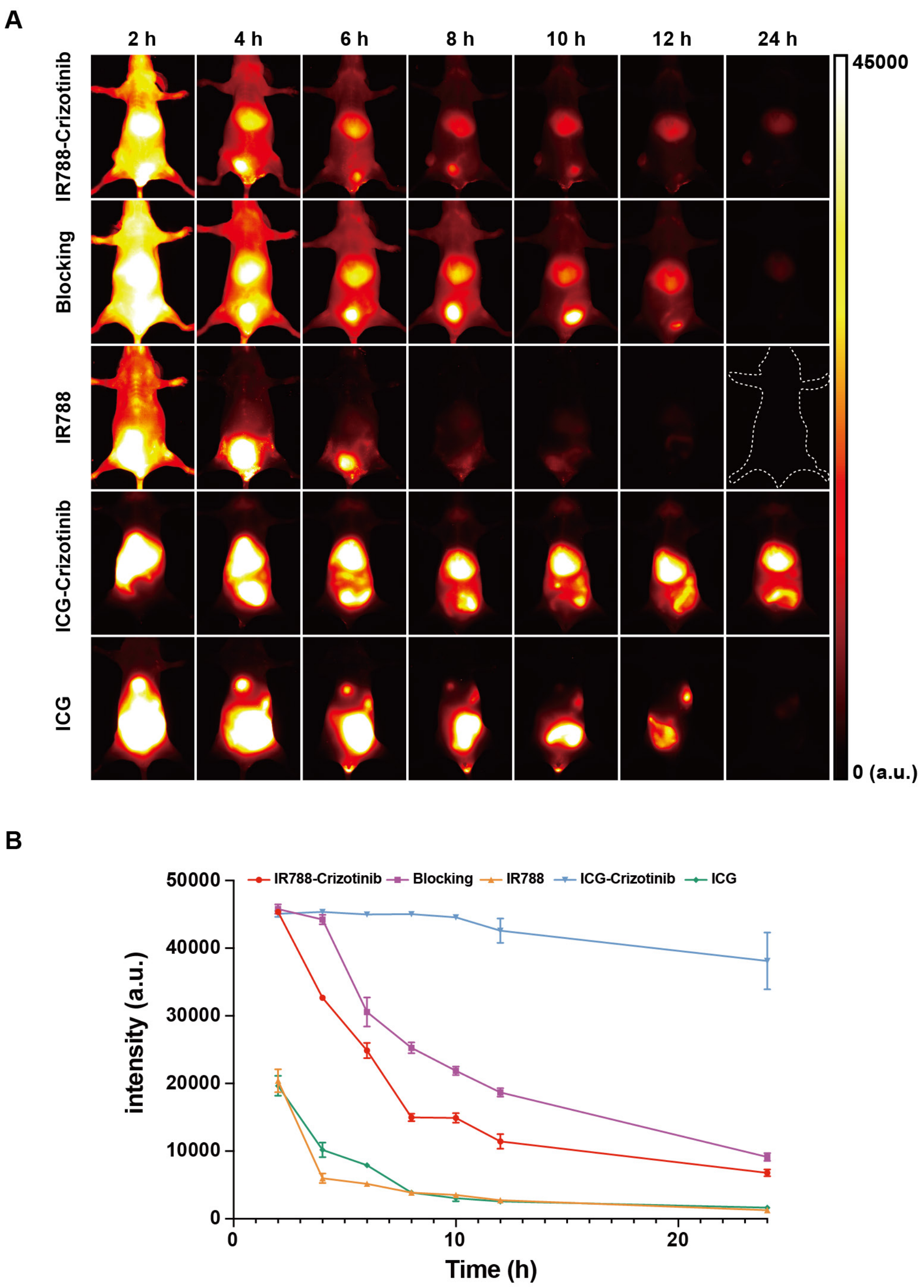

Figure S29.

Whole-body NIR-II fluorescence imaging in the supine position and quantification of hepatic fluorescence signals. (A) Representative whole-body NIR-II fluorescence images acquired in the supine position at the indicated time points after intravenous administration of the indicated probes. **(B)** Time-course quantification of hepatic fluorescence intensity for each group.

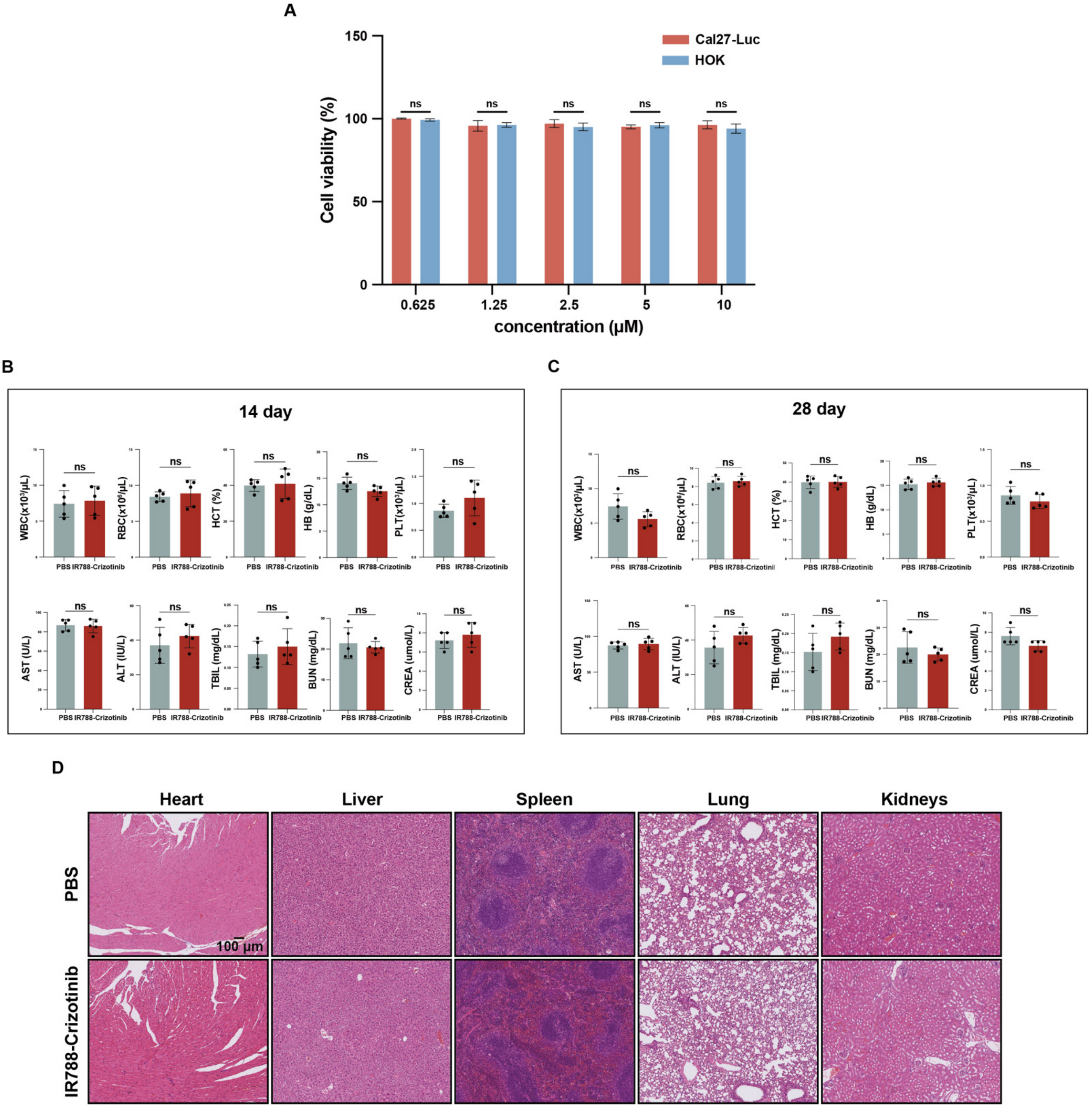

Figure S30.

***In vitro* and *in vivo* toxicity evaluation of IR788-Crizotinib. (A)** *In vitro* cytotoxicity assay in Cal27 and HOK cells. No significant difference in cell viability was observed between the two groups (ns, $p > 0.05$; Student's *t*-test). **(B and C)** Serum biochemical analysis at 14 and 28 days after probe administration. No significant differences were observed between the IR788-Crizotinib and PBS groups in hematological parameters or indices of hepatic and renal function (n = 5, 15 μg/g; ns, $p > 0.05$; Student's *t*-test). **(D)** Representative H&E staining of major organs (heart, liver, spleen, lung, and kidney) collected at 28 days after administration, showing no evident histopathological abnormalities. ns, not significant.

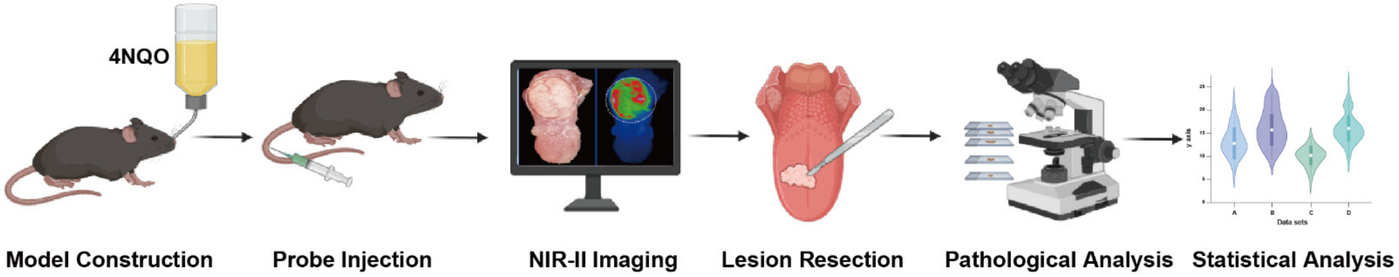

Figure S31.

Workflow of NIR-II imaging-guided resection in the 4-NQO-induced oral lesion model. A stepwise oral carcinogenesis model was established by administering 4-nitroquinoline-1-oxide (4-NQO) in the drinking water to induce graded oral epithelial dysplasia and oral squamous cell carcinoma (OSCC). After intravenous administration of the probe, NIR-II fluorescence imaging was performed at 24 h post-injection, and regions with abnormally increased fluorescence were resected under image guidance. The resected tissues were subsequently subjected to histopathological analysis and quantitative evaluation. Created in BioRender. Ma, Q. (2026) https://BioRender.com/uecfupl. 4-NQO, 4-nitroquinoline-1-oxide; OSCC, oral squamous cell carcinoma.

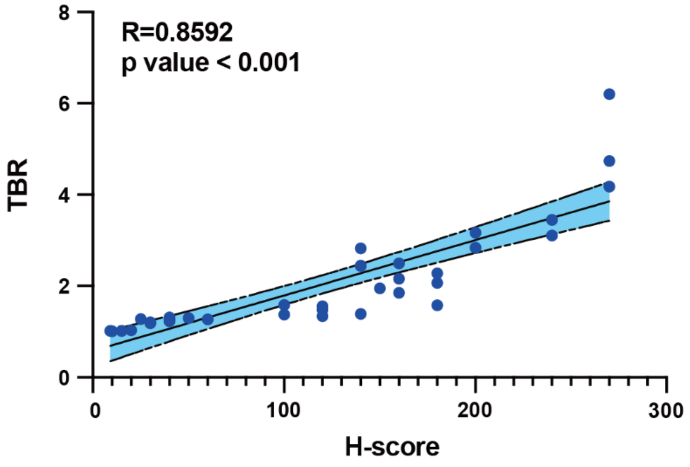

Figure S32.

Correlation between c-Met IHC H-score in biopsy specimens and tumor-to-background ratio (TBR) in NIR-II imaging. Scatter plot showing the relationship between TBR and c-Met IHC H-score (0-300) in biopsy specimens. The solid line denotes the least-squares linear regression fit, and the shaded area represents the 95% confidence interval (Pearson r = 0.859, two-sided $p < 0.001$). Each point represents an independent sample. TBR, tumor-to-background ratio.

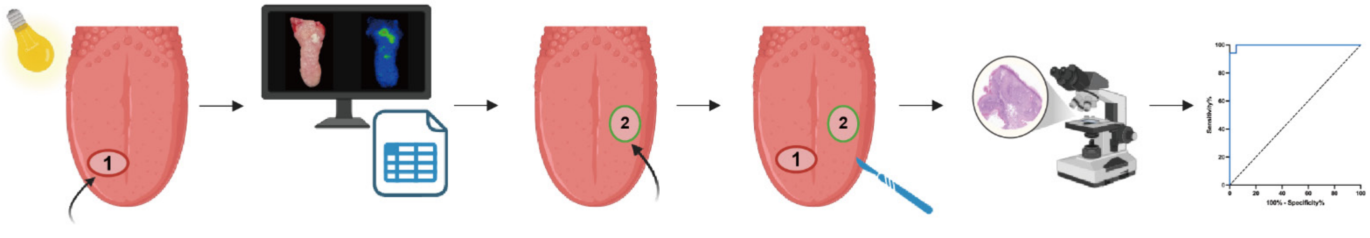

Figure S33.

Workflow comparing NIR-II fluorescence-guided biopsy with conventional white-light selection. Suspicious areas for high-risk oral lesions were first identified under white-light (WL) imaging and then reassessed by NIR-II fluorescence (FL) imaging using a prespecified tumor-to-background ratio (TBR) threshold. Regions identified as suspicious by either modality were subjected to biopsy. Histopathology was used as the reference standard, and the diagnostic performance of WL-guided and NIR-II-guided biopsy-site selection was quantified and compared. Created in BioRender. Ma, Q. (2026) https://BioRender.com/54rh88e. WL, white-light; FL, fluorescence; TBR, tumor-to-background ratio.

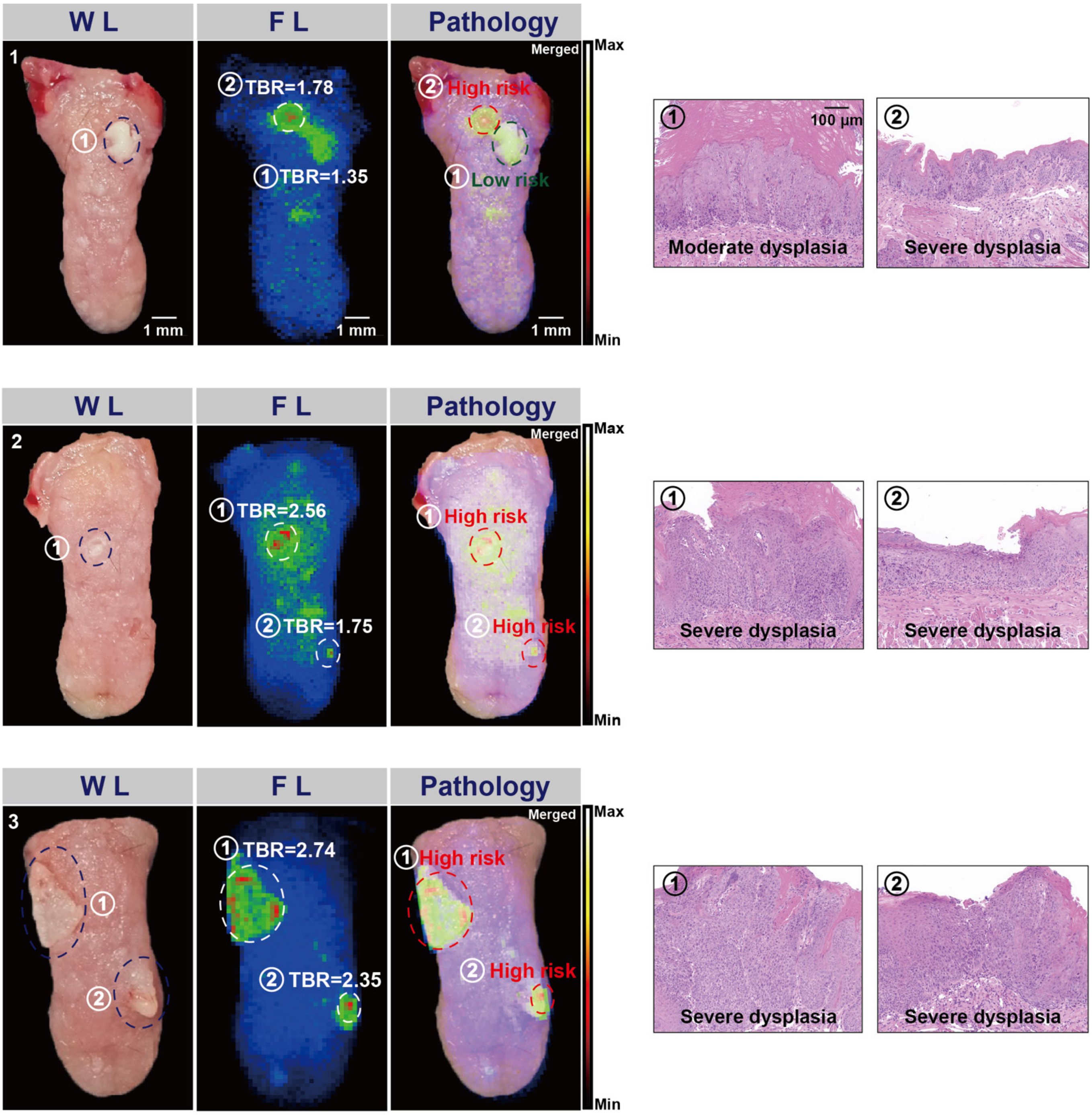

Figure S34.

White-light imaging, fluorescence imaging, and H&E staining of the biopsy specimens shown in Figure 5F. Representative white-light (WL) images, fluorescence (FL) images, and corresponding H&E-stained sections of the biopsy specimens shown in Figure 5F. The right-hand panels display the histopathological results corresponding to each biopsy specimen. Scale bars: 1 mm (left panels) and 100 μm (right panels).

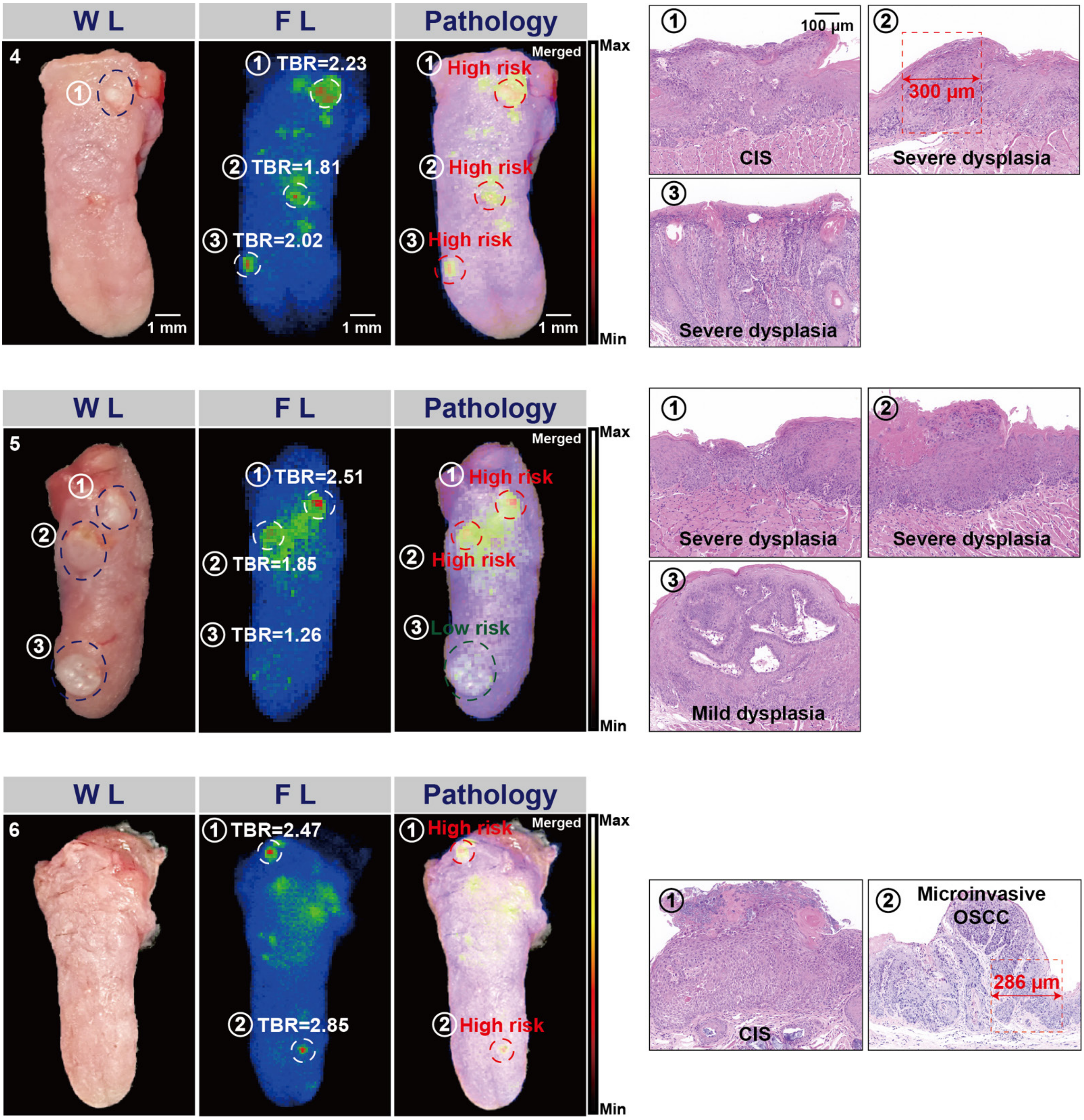

Figure S35.

White-light imaging, fluorescence imaging, and H&E staining of the biopsy specimens shown in Figure 5F. Representative white-light (WL) images, fluorescence (FL) images, and corresponding H&E-stained sections of the biopsy specimens shown in Figure 5F. The right-hand panels show the corresponding histopathological findings. Dashed red boxes delineate the minimal regions detected for severe dysplasia and microinvasive oral squamous cell carcinoma (OSCC), respectively. Scale bars: 1 mm (left panels) and 100 μm (right panels). OSCC, oral squamous cell carcinoma; CIS, carcinoma in situ.

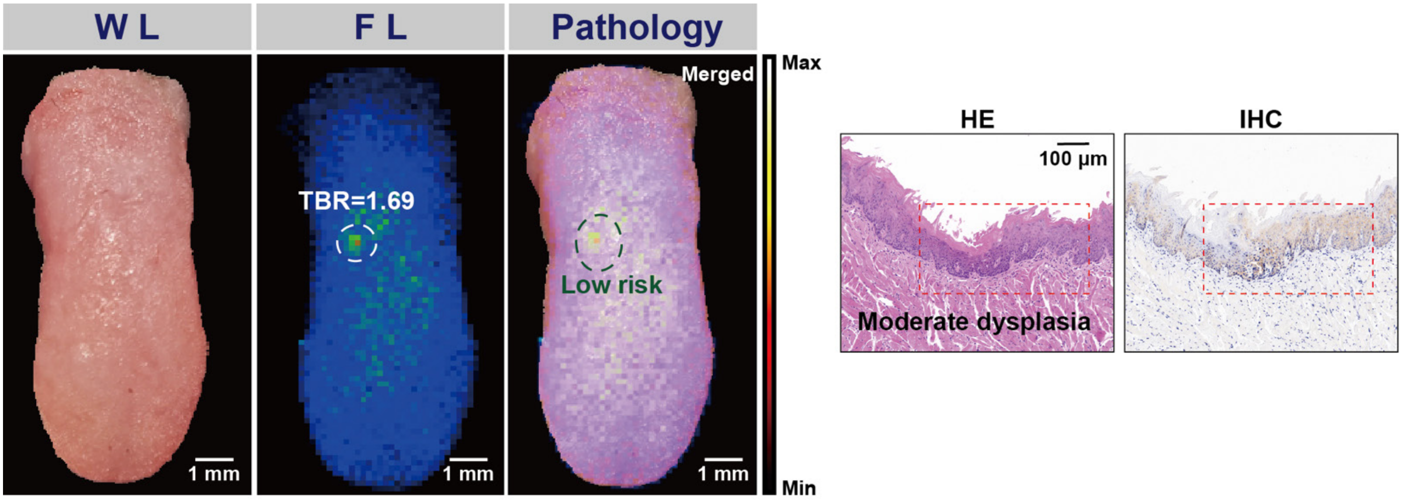

Figure S36.

Representative case of fluorescence-based false-positive classification. Under white-light (WL) endoscopy, no suspicious high-risk lesion was selected. Reassessment by NIR-II fluorescence (FL) imaging identified a suspicious region (white dashed box) with a tumor-to-background ratio (TBR) of 1.69, exceeding the prespecified threshold of 1.57. Histopathological analysis confirmed moderate epithelial dysplasia. The corresponding H&E sections and c-Met IHC are shown in the right-hand panels. The red dashed box delineates the area of moderate dysplasia. Scale bars: 1 mm (left panels) and 100 μm (right panels). WL, white-light; FL, fluorescence; TBR, tumor-to-background ratio.

.

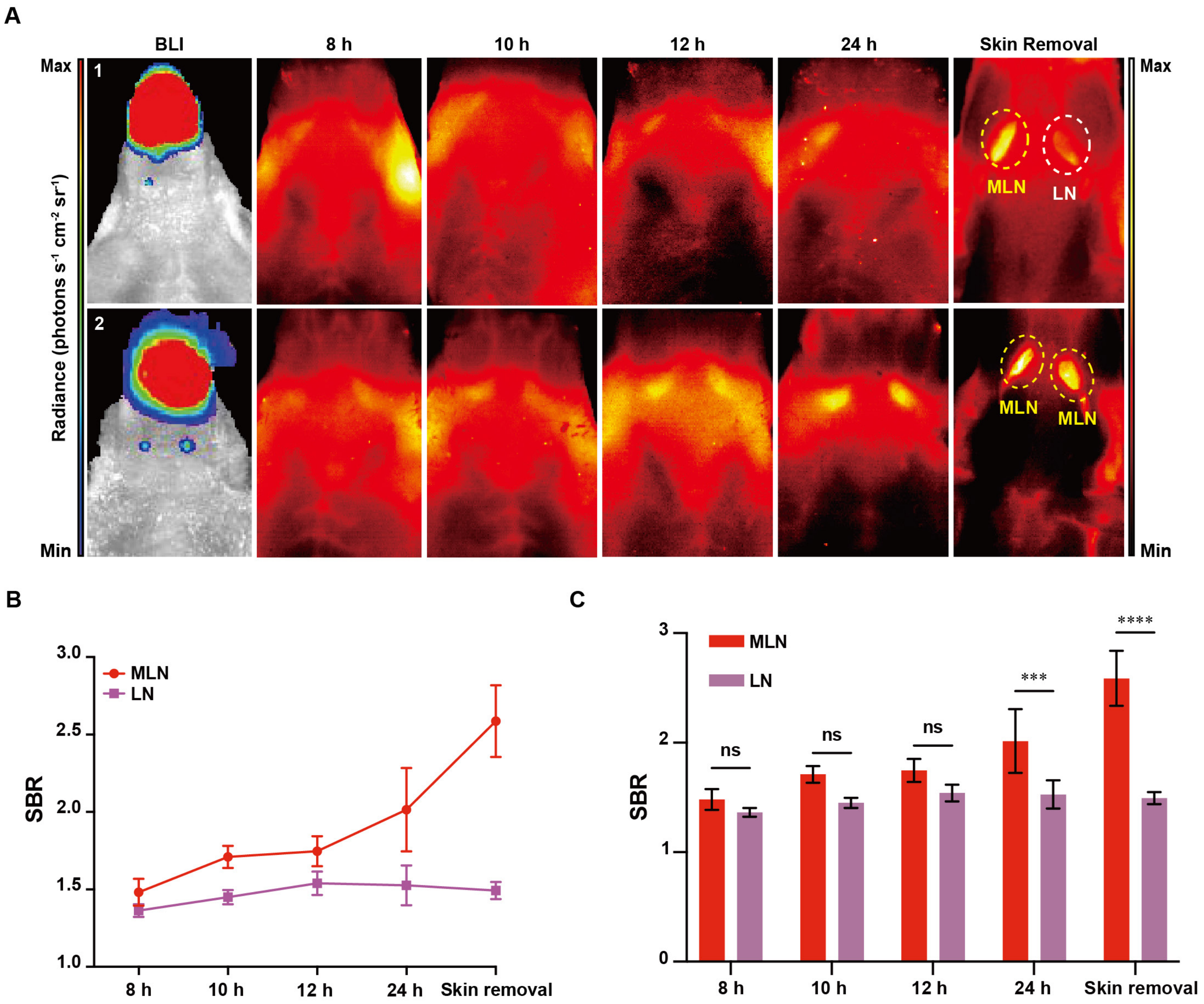

Figure S37.

NIR-II fluorescence imaging and quantification of cervical lymph node metastasis in an OSCC nude mouse model. (A) Representative bioluminescence (IVIS) and NIR-II fluorescence images acquired at 8, 10, 12, and 24 h after probe administration, and after skin removal, in Cal27-Luc orthotopic OSCC mice with cervical lymph node metastasis. **(B and C)** Quantitative comparison of signal-to-background ratio (SBR) between normal lymph nodes (LN) and metastatic lymph nodes (MLN) at the indicated time points (ns, $p > 0.05$; *** $p < 0.001$; **** $p < 0.0001$; paired two-tailed *t*-test). SBR, signal-to-background ratio; MLN, metastatic lymph node; LN, normal lymph node; ns, not significant.

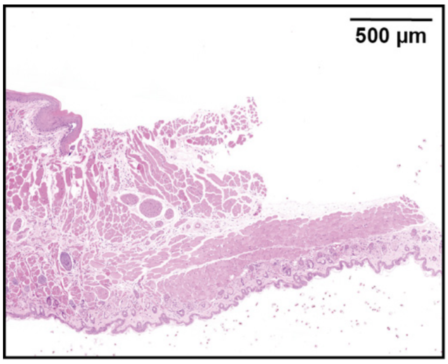

Figure S38.

Histopathological evaluation of the surgical bed after fluorescence-guided resection of floor-of-mouth squamous cell carcinoma. Representative H&E staining of the surgical bed after resection of the residual fluorescent focus identified in **Figure 7**. No residual tumor cells were identified in the examined section. Scale bar, 500 µm.

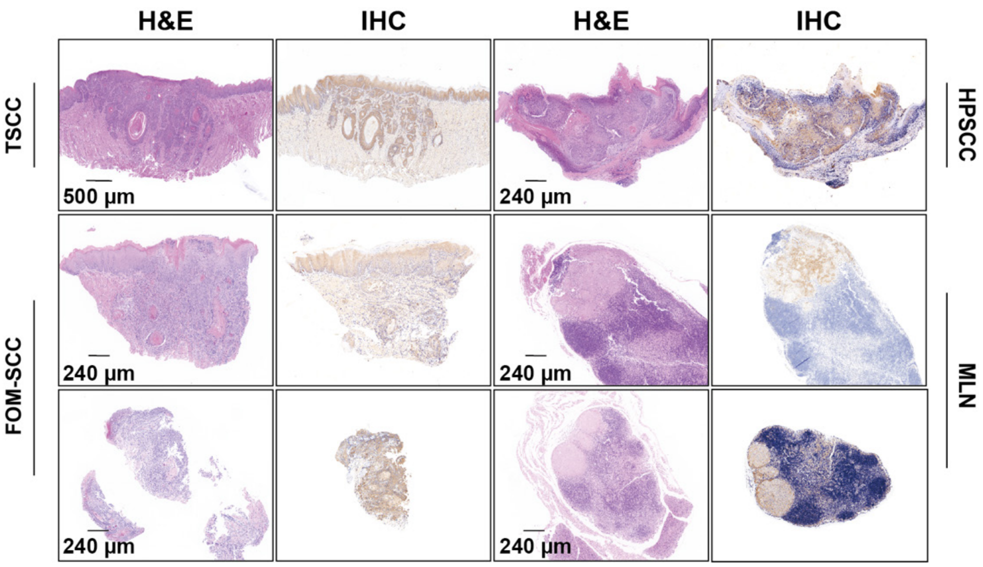

Figure S39.

Histopathological and c-Met immunohistochemical validation of the surgically resected specimens shown in Figure 7. Representative H&E and c-Met immunohistochemical (IHC) staining of surgically resected specimens corresponding to the fluorescence-guided surgery cases shown in **Figure 7**, including tongue squamous cell carcinoma (TSCC), hard palate squamous cell carcinoma (HPSCC), floor-of-mouth squamous cell carcinoma (FOM-SCC), and metastatic lymph nodes (MLN). Tumor-containing regions identified by H&E showed corresponding positive c-Met staining in paired IHC sections. Scale bars: 500 μm, 240 μm.
